# Photolyzable Polymer Brushes: Subtractive 3D Structuring of Surfaces Using Water and Light

**DOI:** 10.1002/anie.2790800

**Published:** 2026-04-13

**Authors:** Henrik Kalmer, Federica Sbordone, Phuong T. Do, Kai Mundsinger, Hazal Kayas, Robert T. Jones, Jayanti Mendhi, Tim R. Dargaville, Damien G. Harkin, Lukas Michalek, Andrew Nelson, Hendrik Frisch

**Affiliations:** ^1^ Centre for Materials Science Queensland University of Technology (QUT) Brisbane QLD Australia; ^2^ School of Chemistry and Physics Queensland University of Technology (QUT) Brisbane QLD Australia; ^3^ Central Analytical Research Facility (CARF) Queensland University of Technology (QUT) Brisbane QLD Australia; ^4^ School of Biomedical Sciences Queensland University of Technology (QUT) Brisbane QLD Australia; ^5^ Centre for Vision and Eye Research Queensland University of Technology (QUT) Brisbane QLD Australia; ^6^ Department of Chemical Engineering Stanford University Stanford California USA; ^7^ Australian Nuclear Science and Technology Organisation (ANSTO) Kirrawee DC NSW Australia

**Keywords:** polymer brushes, photodegradation, SI‐PET‐RAFT

## Abstract

Polymer brushes are a key technology for designing surfaces, with applications in biomedicine alone including biosensing, cell culture, regenerative medicine, and antibacterial coatings. The structuring of polymer brushes has the potential to precisely tailor interfaces for specific application requirements. However, complex fabrication processes can limit the applications of polymer brushes. Herein, a subtractive patterning process is reported, which decouples initial fabrication from the structuring process. Using radical ring‐opening polymerization of cyclic monomers with photocleavable cyclobutane rings, photodegradable targets are directly embedded into the polymer brush main chains. After the initial fabrication, these brushes can be readily degraded with light, triggering photocleavage of the cyclobutane units. This enables continuous brush degradation of over 50% of brush height for topographical patterning without affecting brush properties such as hydrophilicity and adhesion force. The inherent photodegradability of the polymer brush eliminates the need for additional chemicals or catalysts and can be carried out using nothing but water and light at ambient temperature.

## Graphical abstract

Facile structuring of polymer brushes is achieved by flipping the process from controlled growth to controlled brush photolysis. Using surface‐initiated radical ring‐opening polymerization of cyclic monomers with photocleavable cyclobutane rings, photodegradable targets are embedded into the polymer chains. The enabled continuous subtractive patterning is decoupled from initial polymerization conditions, allowing precise structuring of polymer brushes with only water and light.

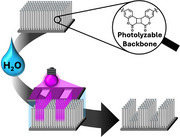

## 1 Introduction

The interactions between materials, whether natural or synthetic, are largely governed by their surfaces [1]. Polymer coatings, for instance, are used to tailor surface properties of materials to ensure the safety of medical devices [2, 3, 4], to prevent corrosion [5, 6, 7], or for reliable food packaging [4, 8, 9]. Driven by its wide range of applications, interest in surface science has long existed and continues to grow, with advances in vacuum technology, analytical techniques, and digital computing in the 1960s further accelerating research on surface structuring [10, 11, 12]. While many surface modifications rely on physisorption, the chemical modification of surfaces through *grafting‐to* and *grafting‐from* approaches offers control over the exact architecture of the surface layer by tethering polymers to the surface—especially if the polymers are packed densely enough to form a polymer brush [13, 14, 15, 16].

To exert control over polymer formation, light provides spatiotemporal precision, enabling selective growth precisely where and when desired [17, 18, 19]. The use of light to control polymerizations has been widely applied for a range of polymerization mechanisms [20, 21], including reversible deactivation radical polymerizations (RDRP) or ultraviolet induced atom‐transfer radical polymerization [22]. However, a major limitation of most light‐driven polymerization techniques is the sensitivity to molecular oxygen, which efficiently quenches the propagating radical species. This limitation was overcome by the work of Boyer through the development of oxygen‐tolerant photo‐induced electron/energy transfer reversible addition fragmentation chain transfer (PET‐RAFT) [23]. This technique paved the way to light‐gated polymerizations without extensive reaction setup, using light as an external stimulus to mediate the polymerization in open reaction vessels [23, 24]. While the initial PET‐RAFT technique relied on an iridium‐based photoredox catalyst [23], further developments led to the usage of other more affordable photocatalysts, such as zinc tetraphenylporphyrin (ZnTPP) [25, 26], zinc tetra‐4‐hydroxyphenylporphyrin (ZnHTPP) [27], and Eosin Y [28], among numerous others [25, 28, 29, 30].

**SCHEME 1:**
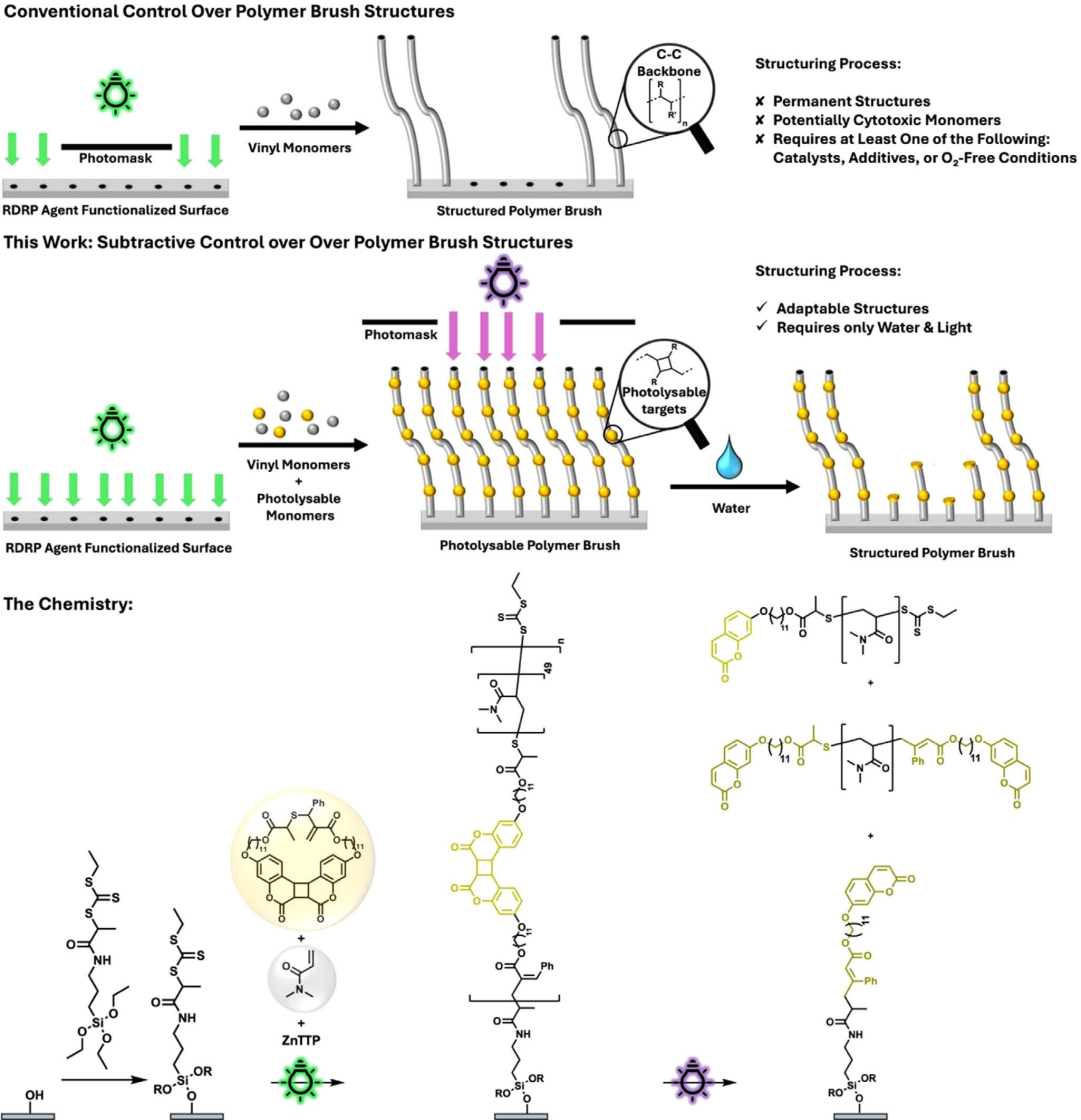
Conventional polymer brush structure control in the growth stage (top). This work controls polymer brush structure post‐growth, in a subtractive process under mild conditions without the use of organic solvents or catalysts (middle). Reaction scheme of the surface functionalization with a CTA, subsequent SI‐PET‐RAFT copolymerization with *N*,*N*‐Dimethylacrylamide (DMA) and cyclic allyl sulfide monomer, **M**, containing a photoreversible cyclobutane moiety that incorporates into the polymer main chains to render them photolyzable (bottom).

To obtain patterned polymer brushes, a chain transfer agent (CTA) is generally tethered to the surface to conduct PET‐RAFT polymerization selectively in the irradiated areas as gated by photomasks (Scheme 1, top) [31, 32, 33]. An improved control over the brush growth, by performing surface‐initiated PET‐RAFT (SI‐PET‐RAFT) in flow conditions, has been reported by Zuilhof and coworkers [34]. Pester and co‐workers, as well as other researchers [35], even achieved topologies of two different polymer brush heights by chain extending a polymer brush with a photomask [36]. Multilevel structuring of polymer brushes by performing multiple polymerization cycles with different monomers, thereby creating multi‐block copolymers, was also shown by the same team [37].

The development of catalyzed aqueous PET‐RAFT has allowed to overcome the limitation of organic solvents [38], but may require organic additives as sacrificial electron‐donor [39]. Catalyst‐free growth of polymer brushes has been achieved using surface‐initiated photoiniferter‐mediated polymerization (SI‐PIMP)‐based polymerizations [40], which are, however, sensitive to oxygen [41, 42]. The underpinning approach to structure polymer brushes by controlling the growth of the brush therefore generally comes with three requirements: (i) the process inherently requires monomers, which are often cytotoxic at the concentrations needed for polymerization (even when the resulting polymer is comparatively well tolerated); (ii) it requires at least one of the following—catalysts, organic additives, or deoxygenated/oxygen‐managed conditions—which may be incompatible with living systems; and (iii) the obtained brush structures are permanent and cannot be readily adjusted post‐fabrication.

Decoupling the structuring processes from the initial polymer brush growth holds promise of overcoming these challenges by using a subtractive approach. However, the all‐carbon backbones that result from the underpinning RDRP processes used for brush fabrication are notoriously inert. Polymer brush post‐fabrication modifications have therefore been limited to interchain crosslinking [16] or cleavage of the chemical moiety that tethers the polymer to a surface, leading to removal of the full chain [43, 44].

Here, we report a scalable synthesis of continuously photocleavable polymer brushes based on surface‐induced radical ring‐opening copolymerization of cyclic allylic sulfides (CAS) (Scheme 1, middle). CAS monomers and their derivatives have been traditionally used to embed hydrolytic targets into the otherwise all‐carbon backbone of polyvinyl polymers [45, 46, 47, 48, 49, 50], but have recently been shown to be able to insert more complex chemical structures, such as peptides [51, 52] or photochemical cleavage targets [53, 54]. By embedding photocleavable targets into the polymer chains of a brush, its degradation and three‐dimensional (3D) patterning can be performed continuously in a post‐fabrication stage—using nothing but light and a solvent such as water (Scheme 1, bottom). The introduced technology can be used to control 3D structures of polymer brushes due to continuous photolysis of the surface‐bound polymer chains, rather than a stepwise growth process.

## 2 Results and Discussion

**FIGURE 1:**
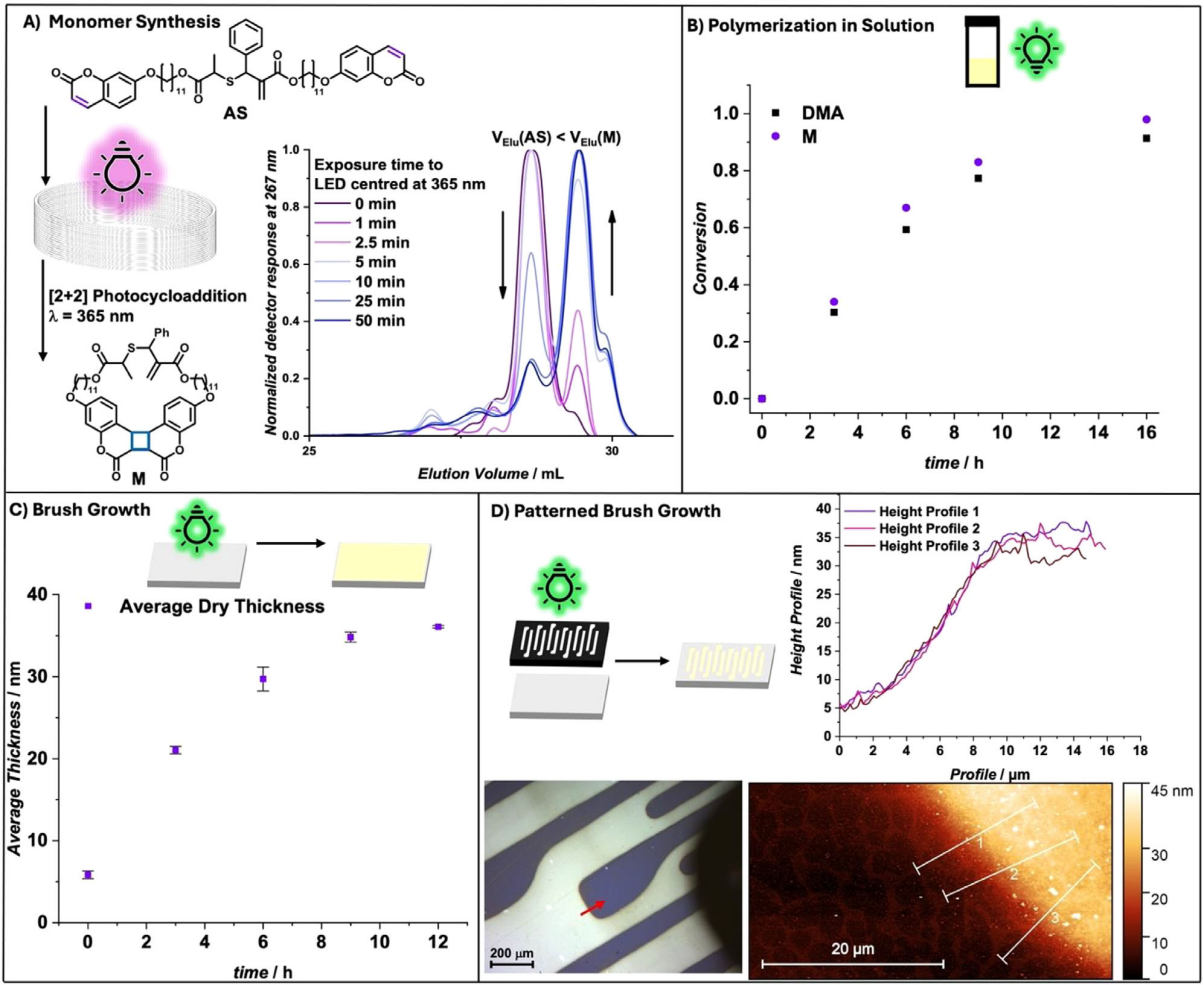
(A) Scaling of the photoresponsive rROP monomer, M, was a prerequisite to enable brush synthesis. Scheme of the scalable intramolecular [2+2] photocycloaddition of allyl sulfide (AS) to the cyclic monomer M using a photoflow‐reactor with LEDs centered at *λ* = 365 nm (left). THF‐SEC of respective products after photoflow‐reaction with different flow rates and thus different exposure times to the LED (right). (B) Conversion of M (purple circles) and DMA (black squares) during PET RAFT polymerization in solution according to ^1^H‐NMR. (C) Average thickness of dry P(DMA‐co‐M) brush on Si‐wafer during SI PET RAFT polymerization of M and DMA according to Ellipsometry measurements on three random locations per wafer (purple squares). Error bars represent a 95% confidence interval for each data point. (D) Optical microscopy and AFM images of a patterned P(DMA‐co‐M) polymer brush obtained using a photomask during SI PET RAFT. Height profiles of 3 arbitrary lines from the AFM image.

To generate scalable access to the photodegradable CAS monomer, **M** [53], a flow synthesis‐based access route was developed first (Figure 1A). A solution of the telechelic coumarin functionalized allyl sulfide precursor, **AS**, in acetonitrile (2 mg/mL) was passed through a photoflow‐reactor at different flowrates yielding exposure times of 1–50 min of an LED light centered at *λ* = 365 nm. Upon intramolecular [2+2] photocycloaddition, the cyclization of the monomer yields a smaller solvated volume, resulting in higher elution volumes in size‐exclusion chromatography (SEC).

The successful [2+2] photocycloaddition was confirmed via THF‐SEC with the UV detector set to the isosbestic point of the linear precursor **AS** and **M** at *λ* = 267 nm, indicating reaction completion after 10 min of light exposure (flow rate $\dot{V}=0.5mL$ min^−1^ in a 5 mL tubing). By performing the intramolecular [2+2] photocycloaddition in flow, the production rate was increased 12‐fold from 0.083 mg min^−1^ in batch [53] to 1 mg min^−1^.

PET‐RAFT copolymerization was performed in solution to investigate the polymerization kinetics and the resulting polymer structures. The conversion of both monomers was monitored via ^1^H‐NMR spectroscopy, revealing similar conversions for **M** and *N*,*N*‐Dimethylacrylamide (DMA) (Figure 1B) reaching >90% after 16 h of irradiation with green light. Thus, the resulting *P(DMA‐*co‐***M***) copolymers are expected to display an almost statistical incorporation of **M**. The polymerization process was then transferred onto surfaces using SI‐PET‐RAFT promoted brush growth from a silicon surface functionalized with ethyl (1‐oxo‐1‐((3‐(triethoxysilyl)propyl)amino)propan‐2‐yl)carbonotrithioate (Figures S13 and S14). Using a ratio of 1:49 of **M** to DMA, brush growth was monitored in the dry state via ellipsometry on 3 random spots per sample (data fitted with a Cauchy model [55], Figure 1C). The dry thickness of the P(DMA‐*co*‐**M**) brush increased to a polymer film thickness of 36.1 ± 0.14 nm with irradiation times up to 12 h. Assuming comparable polymerization kinetics between solution and surface‐initiated polymerization (Figure 1B), the photodegradable monomer **M** is likely statistically embedded across the height of the polymer brush architecture. XPS further supports a largely statistical incorporation of **M**, yielding C 75.4 at %, N 10.8 at %, O 13.2 at %, and S 0.6 at % (Figure S34). This data compares well with the elemental composition expected for a DMA brush containing 2 mol% of **M** (C 73.2 at %, N 12.0 at %, O 14.5 at %, and S 0.25 at %).

Patterned polymer brushes were grown on Si wafers by irradiating the polymerization mixture on a CTA functionalized surface under a photomask. The resulting patterned surfaces were imaged in the dry state via optical microscopy and atomic force microscopy (AFM) (Figure 1D). In agreement with the ellipsometry measurements, a dry brush height of close to 35 nm was measured under otherwise identical polymerization conditions, with interface widths of a few micrometers showing control over the polymerization process on the surface. Further experiments to tune the brush height were performed and showed a dependency on the molarity of the polymerization (Section S5.4.2).

**FIGURE 2:**
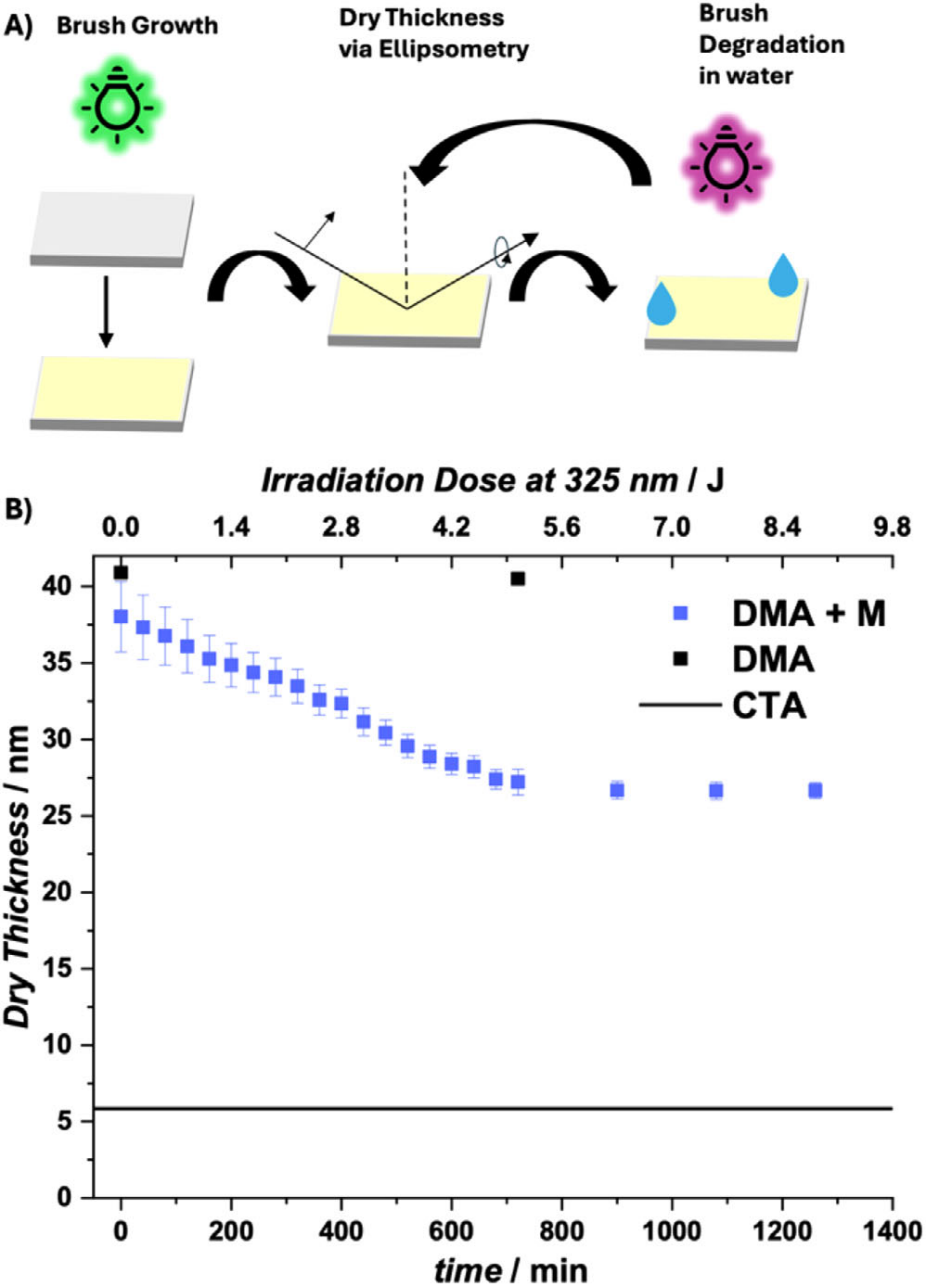
(A) Scheme of polymer brush growth, dry thickness measurement of a P(DMA‐*co*‐**M**) brush via Ellipsometry and brush degradation in water. (B) Dry Thickness change versus irradiation time and irradiation dose; the black line indicates the thickness that was measured after CTA functionalization.

The incorporated coumarin‐based monomer **M** can undergo photocycloreversion, causing the polymer main chain to cleave (Figure 2A). The polymer degradation was confirmed in solution via dimethylacetamide (DMAc) SEC and showed a significant decrease in molar mass from $\bar{M_{n}}$ = 51,000 g mol^−1^ to $\bar{M_{n}}$ = 12,500 g mol^−1^ after irradiating for 30 min with UVB lamps with a peak emission at *λ* = 313 nm in DMAc (Figure S30). After confirming the polymer degradation in solution, the degradation on surfaces was investigated in depth. A non‐patterned polymer brush of a dry thickness of 38 nm was irradiated in water with an LED centered at *λ* = 325 nm at a distance of 3.5 cm, yielding an irradiance of 117 µW on the sample to degrade the polymer brush slowly in a controlled manner. To monitor the decreasing polymer film thickness (Figure 2B, blue squares), the sample was rinsed and dried in a nitrogen flow in 40 min irradiation intervals. The dry polymer film thickness decreased linearly until the original brush thickness was reduced by 34% to 27 nm of brush height after approximately 12 h (5.2 J) of irradiation, after which a plateau was reached (accounting for the thickness of the non‐degradable CTA on the surface). A control experiment with a PDMA homopolymer brush showed no significant decrease in dry film thickness after 12 h of irradiation under identical conditions (Figure 2B, black squares) suggesting that hydrolytic chain detachment of the type reported by de Beer and co‐workers does not play a significant role under the present experimental conditions [56, 57]. Analysis of the water layer used during degradation by SEC‐MS revealed XIC signals at calculated *m*/*z* values consistent with coumarin‐terminated fragments containing 55, 53, 51, and 49 DMA repeat units (Figures S53–S57), which is in agreement with photocycloreversion‐induced polymer cleavage.

The observed plateau is higher than the expected residual thickness based on the statistical spacing of photocleavable units within the copolymer. Under ideal conditions of complete photocleavage, the minimum residual brush thickness is determined by the position of the first successful cleavage event along the surface‐tethered chains rather than the total number of cleavable units, leading to a non‐linear dependence of thickness reduction on cleavable‐site density. Based on the comparable reactivity ratios and a feed ratio of 1:49, the average spacing between cleavable units corresponds to approximately 50 repeat units, yielding a surface‐tethered fragment of similar length. Assuming that the dry brush thickness scales with chain length, this would correspond to a residual thickness of only a few nanometers for an initial brush thickness of 38 nm. The larger experimentally observed plateau, therefore, indicates that the limited reduction is not governed solely by copolymer composition. Instead, the plateau likely arises from a photostationary state in which photocycloaddition competes with photocycloreversion within the confined environment of the degraded polymer matrix, thereby limiting further degradation under the applied irradiation conditions (notably, no significant changes in elemental composition were detected by XPS after irradiation, Figure S34) [58, 59]. We have previously shown that the hydrophobic nature of the monomer M promotes micelle formation, in which the hydrophobic coumarin segments are colocalized in the core [54]. This microenvironment shifts the wavelength‐dependent photochemistry toward bond‐forming photocycloaddition, even under UVB irradiation. Consequently, increasing the content of the degradable monomer **M** to 3.5% did not lead to a significant additional reduction in brush height (Figure S32). In contrast, modifying the monomer hydrophilicity enabled substantially enhanced degradation. A new cyclic allyl sulfide was synthesized, replacing the hydrophobic C11 chains with triethylene glycol to yield a new cyclic allyl sulfide **M′** (Sections S4.4 and S10, Figure S31). Irradiation under otherwise identical conditions resulted in a reduction of the P(DMA‐*co*‐**M′**) brush height by more than half (55%) within the same timeframe (Figure S33). The results confirm that the extent of degradation and hence the maximum achievable structuring depth can be further tuned through rational monomer design.

**FIGURE 3:**
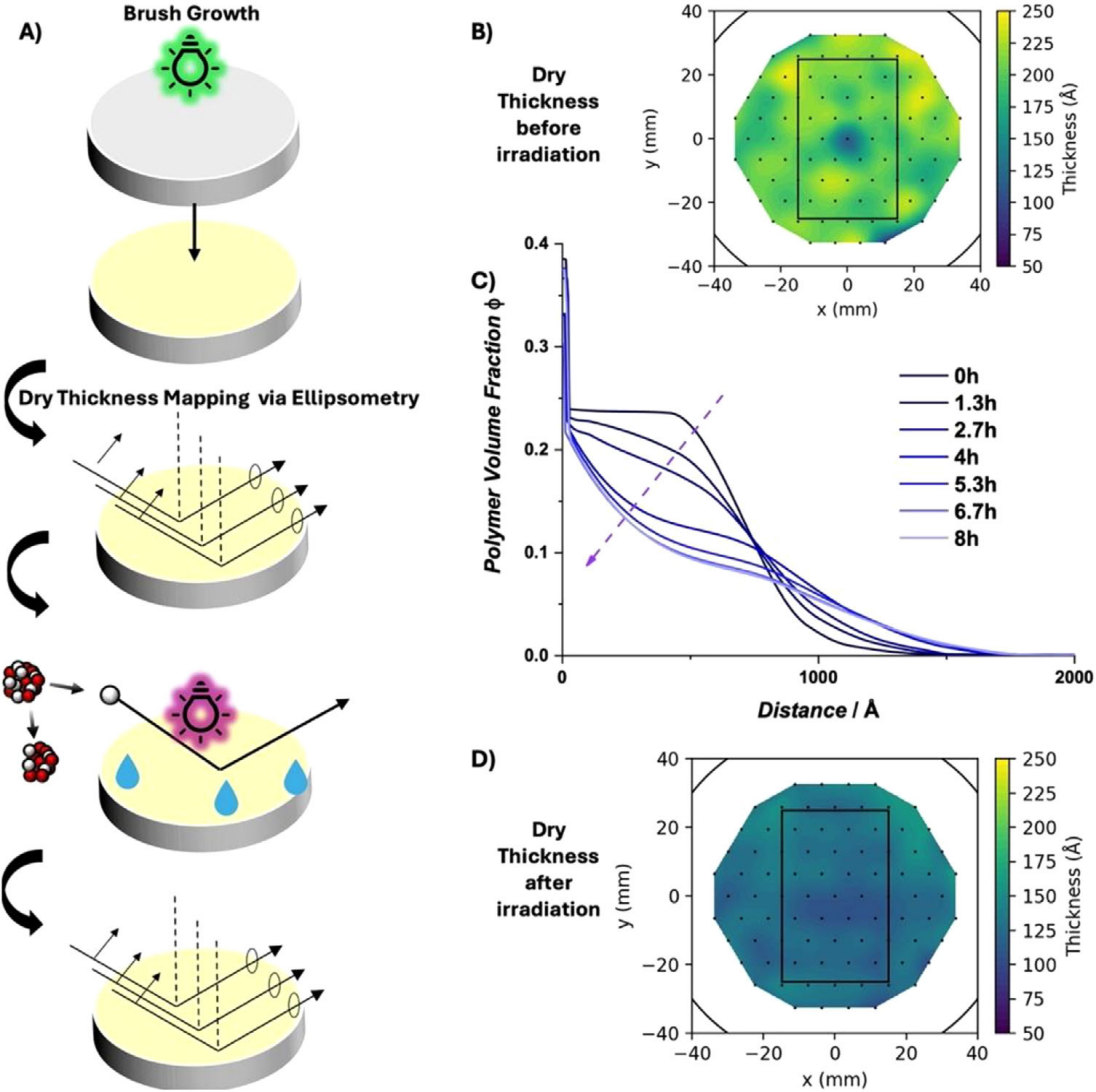
(A) Scheme of brush growth on Si crystals for NR measurements, dry thickness mapping via Ellipsometry, and NR measurements under irradiation with an LED. (B) Polymer film thickness map before irradiation. (C) VFP profiles with progressing irradiation times. (D) Polymer film thickness map after irradiation.

**FIGURE 4:**
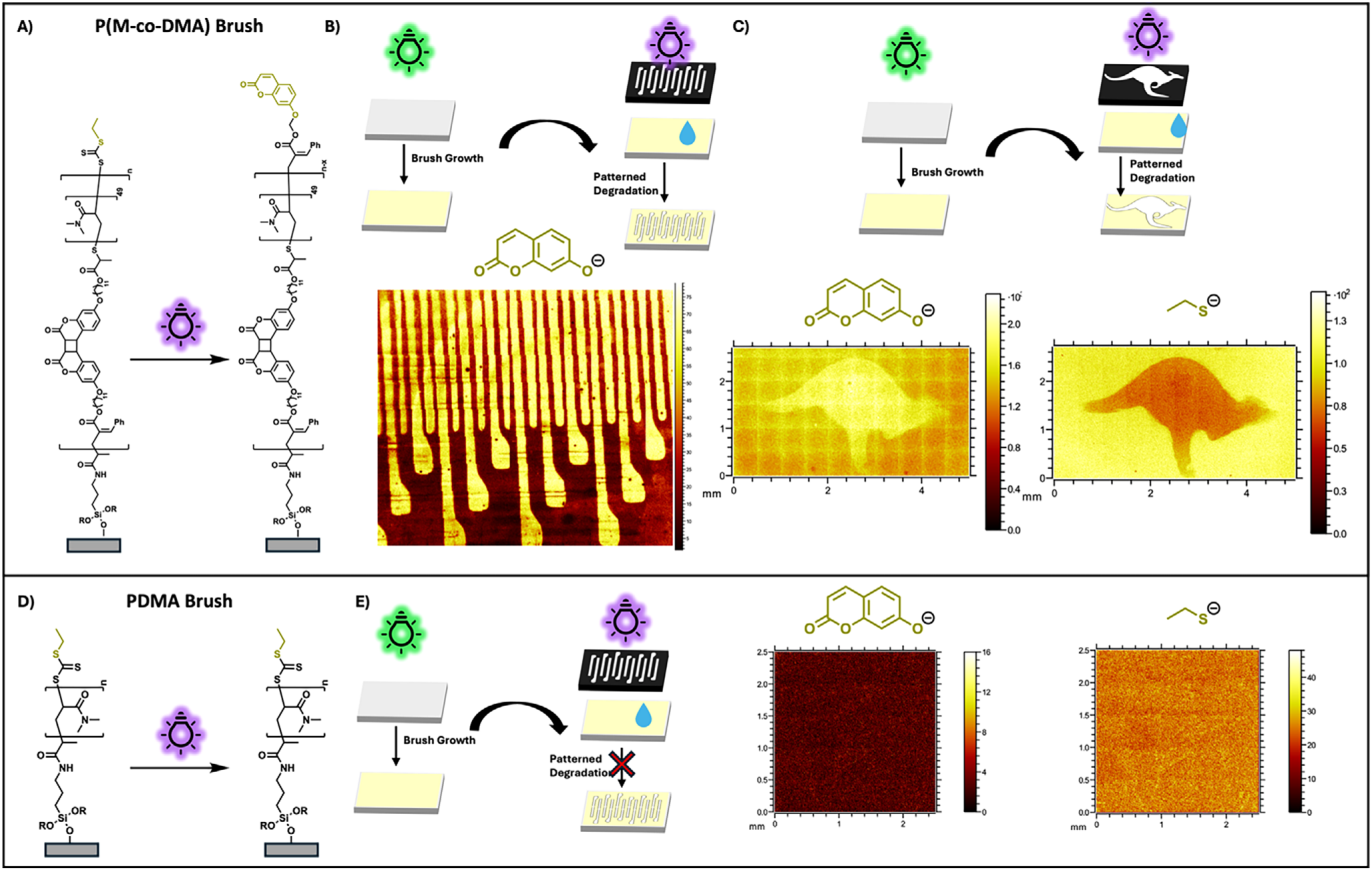
(A) Chemical structure of P(DMA‐*co*‐**M**) copolymer brush before and after irradiation. Upon irradiation, the polymer brush loses the CTA endgroup and is coumarin‐terminated. (B) Scheme of non‐patterned polymer brush growth and patterned polymer brush degradation (top). Resulting ToF‐SIMS image of the C_5_H_9_O_3—_ion attributed to coumarin showing the pattern governed by the photomask (bottom). (C) Scheme of a non‐patterned polymer brush growth and subsequent patterned degradation using a kangaroo photomask (top). ToF‐SIMS images of the C_5_H_9_O_3—_ion attributed to coumarin (middle) and the C_2_H_5_S^−^‐ion attributed to a CTA fragment on the polymer brushes endgroup (bottom), showing the kangaroo pattern governed by the photomask. (D) Chemical structure of DMA homopolymer brush before and after irradiation. (E) Scheme of non‐patterned polymer brush growth and patterned polymer brush degradation (left). Resulting ToF SIMS image of the C_5_H_9_O_3—_ion attributed to coumarin (right, top) and the C_2_H_5_S^−^‐ion attributed to a CTA fragment on the polymer brush's endgroup (right, bottom) showing no pattern.

To investigate polymer degradation in the swollen state, in situ Neutron Reflectometry (NR) measurements in deuterated water were carried out [60, 61, 62]. A non‐patterned P(DMA‐*co*‐**M**) polymer brush was grown on a large silicon wafer suitable for solid‐liquid measurements using neutron reflectometry (Figure 3A) [63]. Due to the diameter of the wafers (*d* = 10 cm), the setup for the polymerization had to be adjusted accordingly. To ensure exposure of the entire crystal to green light, the polymerization setup was built on a rotating carousel (Figure S16 and Scheme S5). As NR measurements in the swollen state are most sensitive close to the surface of the crystal, the brushes were grown at a lower irradiation dose by increasing the distance between the green light source and the sample to obtain thinner polymer films. The brushes were initially analyzed in the dry state via NR and ellipsometry mapping. The average dry polymer film thickness was 20.1 nm according to the ellipsometry map (Figure 3B). The average NR‐derived thickness was identical at 20.1 nm. Subsequently, the P(DMA‐*co*‐**M**) brush on the Si wafer was placed in a D_2_O‐filled liquid cell and irradiated with an LED centered at *λ* = 325 nm at 3.5 cm distance yielding an irradiance of 117 µW. The neutron reflectivity was continuously measured during irradiation. After data acquisition in the liquid cell, the brush was remeasured via NR and ellipsometry mapping in the dry state. Both methods showed a significantly reduced film thickness of 11.1 nm = 111 Å (NR) (12.9 nm = 129 Å via ellipsometry average across map, Figure 4D). The acquired datasets were fitted using the approach outlined by Gresham et al. [60, 61] (Figures S46–S52 and Table S2) and the resulting volume fraction profiles (VFP) overlaid (Figure 3C). Due to the linear decrease in dry film thickness observed previously during irradiation (Figure 2B), linearly scaled values calculated from the dry measurements at the start were used during the NR modelling process. With rradiation time, a change in the VFPs shows a transition from a parabolic profile to an exponentially decaying profile. This demonstrates that irradiation induces a pronounced restructuring of the brush architecture in the swollen state, although the present data do not allow a unique assignment of the underlying molecular mechanism.

According to the proposed brush degradation mechanism, the cycloreversion of backbone‐embedded **M** yields coumarin‐terminated polymer fragments upon main chain scission [53]. To prove that the degradation of these polymer brushes is a consequence of the cycloreversion of **M**, a non‐patterned brush was irradiated with UVB light under a photomask in water for 30 min and subsequently analyzed via time‐of‐flight secondary‐ion mass spectrometry (ToF‐SIMS) to map the distribution of fragment ions attributed to coumarin (C_9_H_5_O_3_^−^, Figure 4B,C). The resulting ion map reflects the pattern of the photomask used for the irradiation, indicating a higher abundance of coumarin moieties at the outer surface of the brush in the irradiated areas. Additionally, the intensity of ions attributed to the terminal CTA fragment (C_2_H_5_S^−^, Figure 4C) decreased in the irradiated areas, indicating the cleavage of the polymer chains. In contrast, a pure PDMA brush exposed to identical irradiation conditions did not show any changes in intensity inside or outside the irradiated areas, confirming the CTA's photostability (Figure 4E). Thus, changes in CTA abundance likely result from the cycloreversion of embedded **M**. Further, pure PDMA brushes did not show significant signal intensity for the C_9_H_5_O_3_^−^ ion assigned to coumarin (Figure 4E).

To investigate whether physical properties of the P(DMA‐*co*‐**M**) polymer brush change after photocycloreversion‐based degradation and the release of coumarin end groups, non‐patterned brushes were compared in either the pristine state or after exposure to UVB in water. Contact angle measurements were performed, showing a contact angle of 71.0° for a pristine P(DMA‐*co*‐**M**) brush and after 30 min of UVB irradiation, indicating no significant change in hydrophilicity throughout the polymer brush degradation (Figures S38–S40). Adhesion measurements on the same samples were performed via AFM and showed no significant difference in adhesion for the pristine brush (221 pN ± 16 pN) and the degraded brush (218 pN ± 16 pN) (Figure S40).

**FIGURE 5:**
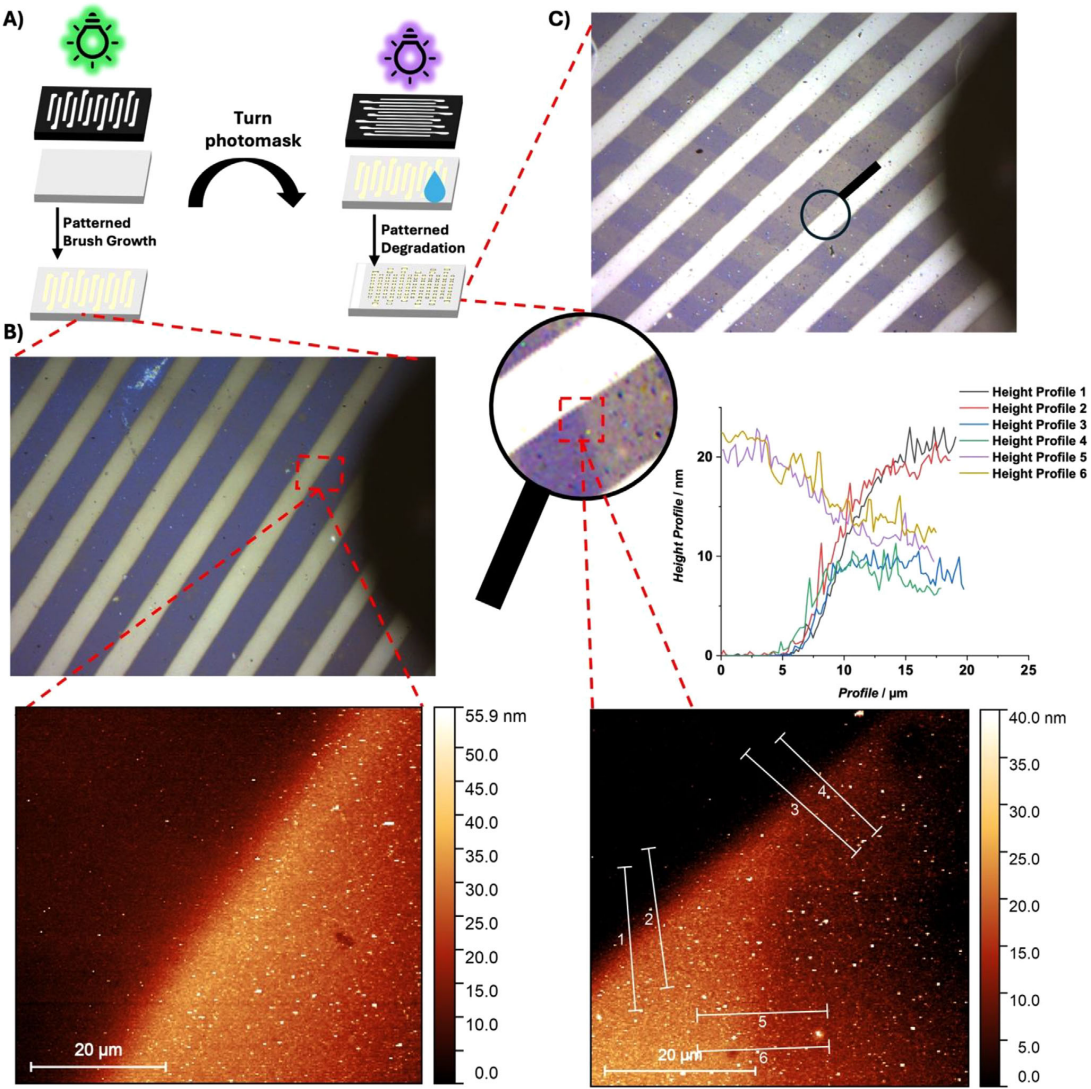
(A) Scheme of patterned growth of a P(DMA‐co‐**M**) brush and patterned polymer brush degradation with a rotated photomask. (B) Microscope image of the pristine, patterned polymer brush (top) with AFM image of the highlighted position (bottom). (C) Microscope of the cross‐patterned degraded polymer brush (top) and zoom of the cross‐patterned degraded polymer brush (middle, left) and the corresponding AFM image (bottom). Height profiles along the lines shown in the AFM image displaying the silicon—non‐degraded brush interface (1‐2), silicon—degraded brush interface (3‐4), and non‐degraded brush—degraded brush interface (5‐6).

To investigate the potential of subtractive 3D structuring of polymer brushes, patterned polymer brushes were grown and imaged via optical microscopy and AFM (Figure 5A). The photomask used for the brush growth was then rotated, and the sample was irradiated in water with UVB light for 30 min. As a result, the brush height in the UVB light irradiated areas was decreased in its dry thickness from close to 26 nm to around 10 nm (Figure 5B)—giving rise to 3D structured polymer brushes.

## 3 Conclusion

In summary, we report a subtractive structuring approach for the spatiotemporally controlled modification of polymer brushes with non‐specialized equipment using only water and light. Photoflow cyclisation of coumarin‐based allylic sulfides yielded the cyclic monomer **M**. Upon radical ring opening copolymerization with DMA using PET‐RAFT, structured and non‐structured P(DMA‐*co*‐**M**) brushes were obtained. Ellipsometry measurements showed the linear increase of dry polymer film thickness during the polymerization process, which aligned with the conversion of both monomers monitored via ^1^H‐NMR spectroscopy. Dry P(DMA‐*co*‐**M**) polymer brush thicknesses of 20–40 nm measured via AFM were obtained depending on the concentration of monomer solution and irradiation setup. The polymer brush photolysis was investigated in depth via ellipsometry, showing a linear decrease in dry film thickness and neutron reflectometry, enabling a structured reduction in brush height of over 50%, also depending on the hydrophilicity of the photolysable monomer. The increased signal intensity of coumarin ions in areas irradiated with UV light, observed in ToF‐SIMS data, corroborates the proposed cycloreversion mechanism. Importantly, neither hydrophilicity nor adhesion force was affected upon brush photolysis. The reported strategy decouples the surface polymerization process from the subtractive structuring process, opening the way to on‐demand structuring of surfaces whilst retaining their physical properties.

## Author Contributions

**Henrik Kalmer**: conceptualization, investigation, writing – original draft, visualization, writing – review & editing, methodology, software, data curation, formal analysis, validation. **Federica Sbordone**: investigation, writing – review and editing, formal analysis, methodology, conceptualization, data curation. **Phuong T. Do**: investigation, writing – review and editing, data curation, methodology. **Kai Mundsinger**: investigation, writing – review and editing, data curation, methodology, formal analysis. **Hazal Kayas**: visualization, formal analysis, data curation, writing – review & editing. **Robert T. Jones**: investigation, writing – review & editing, data curation, formal analysis. **Jayanti Mendhi**: investigation, writing – review and editing, data curation, formal analysis. **Tim R. Dargaville**: investigation, writing – review and editing, formal analysis. **Damien G. Harkin**: investigation, formal analysis, data curation, methodology, writing – review and editing. **Lukas Michalek**: writing – review & editing, investigation, conceptualization, methodology, formal analysis, data curation. **Andrew Nelson**: data curation, supervision, resources, formal analysis, software, writing – review and editing, validation, methodology, investigation, funding acquisition. **Hendrik Frisch**: conceptualization, investigation, funding acquisition, writing – original draft, writing – review and editing, project administration, resources, supervision, formal analysis, visualization.

## Conflicts of Interest

The authors declare no conflicts of interest.

## Supporting information




**Supporting File**: The authors have cited additional references within the Supporting Information [27, 48, 58, 59, 60, 61, 64].

## Data Availability

The data that support the findings of this study are available from the corresponding author upon reasonable request.
