# Supplementary material for: Photolyzable Polymer Brushes: Subtractive 3D Structuring of Surfaces Using Water and Light
Source: Angew Chem Int Ed Engl. 2026 Apr 13;65(22):e2790800. doi: 10.1002/anie.2790800 (PMC13206511; doi:10.1002/anie.2790800)
Supplement: Supplementary file 1 — Supporting File: The authors have cited additional references within the Supporting Information [27, 48, 58, 59, 60, 61, 64]. [file ANIE-65-e2790800-s001.pdf]

Photolyzable Polymer Brushes: Subtractive 3D Structuring of Surfaces using Water and Light

Henrik Kalmer, Federica Sbordone, Phuong T. Do, Kai Mundsinger, Hazal Kayas, Robert T. Jones, Jayanti Mendhi, Tim R. Dargaville, Damien G. Harkin, Lukas Michalek, Andrew Nelson and Hendrik Frisch

1 Contents

2 Materials 5
3 Experimental Details 6
3.1 NMR 6
3.2 LCMS 6
3.3 DMAc SEC 6
3.4 THF SEC 6
3.5 XPS 7
3.6 ToF-SIMS 7
3.7 AFM 7
3.8 Optical Tensiometer 7
3.9 Ellipsometry Maps 7
3.10 Single Point Ellipsometry Measurements 7
3.11 Neutron Reflectometer Platypus 8
3.12 Puriflash 8
3.13 Photoflowreactor 8
3.14 SEC-ESI-MS 9
3.15 Photoreactor 10
3.16 Scanning electron microscopy 10
4 Monomer synthesis: 11
4.1 AS 11
4.2 AS' 13
C1 13
AS' 14
4.3 M 16
4.4 M' 17
5 Polymerisations 18
5.1 ZnHTPP 18
5.2 CTA and surface functionalization 20
5.3 Polymerization setup 24
5.4 Patterned polymer brushes 27
5.4.1 Standard conditions 27
5.4.2 0.5 M monomer solution 29
5.5 AFM of scratched non-structured polymer brushes 32
6 Photodegradation 34
6.1 Photodegradation setup 34

6.2 Polymer photodegradation ... 36
6.2.1 Polymer photodegradation in solution ... 36
6.2.2 Polymer Brush Photodegradation ... 38
6.2.3 XPS of Polymer Brushes ... 39
6.3 Polymer Brush Properties ... 42
6.3.1 Contact angle measurements ... 42
6.3.2 Adhesion measurements ... 43
7 Characterization of large wafers (d = 10 cm) ... 44
8 Fitting Liquid Cell Neutron Reflectometry Data ... 46
9 Extracted Ion Chromatograms of Degraded Polymer Brushes ... 50
10 Time-Dependent Conversions of DMA and M’ ... 53
11 Literature ... 55

List of abbreviations

AFM:	Atomic force microscopy
CTA:	Chain transfer agent
DBU:	1,8-Diazabicyclo[5.4.0.]undec-7-ene
DCM:	Dichloromethane
DMA:	*N*,*N*-Dimethylacrylamide
DMAc:	*N*,*N*-Dimethylacetamide
DMSO:	Dimethylsulphoxide
LC-ESI-HRMS:	Liquid Chromatography-Electrospray Ionisation-High Resolution Mass Spectrometry
NMR:	Nuclear magnetic resonance
NR:	Neutron reflectometry
PET-RAFT	Photoinduced energy/electron transfer reversible addition fragmentation chain transfer
SEC:	Size-exclusion chromatography
TFA:	Trifluoroacetic acid
ToF-SIMS:	Time-of-flight secondary ion mass spectrometry
XPS:	X-ray photoelectron spectroscopy
ZnHTPP:	Zinc tetra-4-hydroxyphenylporphyrin

2 Materials

Unless stated otherwise, all materials were used as received without further purification.

Benzaldehyde (Sigma- Aldrich, 99.0% min), tert-butyl acrylate (Sigma-Aldrich, 98.0% min), 1,8-Diazabicyclo [5.4.0] undec-7-ene (Sigma- Aldrich, 98.0% min), hydrochloric acid (32% in water, Thermo Fisher Scientific), sodium sulfate (anhydrous, granular, Thermo Fisher Scientific, 99.0% min), 4-methoxyphenol (Sigma-Aldrich, 99.0% min), acetic anhydride (Chem-Supply , 99.0% min), *N*, *N*-dimethylaminoprydine (Sigma-Aldrich, 99.0%), dichloromethane (Chem-Supply, 99.8%), ethyl acetate (Thermo Fisher Scientific, 99.5%), cyclohexane (Sigma-Aldrich, 99.5%), 1,4-Diazabicyclo [2.2.2] octane (Sigma-Aldrich, 99.0% min), THF (Thermo Fisher Scientific, 99.7%), 2-mercaptopropionic acid (Sigma-Aldrich, 95.0% min), trifluoracetic acid (Sigma-Aldrich, 99.0% min), acetonitrile (Fisher Scientific, 99.9% min), 7-hydroxycoumarin (Comni-Blocks, 98.0%, min), 11-bromo-1-undecanol (Combi-Blocks, 98.0% min), anhydrous potassium carbonate (Chem-Supply, 99.0% min), potassium iodine (Chem-Supply, 99.0%), acetone (Themo Fisher Scientific, 99.0% min), oxalyl chloride (Sigma-Aldrich, 98.0% min), dimethylformamide (Thermo Fisher Scientific, 99.8% min), pyridine (Sigma-Aldrich, 99.0% min), *N*,*N*-Dimethylacrylamide (Sigma Aldrich) (DMA) (purified through an aluminium oxide column), toluene (Thermo Fischer Scientific, 99.8%), .methanol (Thermo Fischer Scientific, 98%), dimethyl sulfoxide (Thermo Fischer Scientific, 99 %), 5,10,15,20-Tetrakis(4-hydroxyphenyl)-21h,23h-porphine (Combi Blocks, 95%), zinc acetate (99.99 % trace metal basis), (3-Aminopropyl)triethoxysilane (Merck Life Science, 98%), 1-Ethyl-3-(3-dimethylaminopropyl)carbodiimide hydrochloride (Thermo Fischer Scientific), ethanethiol (Merck Life Science), carbon disulfide (Merck Life Science), potassium hydroxide (Chem-Supply, AR), 2-Bromopropionic acid (Merck Life Science, 99%), osmium tetraoxide (ProSciTech), sodium cacodylate (ProSciTech), hexamethyldisilazane (Sigma Aldrich).

Silicon wafers:

Small silicon wafers n-type Si:P; [111] ±0.5°; SEMI Prime by *El-CAT Inc.*

Large silicon wafers (10 cm diameter and 1 cm thickness) were provided by the Australian Nuclear Science and Technology Organisation (ANSTO).

3 Experimental Details

3.1 NMR

^{1}H-NMR as well as, COSY-spectra were recorded on a *Bruker* System 600 Ascend LH, equipped with a BBO-Probe (5 mm) with z-gradient (^{1}H: 600.13 MHz). Resonances are reported in parts per million (ppm) relative to tetramethylsilane (TMS). The δ-scale was calibrated to the respective solvent signal of $CHCl_3$ or Acetone for ^{1}H NMR spectra. The annotation of the signals is based on HSQC-, COSY- and DEPT-experiments.

3.2 LCMS

LC-MS measurements were performed on an UltiMate 3000 UHPLC System (Dionex, Sunnyvale, CA, USA) consisting of a pump (LPG 3400SZ), autosampler (WPS 3000TSL) and a temperature controlled column compartment (TCC 3000). Separation was performed on a C18 HPLC column (Phenomenex Luna 5µm, 100 Å, 250 × 2.0 mm) operating at 40 °C. Water (containing 5 mmol L-1 ammonium acetate) and acetonitrile were used as eluents. A gradient of acetonitrile: H2O, 5:95 to 100:0 (v/v) in 7 min at a flow rate of 0.40 mL·min-1 was applied. The flow was split in a 9:1 ratio, where 90% of the eluent was directed through a DAD UV-detector (VWD 3400, Dionex) and 10% was infused into the electrospray source. Spectra were recorded on an LTQ Orbitrap Elite mass spectrometer (Thermo Fisher Scientific, San Jose, CA, USA) equipped with a HESI II probe. The instrument was calibrated in the m/z range 74-1822 using premixed calibration solutions (Thermo Scientific). A constant spray voltage of 3.5 kV, a dimensionless sheath gas, and a dimensionless auxiliary gas flow rate of 5 and 2 were applied, respectively. The capillary temperature was set to 300 °C, the S-lens RF level was set to 68, and the aux gas heater temperature was set to 100 °C.

3.3 DMAc SEC

The SEC measurements were conducted on a *PSS* SECurity^2 system consisting of a *PSS* SECurity Degasser, *PSS* SECurity TCC6000 Column Oven (60 °C), *PSS* GRAM Column Set (8x150 mm 10 µm Precolumn, 8x300 mm 10 µm Analytical Columns, 1000 Å, 1000 Å and 30 Å) and an *Agilent* 1260 Infinity Isocratic Pump, *Agilent* 1260 Infinity Standard Autosampler, *Agilent* 1260 Infinity Diode Array and Multiple Wavelength Detector (A: 254 nm, B: 360 nm), *Agilent* 1260 Infinity Refractive Index Detector (35 °C). HPLC grade DMAc, 0.01 M LiBr, is used as eluent at a flow rate of 1 mL·min^{-1}. Narrow disperse linear poly(styrene) (M_n: 266 g·mol^{-1} to 2.52x10^6 g·mol^{-1}) and poly(methyl methacrylate) (M_n: 202 g·mol^{-1} to 2.2x10^6 g·mol^{-1}) standards (*PSS* ReadyCal) were used as calibrants. All samples were passed over 0.22 µm PTFE membrane filters. Molecular weight and dispersity analysis was performed in *PSS* WinGPC UniChrom software (version 8.2).

3.4 THF SEC

The SEC measurements were conducted on a *PSS* SECurity^2 system consisting of a *PSS* SECurity Degasser, *PSS* SECurity TCC6000 Column Oven (35 °C), *PSS* SDV Column Set (8x150 mm 5 µm Precolumn, 8x300 mm 5 µm Analytical Columns, 100000 Å, 1000 Å and 100 Å) and an *Agilent* 1260 Infinity Isocratic Pump, *Agilent* 1260 Infinity Standard Autosampler, *Agilent* 1260 Infinity Diode Array and Multiple Wavelength Detector (A: 254 nm, B: 360 nm), *Agilent* 1260 Infinity Refractive Index Detector (35 °C). HPLC grade THF, stabilized with BHT, is used as eluent at a flow rate of 1 mL·min^{-1}. Narrow disperse linear poly(styrene) (M_n: 266 g·mol^{-1} to 2.52x10^6 g·mol^{-1}) and poly(methyl methacrylate) (M_n: 202 g·mol^{-1} to 2.2x10^6 g·mol^{-1}) standards (*PSS* ReadyCal) were used as calibrants. All

samples were passed over 0.22 µm PTFE membrane filters. Molecular weight and dispersity analysis was performed in *PSS* WinGPC UniChrom software (version 8.2).

3.5 XPS

Spectra were recorded on a *Kratos* Axis Supra photoelectron spectrometer. During analysis, the charge compensation system was employed to prevent any localised charge build-up. For each sample, wide spectra and high-resolution spectra of individual peaks (e.g N 1s) were recorded. All spectra were calibrated by setting the C 1s peak to 285.00 eV. Evaluation, peak deconvolution, and fitting was carried out in *Casa Software Ltd* CasaXPS 2.3.

3.6 ToF-SIMS

ToF-SIMS data were acquired using an IONTOF M6 instrument (IONTOF GmbH, Münster, Germany) equipped with a reflectron time-of-flight analyser and 30 kV Bi/Mn primary-ion source. Bi_3^+ cluster ions were selected from the pulsed primary ion beam for the analysis and 'bunched' to attain optimal mass resolution ($M/\Delta M > 8500$ for $C_9H_5O_3^-$, at m/z 161). The measured target current was approximately 0.5 pA, and the primary-ion dose limited to 1×10^{-11} ions cm^{-2}. A cycle time of 100 µs provided an accessible mass range up to m/z 500.

Spectra were acquired in both positive and negative polarity; however, only negative data are reported, as all relevant analytical secondary ions were anions. The mass scale was calibrated using peaks attributed to hydrocarbon ions (C^-, C_2^-, C_3^-, C_4^-, C_5^-, C_6^-, C_7H^-). During data acquisition, the pressure in the analysis chamber was maintained at, or below, 5×10^{-9} mbar.

3.7 AFM

AFM measurements were performed on Bruker Dimension Icon Atomic Force Microscope (AFM) in ScanAsyst mode equipped with the ScanAsyst-Air AFM cantilever (0.4 N/m spring constant, 70kHz resonant frequency, 2nm tip radius).

3.8 Optical Tensiometer

Contact angle measurements were performed on a Biolin Scientific Theta Flex Optical Tensiometer equipped with a CMOS 2/3'' USB 3.0 digital camera with zoom using the OneAttension software and a drop volume of 5 µL.

3.9 Ellipsometry Maps

Ellipsometry maps were acquired on an Accurion EP4 variable angle spectroscopic ellipsometer equipped with a 658 nm laser. Several datasets were acquired across the sample to create the respective topographical map using refellips.[1]

3.10 Single Point Ellipsometry Measurements

All single point ellipsometry measurements were performed on an J. A. Woollam M-2000UI with a wavelength range of 4245 – 1690 nm. These measurements were done with incidence angles from 45 to 65° (in 10° steps) and fitted by a Cauchy model using the CompleteEASE software.

3.11 Neutron Reflectometer Platypus

Neutron reflectometry (NR) measurements were carried out on the Platypus time-of-flight reflectometer at the OPAL 20MW reactor (Australian Nuclear Science and Technology Organisation, Sydney).

The brush structures were measured by NR in air (collapsed) and D_2O (swollen) for the in-situ experiments conducted in a solid-liquid flow cell using a (roughened) quartz backing plate, for later irradiation with an UV-LED. NR measurements of the dry films were made at angles of incidence of 0.65 and 3.00 in air giving a Q range of 0.0077 – 0.23 Å^{-1}. Subsequently, reflectivity measurements of the solvated brush were made at angles of incidence of 0.65 and 3.50 (reflecting downwards) in D_2O giving a Q range of 0.077 – 0.27 Å^{-1}. The cell was irradiated for up to 18 h using and UV-LED ($\lambda = 325\ nm, 4.5\ V, I = 0.15\ A$), which was placed 3.5 cm above the cell.

3.12 Puriflash

Flash chromatography was performed on a *Interchim* XS420+ flash chromatography system consisting of a SP-in-line filter 20-µm, an UV-VIS detector (200-800 nm) and a *SofTA* Model 400 ELSD (55 °C drift tube temperature, 25 °C spray chamber temperature, filter 5, EDR gain mode) connected via a flow splitter (*Interchim* Split ELSD F04590). The separations were performed using an Interchim dry load column (liquid injection) and a Interchim Puriflash Silica HP 30 µm column for normal phase separations and a Interchim Puriflash C18-HP 30 µm column for reverse phase separations.

3.13 Photoflowreactor

Photoreactions under flow conditions were performed using a *Vapourtec* E-series platform (peristaltic pumps) in combination the UV-150 module and the VSD006 cooling module. The module consists of a temperature-controlled irradiation chamber, a transparent fluorinated ethylene polymer (FEP) reactor coil (1.3 mm inner diameter, 0.15 mm wall thickness, 5 mL PN: 50-1287) and a LED assembly (360 to 390 nm, peak 365 nm, total power output of 18 W, PN: 50-1438). The temperature is controlled employing pre-cooled nitrogen (heat exchange in the cooling module).

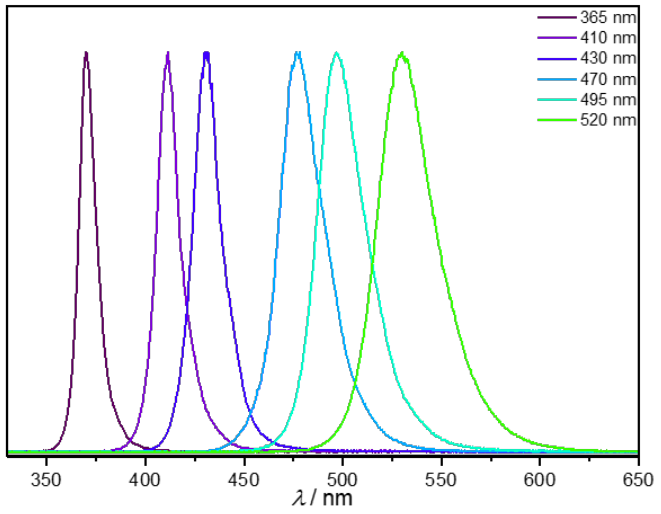

Figure S1: Emission spectra of the Vapourtec LEDs.

3.14 SEC-ESI-MS

Size exclusion chromatography coupled with electrospray ionisation mass spectrometry experiments were measured using a Q Exactive Plus Biopharma mass spectrometer (Thermo Fisher Scientific, San Jose, CA, USA) equipped with a HESI-II ionisation source. The mass spectrometer was calibrated up to *m/z* 2000 using premixed calibration solution (Pierce; Thermo Scientific) and for high mass mode (*m/z* 600-8000) using ammonium hexafluorophosphate solution. A constant spray voltage of +3.5 kV, a dimensionless sheath gas and a dimensionless auxiliary gas flow rate of 10 and 0 were applied, respectively. The capillary temperature was set to 320 °C, the S-lens RF level was set to 150 and the auxiliary gas heater temperature was set to 125 °C. The Q Exactive was coupled to an UltiMate 3000 UHPLC System (Dionex, Sunnyvale, CA, USA) consisting of a pump (LPG 3400SD), autosampler (WPS 3000TSL), and a temperature-controlled column department (TCC 3000). Separation was performed PSS SDV Column Set (8x150 mm 5 µm Precolumn, 8x300 mm 5 µm Analytical Columns, 100000 Å, 1000 Å and 100 Å) operating at 30 °C. THF at a flow rate of 0.30 mL min^{-1} was used as eluent. The mass spectrometer was coupled to the column in parallel with an UV-detector (VWD 3400, Dionex) and a Refractive Index detector (RefractoMax520, ERC, Japan). A split flow of 0.27 mL min^{-1} of the eluent were directed through the UV- and RI-detector and the remaining 30 µL min^{-1} directed into the electrospray source following post-column addition of 50 µM sodium iodide in methanol at 20 µL min^{-1} by a micro-flow HPLC syringe pump (Teledyne ISCO, Model 100DM). A 100 µL aliquot of polymer solution at 2 mg mL^{-1} concentration was injected into the SEC system for analysis.

3.15 Photoreactor

For UVB degradation:

The samples were irradiated in a *Luzchem* LZC-4V photoreactor using LZC-UVB lamps, centered at ~300 nm, with a peak of 313 nm. 2 lamps were installed for side irradiation. One lamp was installed for top irradiation. The internal chamber was ventilated to maintain ambient temperature during the entire experiment.

For PET-RAFT:

The samples were irradiated in a *Luzchem* LZC-4V photoreactor using LZC-LGR emitting at λ = 510-530 nm. 6 lamps were installed for top irradiation. The internal chamber was ventilated to maintain ambient temperature during the entire experiment. Homogeneous irradiation from all directions was ensured by rotating the sample inside the reactor using the LZC-B carousel.

The irradiance was determined with a powermeter *PM400 Optical Power Meter* by *Thorlabs*. The lamps were switched on and given 20 min to heat up and stabilize. Subsequently, the irradiance was measured for >10 min in intervals of 1 s.

**Lamp**	**Mean Irradiance [mW/cm^2]**
UVB	76.79 ± 0.63
Blue	118.19 ± 1.45
Green	79.96± 1.53

Table S1: Irradiance of photoreactor lamps.

3.16 Scanning electron microscopy

Scanning electron microscopy (SEM) images were acquired in a *Tescan MIRA 3 SEM* using 5 kV and 8 beam intensity. Samples were fixed using 2.5 % glutaraldehyde in PBS, followed by buffer rinsing with sodium cacodylate buffer and fixation using 1 % aqueous osmium tetroxide. Dehydration was carried out using increasing concentrations of ethanol followed by overnight critical drying using hexamethyldisilazane (HMDS). All procedures were conducted using standard protocol in the *Pelco Biowave* microwave processing system. Samples were mounted on SEM stubs and sputter coated with a 5 nm platinum conductive coating (Leica Microsystems) for observation.

4 Monomer synthesis:

4.1 AS

The allyl sulfide **AS** was synthesised as previously described in literature.[2]

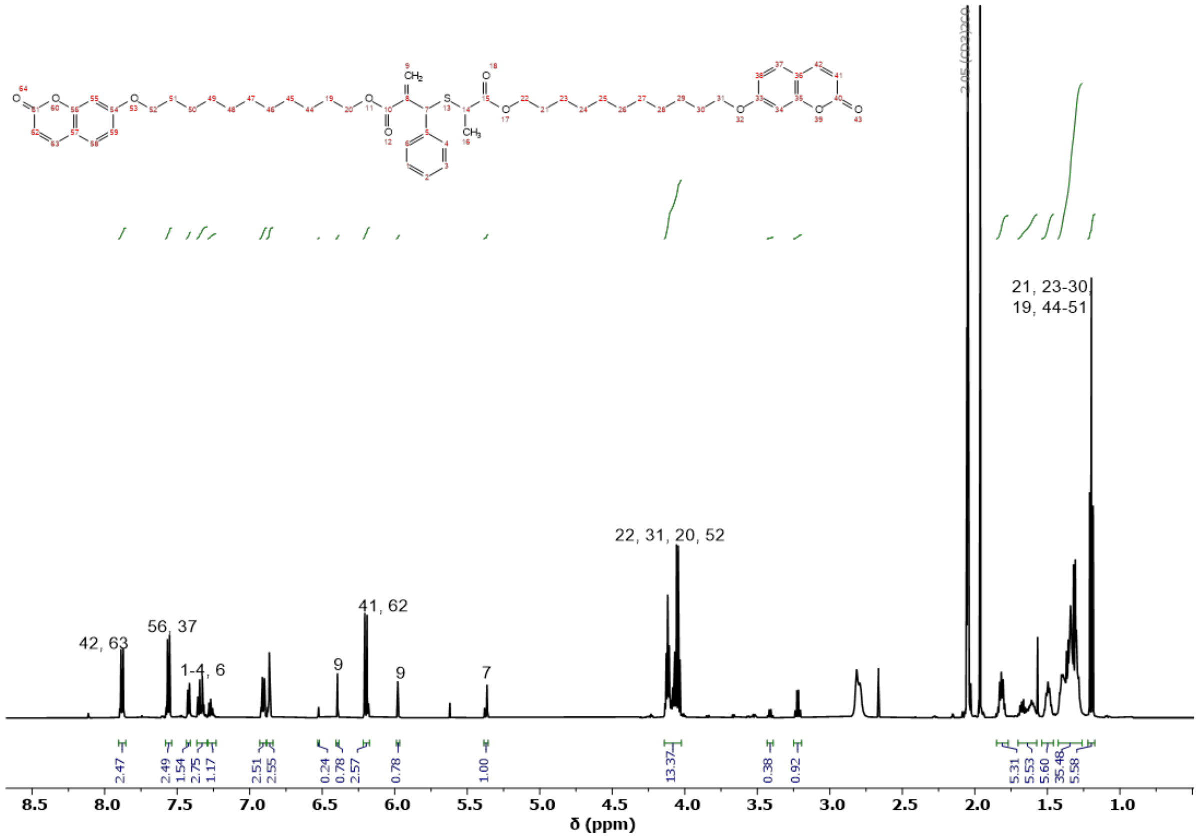

*Figure S2: ^{1}H-NMR of **AS** in acetone-d$_6$.*

^{1}H NMR (600 MHz, acetone-d$_6$, *main isomer*) δ 7.97 – 7.79 (m, 2H), 7.56 (d, J = 8.6 Hz, 2H), 7.50 – 7.37 (m, 2H), 7.37 – 7.23 (m, 2H), 6.40 (d, J = 0.8 Hz, 1H), 6.20 (d, J = 9.5 Hz, 2H), 5.98 (dd, J = 1.2, 0.8 Hz, 1H), 5.42 – 5.32 (m, 1H), 4.21 – 3.98 (m, 8H), 3.22 (d, J = 7.2 Hz, 1H), 1.86 – 1.75 (m, 4H), 1.73 – 1.56 (m, 4H), 1.53 – 1.43 (m, 4H), 1.43 – 1.26 (m, 36H), 1.20 (t, J = 7.1 Hz, 6H).

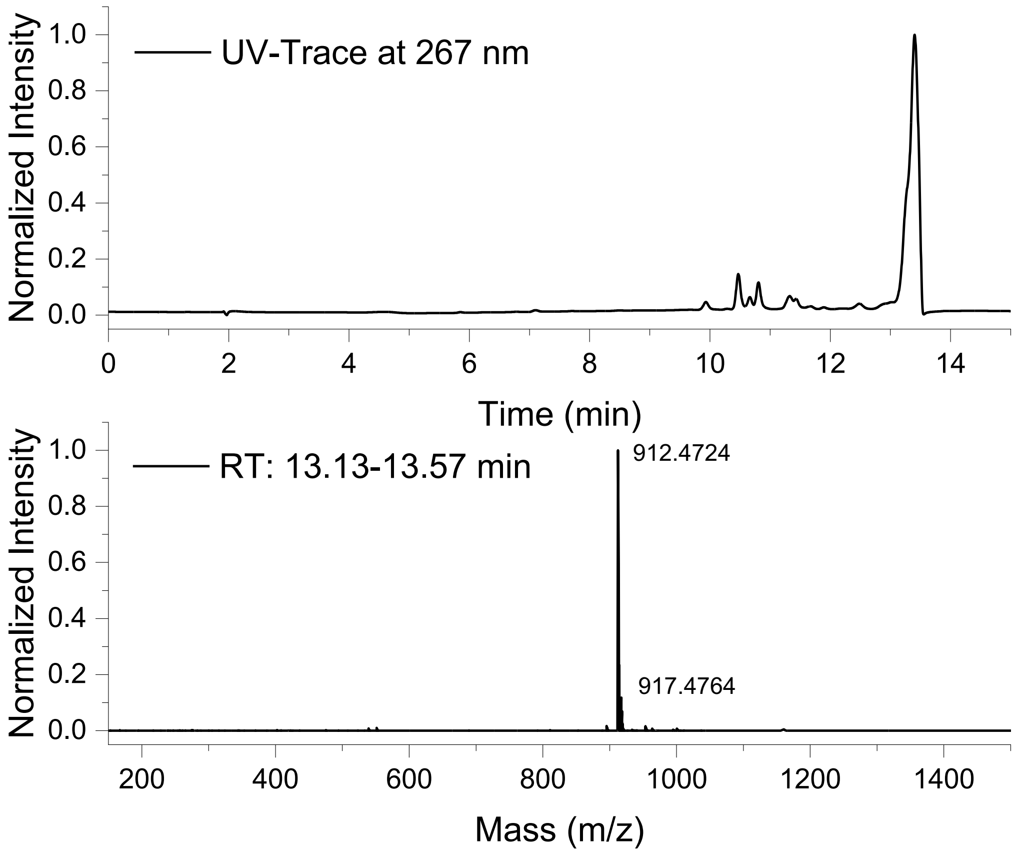

*Figure S3: LCMS of **AS**.*

LCMS: Calculated for $C_{53}H_{66}O_{10}SNa^{+}$: 917.4269; found: 917.4264

Calculated for $C_{53}H_{66}O_{10}SNH_4^{+}$: 912.4715; found: 912.4724

4.2 AS'

The more hydrophilic coumarin derivative **C1** was synthesised following a literature known procedure. **C1** was then substituted to the 7-((11-hydroxyundecyl)oxy)-2H-chromen-2-one in the previously reported procedure to obtain **AS2.**

C1

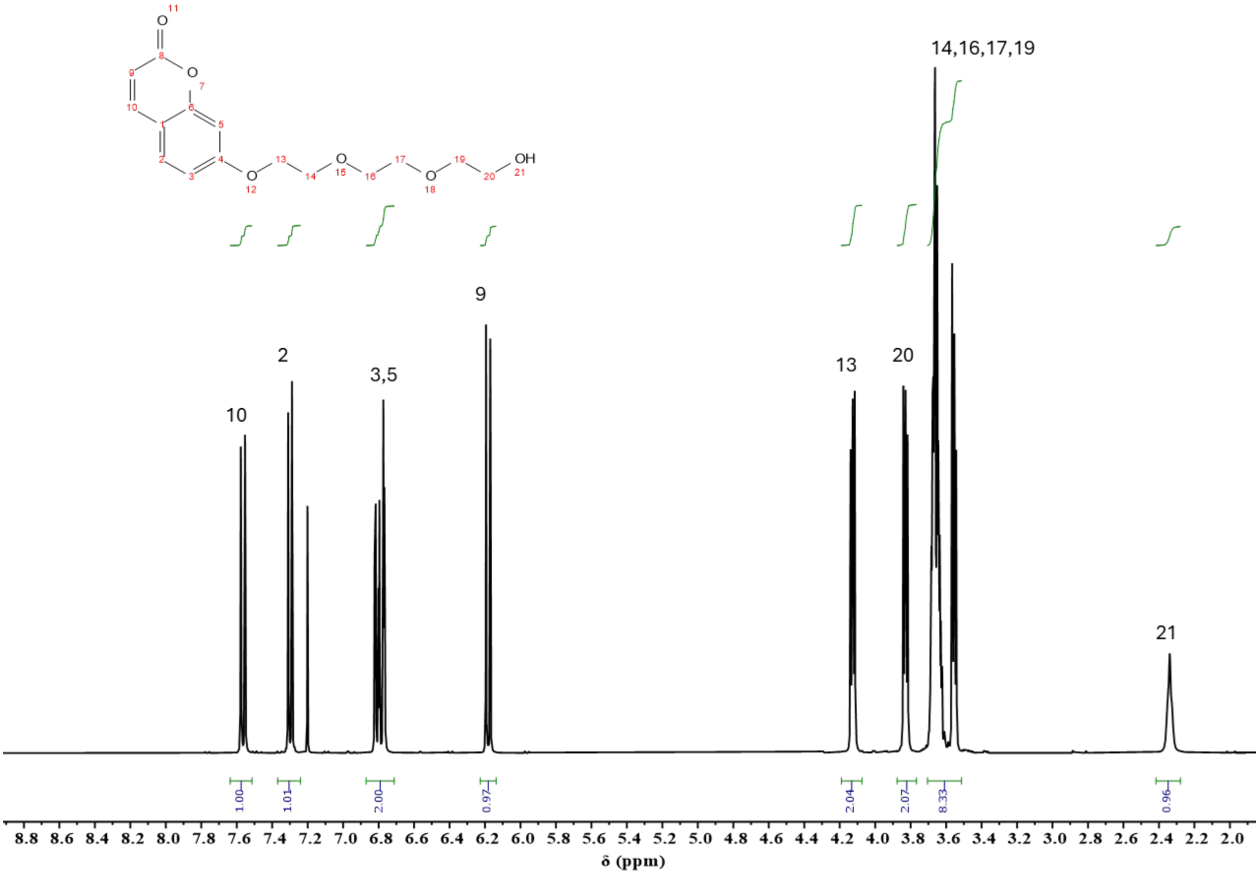

*Figure S4: ¹H-NMR of **C1** in Chloroform-d₃*

¹H NMR (400 MHz, Chloroform) *δ* 7.56 (d, *J* = 9.5 Hz, 1H), 7.30 (d, *J* = 8.6 Hz, 1H), 6.86 – 6.73 (m, 2H), 6.18 (d, *J* = 9.5 Hz, 1H), 4.19 – 4.07 (m, 2H), 3.88 – 3.77 (m, 2H), 3.75 – 3.60 (m, 6H), 3.60 – 3.53 (m, 2H), 2.35 (d, *J* = 5.3 Hz, 1H).

AS’

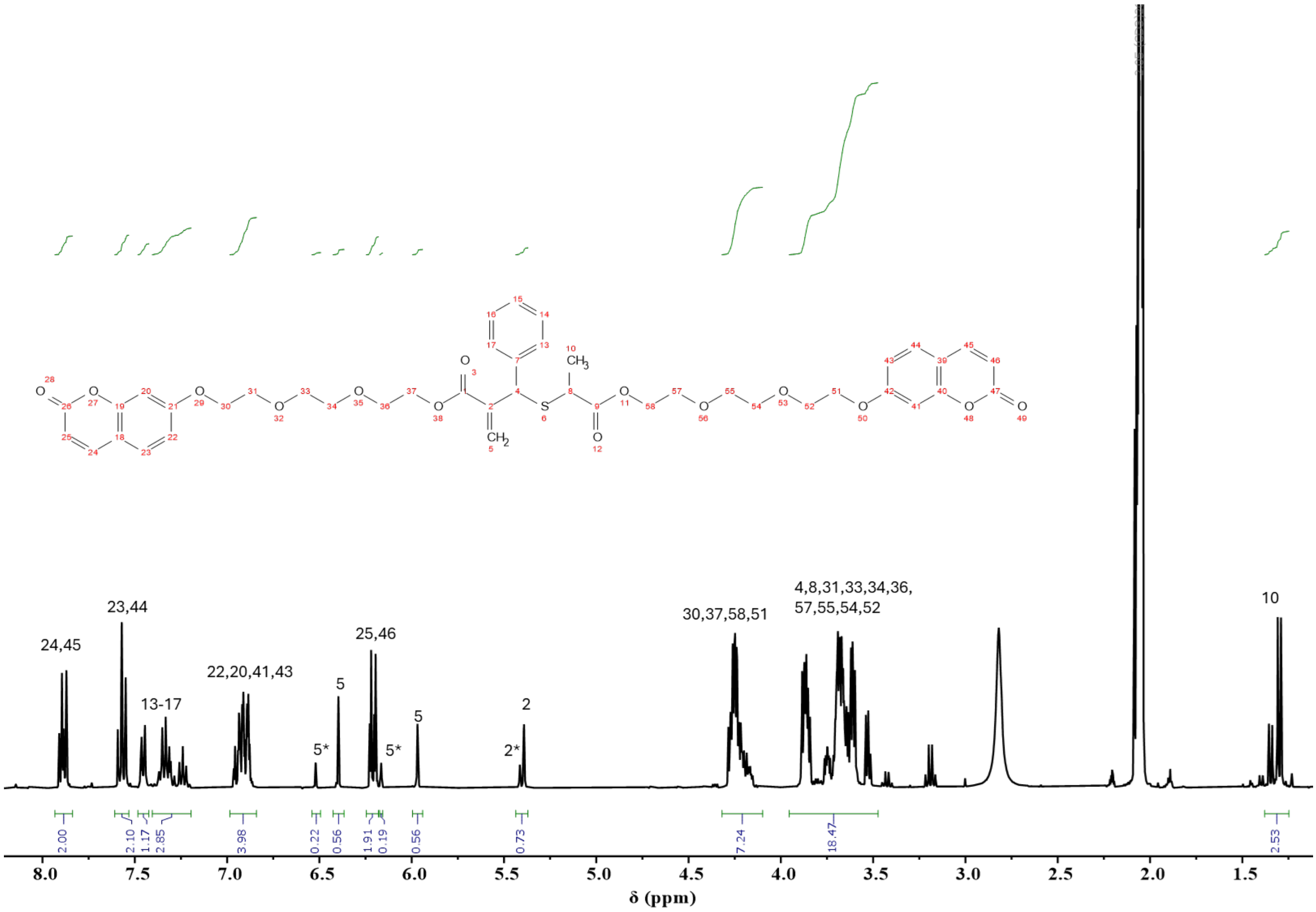

*Figure S5: ^{1}H-NMR of **AS’** in Acetone-d_6*

^{1}H NMR (400 MHz, Acetone) δ 7.91 (dd, J = 9.5, 5.9 Hz, 2H), 7.59 (t, J = 8.5 Hz, 2H), 7.51 – 7.46 (m, 1H), 7.41 – 7.31 (m, 2H), 7.29 – 7.22 (m, 1H), 7.00 – 6.86 (m, 4H), 6.42 (s, 1H), 6.23 (dd, J = 9.5, 3.0 Hz, 2H), 5.99 (d, J = 1.0 Hz, 1H), 5.41 (s, 1H), 4.35 – 4.12 (m, 8H), 3.96 – 3.52 (m, 18H), 1.32 (d, J = 7.2 Hz, 3H).

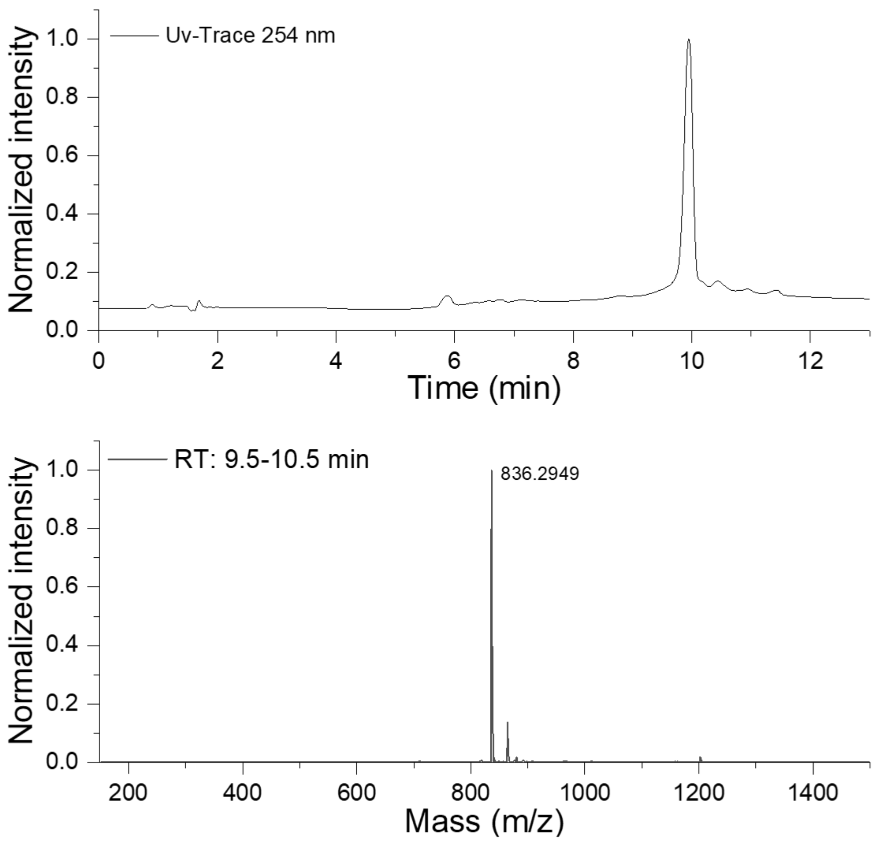

*Figure S6: LCMS of **AS'***

LCMS: Calculated for $C_{43}H_{50}NO_{14}S^{+}$: 836.2947; found: 836.2949

4.3 M

A 2 mg/mL solution of **AS** in acetonitrile was pumped through a photoflow-reactor equipped with an LED with a peak emission at λ = 365 nm. A flow rate of 100 µL/min resulted in an exposure time of 50 min.

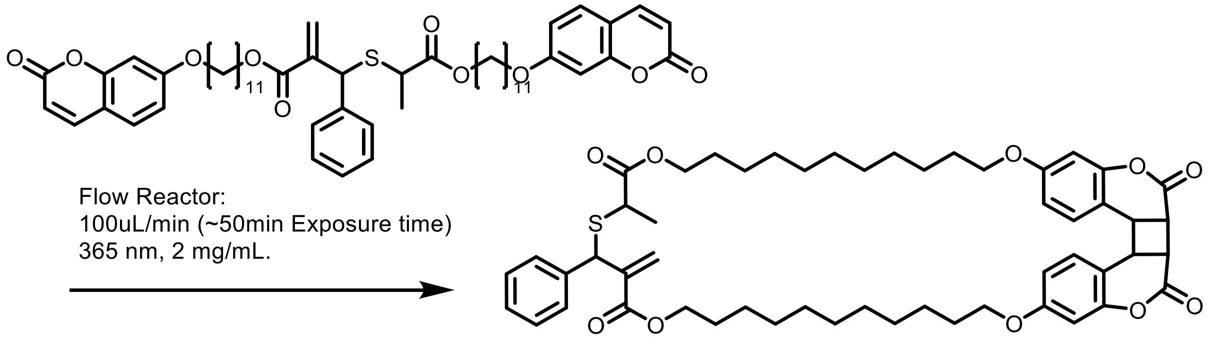

*Scheme S1: Reaction scheme for the [2+2] photocycloaddition of **AS** to **M**.*

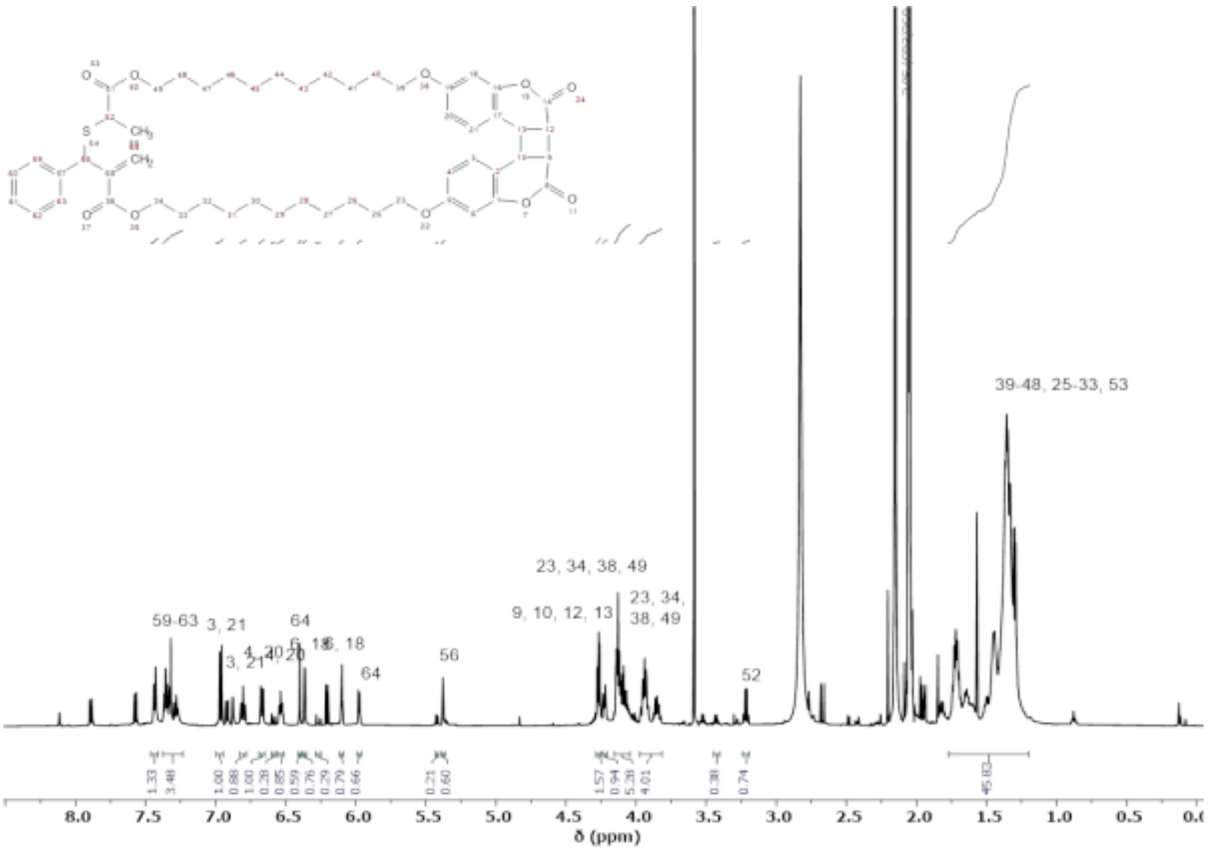

*Figure S7: ^{1}H-NMR of **M** in Acetone-d_6.*

^{1}H NMR (600 MHz, Acetone) δ 7.46 – 7.23 (m, 5H), 6.96 (d, J = 8.6 Hz, 1H), 6.83 – 6.79 (m, 1H), 6.67 (ddt, J = 8.6, 2.2, 1.0 Hz, 1H), 6.61 – 6.50 (m, 1H), 6.40 (d, J = 0.8 Hz, 1H), 6.36 (dd, J = 2.5, 1.2 Hz, 1H), 6.10 (q, J = 1.9 Hz, 1H), 6.00 – 5.95 (m, 1H), 5.38 (s, 1H), 4.30 – 4.24 (m, 4H), 4.15 – 4.05 (m, 4H), 3.98 – 3.81 (m, 4H), 3.25 – 3.19 (m, 1H), 1.77 – 1.19 (m, 53H).

4.4 M’

A solution of **AS2** in acetonitrile 2 mg/mL was irradiated with UVA lamps. The reaction mixture was concentrated by a rotary-evaporator and purified with reverse phase chromatography.

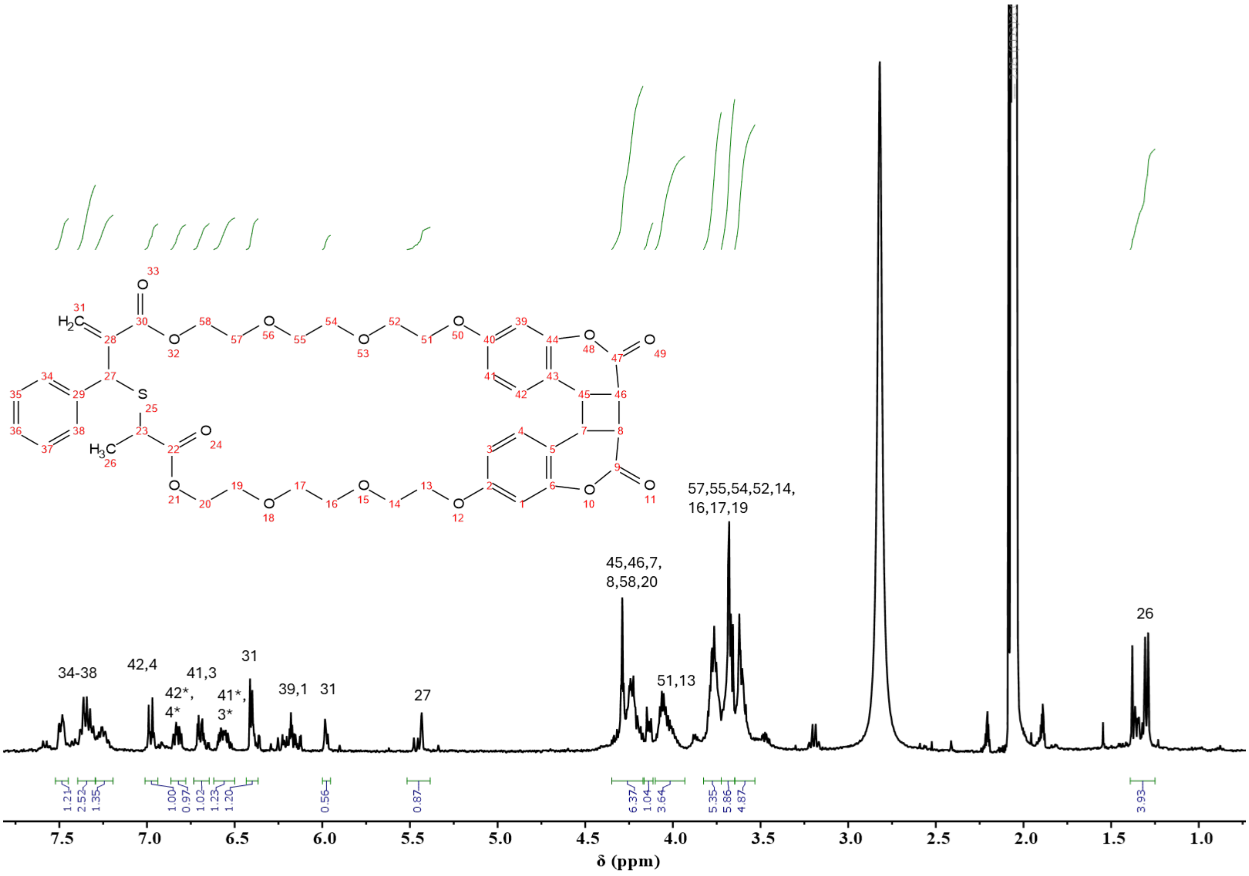

*Figure S 8: ^{1}H-NMR of **M’** in Acetone-d_6.*

^{1}H NMR (400 MHz, Acetone) δ 7.53 – 7.45 (m, 1H), 7.43 – 7.20 (m, 3H), 6.98 (dd, J = 8.6, 3.1 Hz, 1H), 6.88 – 6.79 (m, 1H), 6.73 – 6.63 (m, 1H), 6.62 – 6.51 (m, 1H), 6.45 – 6.36 (m, 1H), 6.29 – 6.11 (m, 1H), 5.98 (d, J = 5.7 Hz, 1H), 5.46 (d, J = 17.3 Hz, 1H), 4.38 – 4.16 (m, 7H), 4.16 – 4.11 (m, 1H), 4.11 – 3.94 (m, 4H), 3.85 – 3.73 (m, 5H), 3.71 – 3.65 (m, 6H), 3.64 – 3.56 (m, 5H), 1.40 – 1.25 (m, 3H).

5 Polymerisations

5.1 ZnHTPP

Zinc acetate (121.6 mg, 3 eq.) and 4,4',4'',4'''-(porphyrin-5,10,15,20-tetrayl)tetraphenol (678.8 mg, 1 eq.) were dissolved in 5 mL DMF and heated to 50 °C for 3 h. After cooling the mixture down, the product was precipitated out of cold water, centrifuged and used without any further purification.

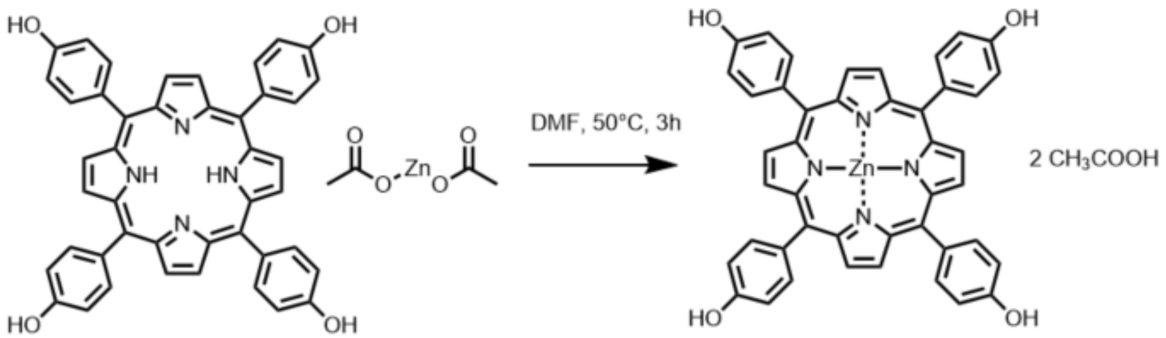

Scheme S2: Reaction Scheme for the synthesis of ZnHTPP.

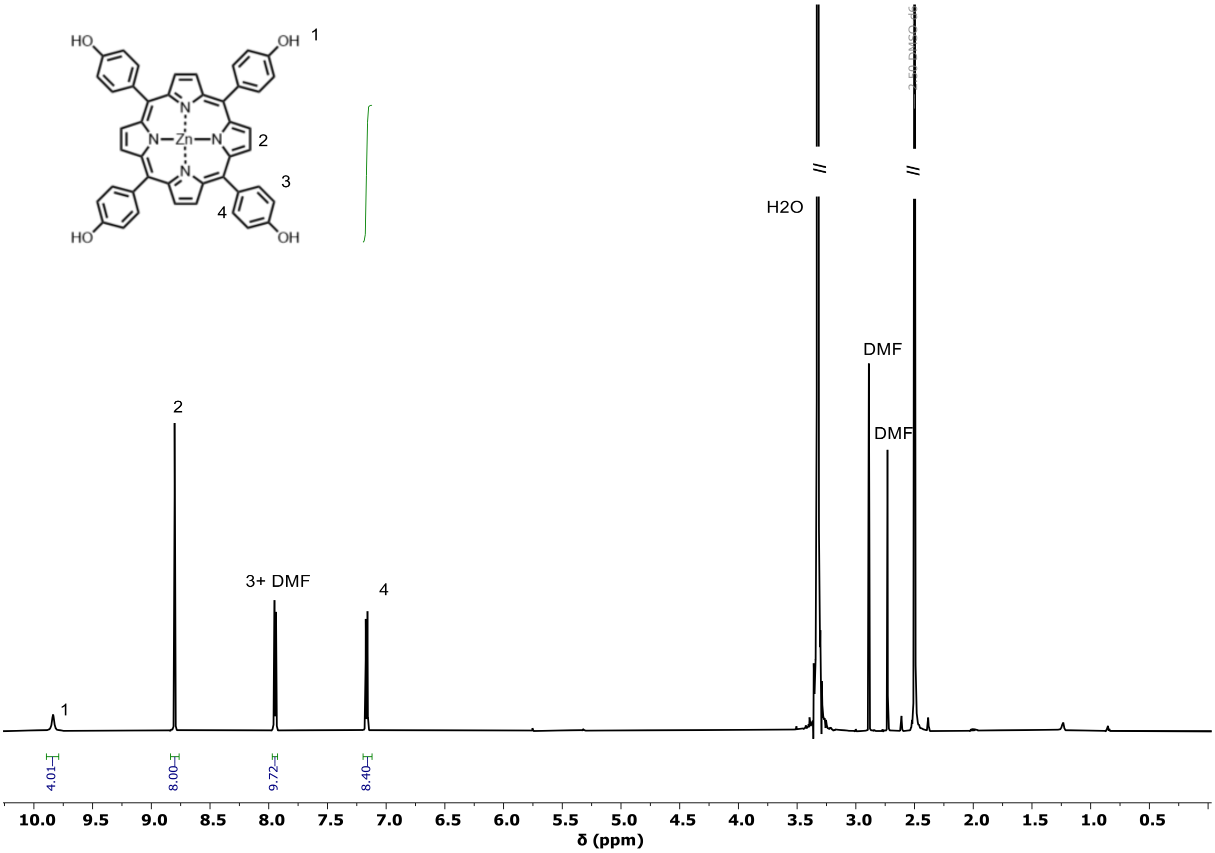

Figure S9: ^{1}H-NMR of ZnHTPP in DMSO-d_6.

^{1}H NMR (600 MHz, DMSO) δ 9.84 (s, 4H), 8.80 (s, 8H), 8.05 – 7.83 (m, 8H), 7.27 – 7.08 (m, 8H).

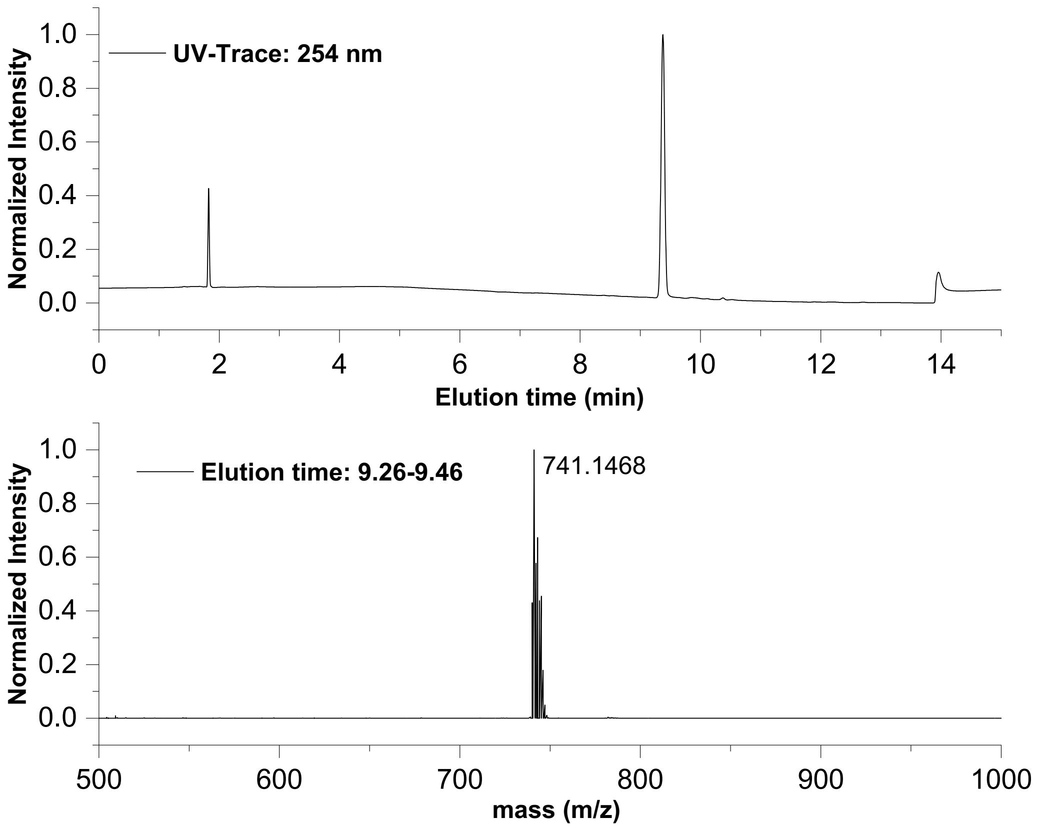

Figure S10: LCMS of ZnHTPP.

LCMS: Calculated for: $C_{44}H_{28}N_4O_4ZnH^+$: 741.1475; found: 741.1468

5.2 **CTA and surface functionalization**

The CTA 2-(((ethylthio)carbonothioyl)thio)propanoic acid was synthesized as previously reported[3] and further modified to enable surface attachment on silicon wafers:

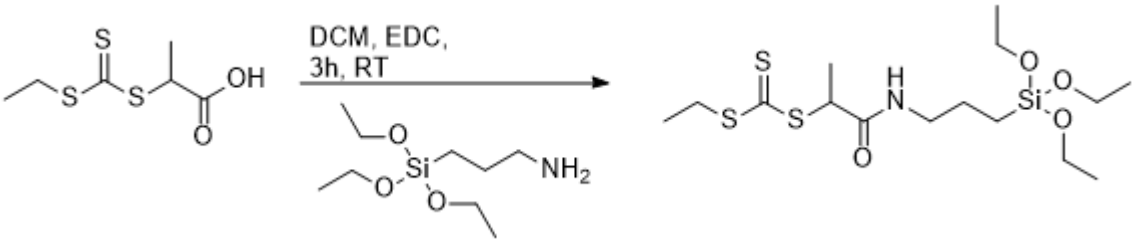

Scheme S3: Reaction Scheme for the CTA modification to obtain ethyl (1-oxo-1-((3-(triethoxysilyl)propyl)amino)propan-2-yl) carbonotrithioate.

A dry Schlenk flask was evacuated and purged with Argon several times before 175.4 mg (0.79 mmol, 1.0 eq.) 3-(triethoxysilyl)propan-1-amine, 200.0 mg 2-(((ethylthio)carbonothioyl)thio)propanoic acid (0.95 mmol, 1.2 eq.), 227.9 mg (1.19 mmol, 1.5 eq.) 1-Ethyl-3-(3-dimethylaminopropyl)carbodiimide and 15mL of dry DCM were added. The mixture was stirred at room temperature for 3 h and then washed with sodium bicarbonate (2x), water (2x) and brine (2x). The organic layer was dried over magnesiumsulfate. DCM was removed under reduced pressure to obtain a yellow oil in 88% yield.

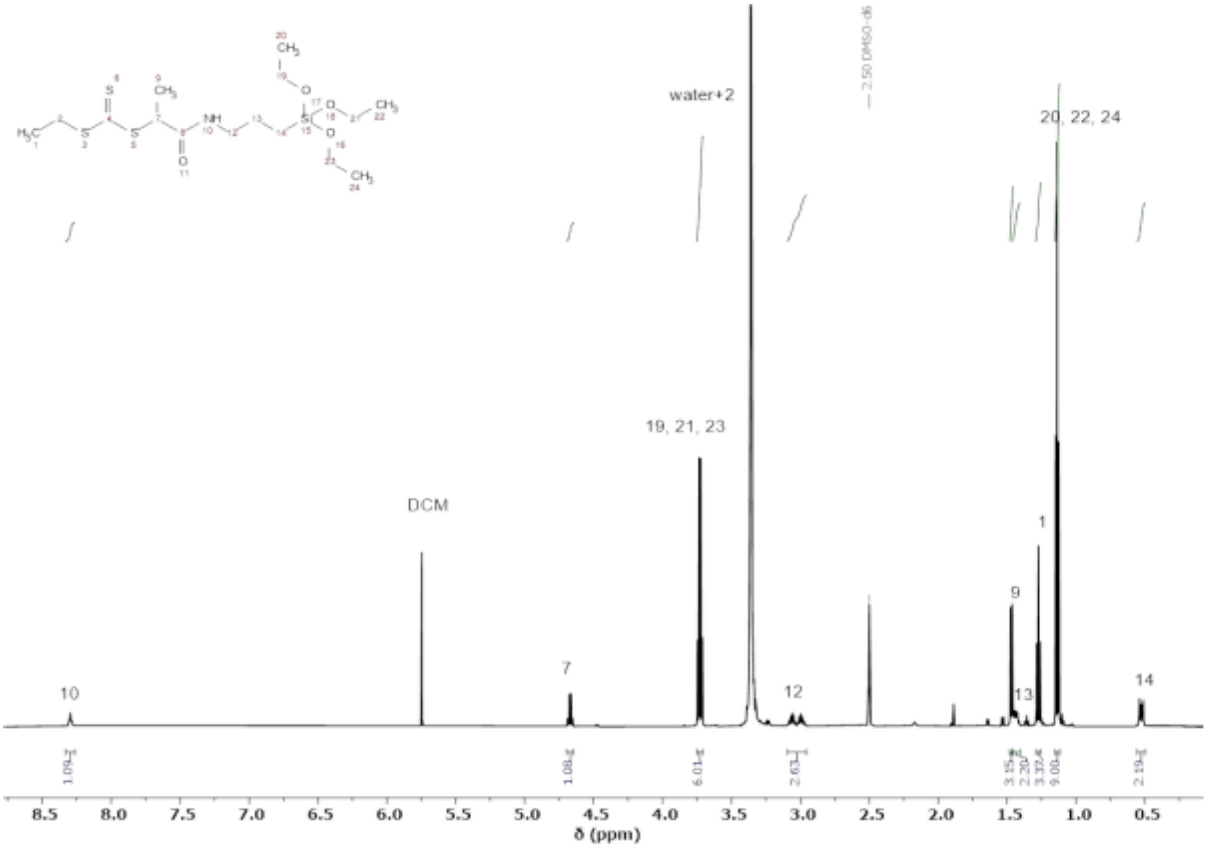

Figure S11: ^{1}H-NMR of ethyl (1-oxo-1-((3-(triethoxysilyl)propyl)amino)propan-2-yl) carbonotrithioate in DMSO-d_6.

^{1}H NMR (600 MHz, DMSO) *δ* 8.30 (s, 1H), 4.67 (d, *J* = 7.0 Hz, 1H), 3.73 (q, *J* = 7.0 Hz, 6H), 3.17 – 2.91 (m, 2H), 1.47 (d, *J* = 7.0 Hz, 3H), 1.46 – 1.40 (m, 2H), 1.30 – 1.24 (m, 3H), 1.14 (t, *J* = 7.0 Hz, 9H), 0.59 – 0.47 (m, 2H).

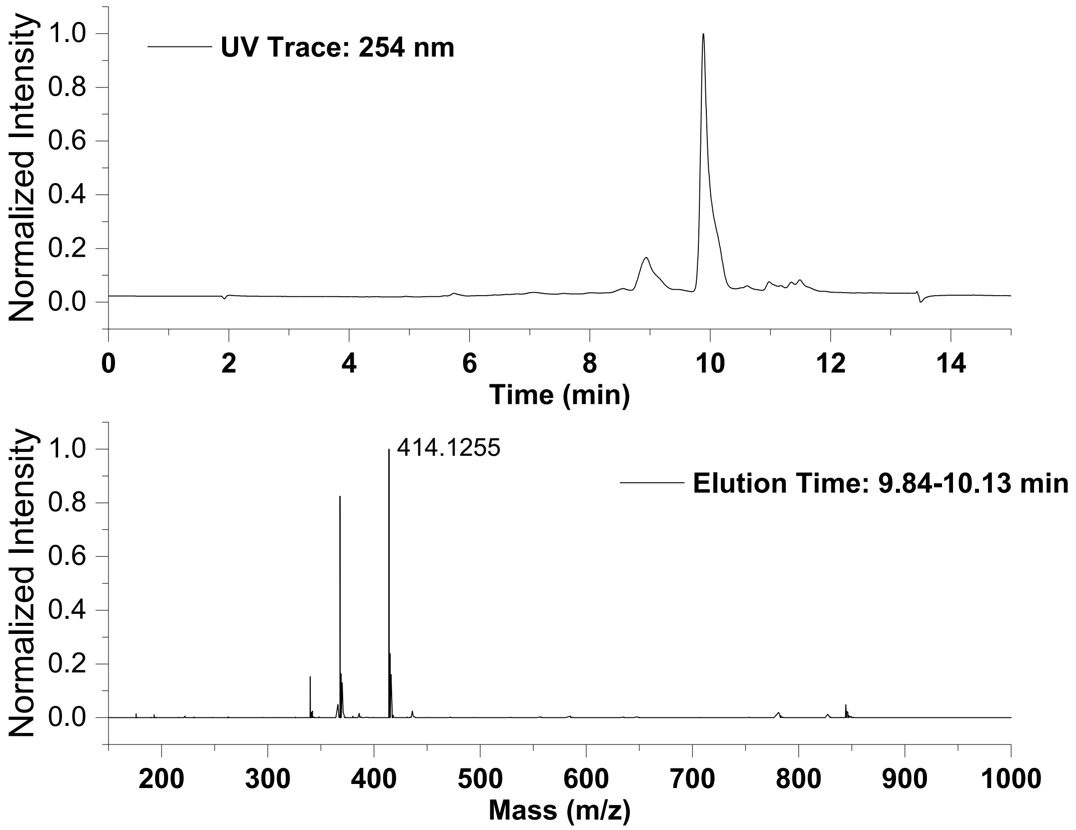

Figure S12: LCMS of modified CTA ethyl (1-oxo-1-((3-(triethoxysilyl)propyl)amino)propan-2-yl) carbonotrithioate.

LCMS: Calculated for: $C_{15}H_{31}NO_4S_3SiH^+$: 414.1257; found: 414.1255

Surface Functionalisation

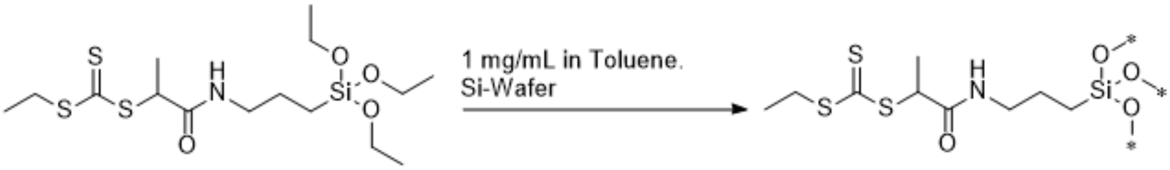

Scheme S4: Reaction scheme for the functionalisation of Si-wafers.

Small Si-wafers (ca. 10x10 mm) were plasma-cleaned for at least 20 min and subsequently immersed in a 1mg/mL solution of the modified CTA in Toluene. The mixture was heated to 50 °C for 4 h and left in solution at least over night until usage.

Large silicon wafers (for Neutron measurements; 10 cm diameter) were piranha cleaned overnight and subsequently rinsed with water and then methanol. The wafers were immersed in a 1mg/mL solution of the modified CTA in Toluene. The mixture was heated to 50 °C for 4 h and left in solution at least over night until usage.

Sum formula ethyl (1-oxo-1-((3-(triethoxysilyl)propyl)amino)propan-2-yl) carbonotrithioate after successful functionalisation: $C_9H_{16}NO_4S_3Si$.

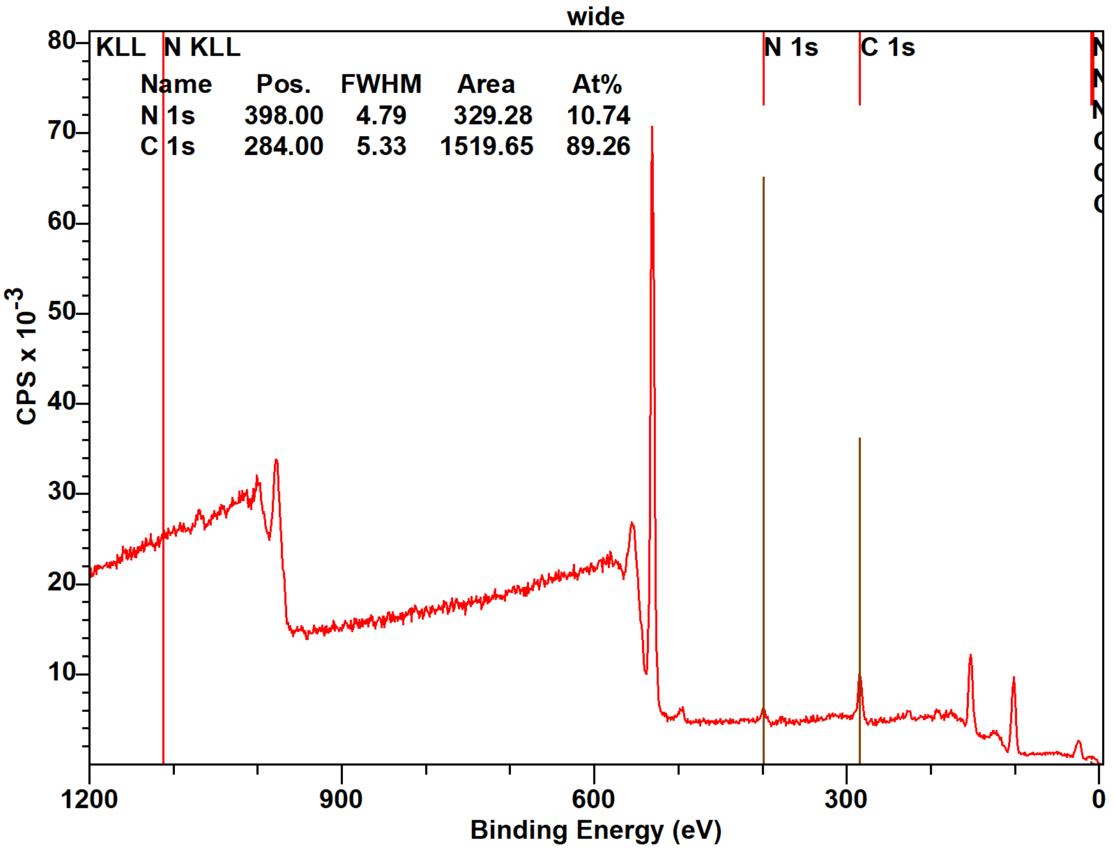

Figure S13: XPS of functionalised Si surface. The successful functionalisation was confirmed via XPS showing the 9:1 ratio of Carbon to Sulphur expected for an attached CTA (ethyl (1-oxo-1-((3-(triethoxysilyl)propyl)amino)propan-2-yl) carbonotrithioate) on the surface.

The successful functionalization was further confirmed via ToF-SIMS showing fragments of the modified CTA.

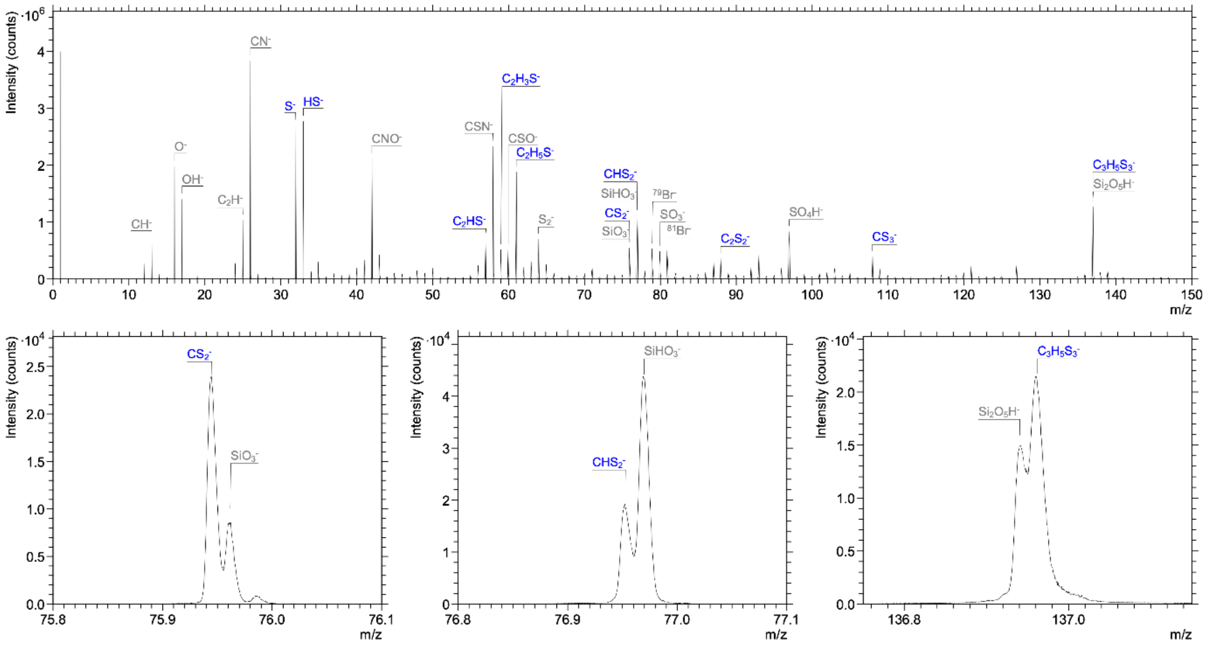

Figure S14: ToF-SIMS spectra of a CTA-functionalised Si-wafer (top). Expanded spectra of mass regions containing the CS_2^- (bottom left), CHS_2^- (bottom middle), and $C_3H_5S_3^-$ (bottom right) signals.

5.3 Polymerization setup

In solution

PET-RAFT polymerizations in solution were performed using a 96 LED-well plate with LEDs centered at λ = 572 nm by positioning a second, clear polystyrene well plate containing the polymerization mixture on top of the LED-well plate and irradiating for 12 h as reported by the Chapman group[4]:

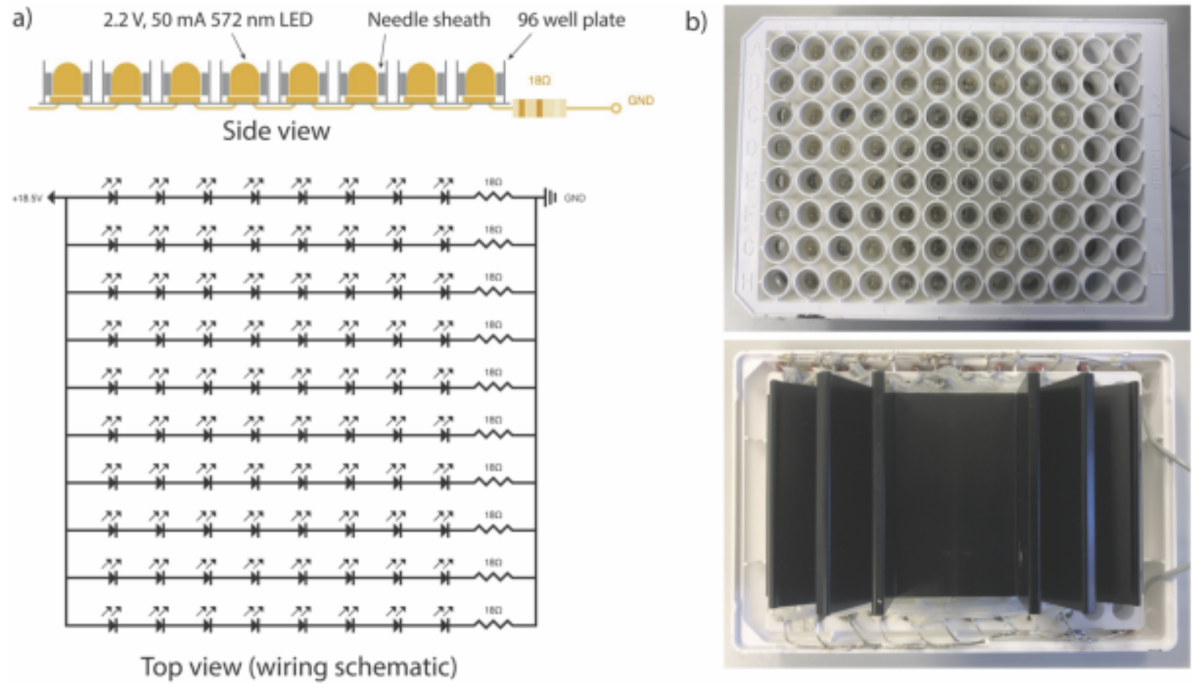

Figure S15: Wiring scheme and picture of LED-plate for PET RAFT polymerizations. a) Top and side view schematics of the LED array and b) photographs of the assembled array. Reprinted with permission from Macromolecules 2022, 55, 14, 5938-5945. Copyright 2024 American Chemical Society [4]

On surfaces

SI-PET-RAFT polymerizations on small wafers (ca. 10x10 mm) and large wafers (d = 10 cm) were performed either the same LED plate setup by turning the LED plate upside down to irradiate the silicon wafer, or alternatively by using a photoreactor with green LEDs. When performing SI-PET-RAFT polymerizations on large wafers (d = 10 cm), the polymerization was performed while the wafer was spinning on a carousel as shown in the following picture and scheme to ensure homogeneous light exposure across the whole surface. A glass slide was positioned on top of the polymerization mixture to:

- i) minimize DMSO evaporation throughout the reaction.
- ii) create a polymerization mixture layer of uniform thickness across the entire wafer.
- iii) hold the photomask in place for patterned polymer brush growth.

While the glass slide on small wafers (10 x 10 mm) adhered to the surface sufficiently, the glass slide on large wafers (d = 10 cm) had to be held in place with tape as shown in Figure S16.

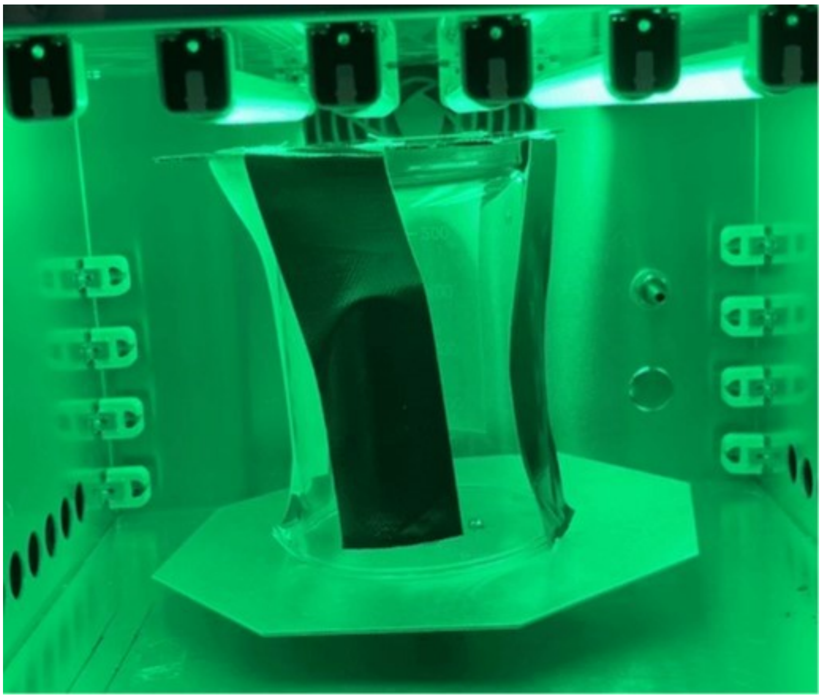

Figure S16: Picture of polymerization setup for large silicon wafers (d = 10 cm).

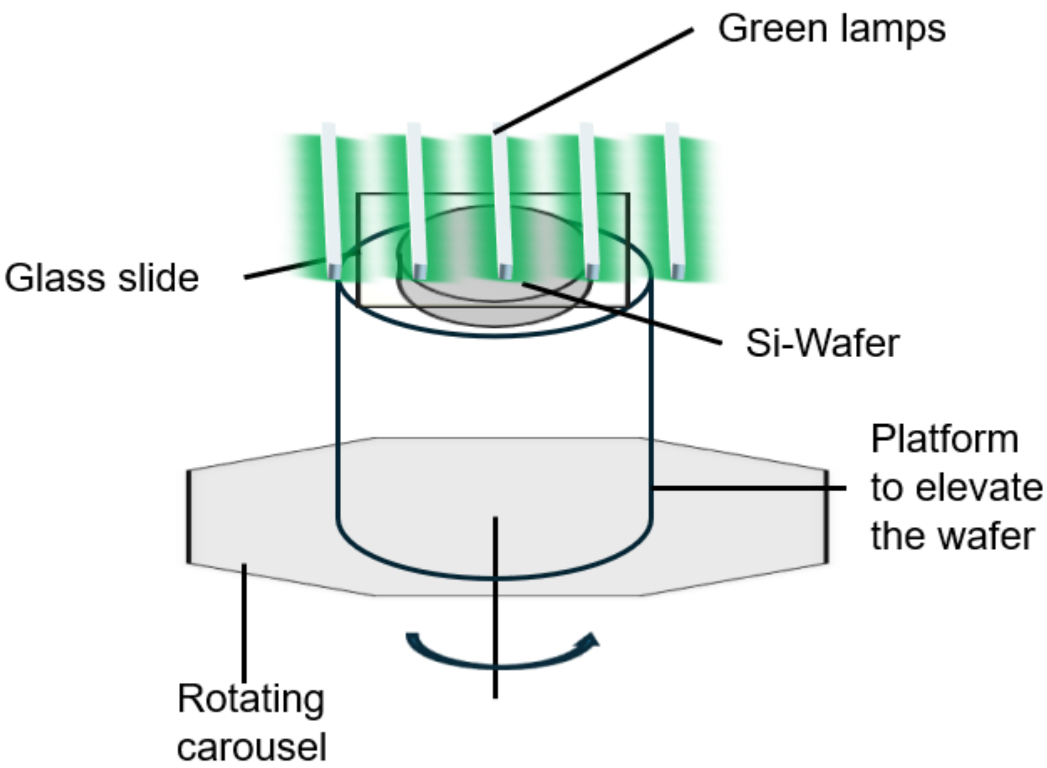

Scheme S5: Scheme of polymerization setup for large silicon wafers (d = 10 cm).

Photomasks

To obtain patterned polymer brushes, photomasks were used. These photomasks were used for polymer brush growth and degradation:

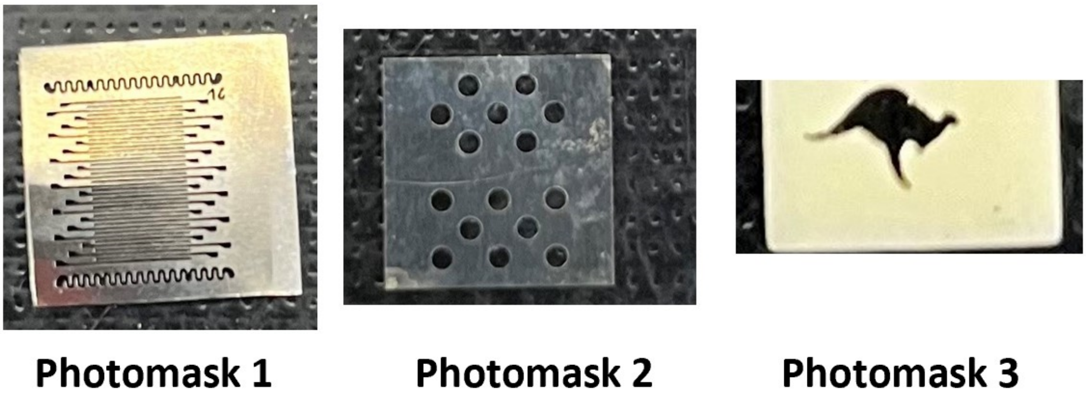

Figure S17: Photo of all used photomasks.

Photomask 3 was 3D printed from poly lactic acid.

Polymerization mixture composition

PET-RAFT in solution:

Unless otherwise noted, all PET-RAFT polymerizations were performed at 0.5 M concentration in DMSO with a ratio of 196/4/1/0.1 (DMA/**M**/CTA/ZnHTPP) by irradiating with an LED plate described in section 5.3 for 12 h.

SI-PET-RAFT on silicon:

Unless otherwise noted, all SI-PET-RAFT polymerizations were performed at 1 M concentration in DMSO with a ratio of 980/20/1/0.1 (DMA/**M**/CTA/ZnHTPP) by irradiating with an LED plate or LEDs in a photoreactor described in 5.3. This ratio does not account for surface bound CTA. Samples were irradiated for 12 h.

5.4 Patterned polymer brushes

Patterned polymer brushes were obtained by positioning a photomask on top of the wafer in addition to the procedures detailed in 5.3.

5.4.1 Standard conditions

Additional patterned polymer brushes obtained on a small (ca. 10x10 mm) wafer using the standard conditions in section 5.3 using photomask 1.

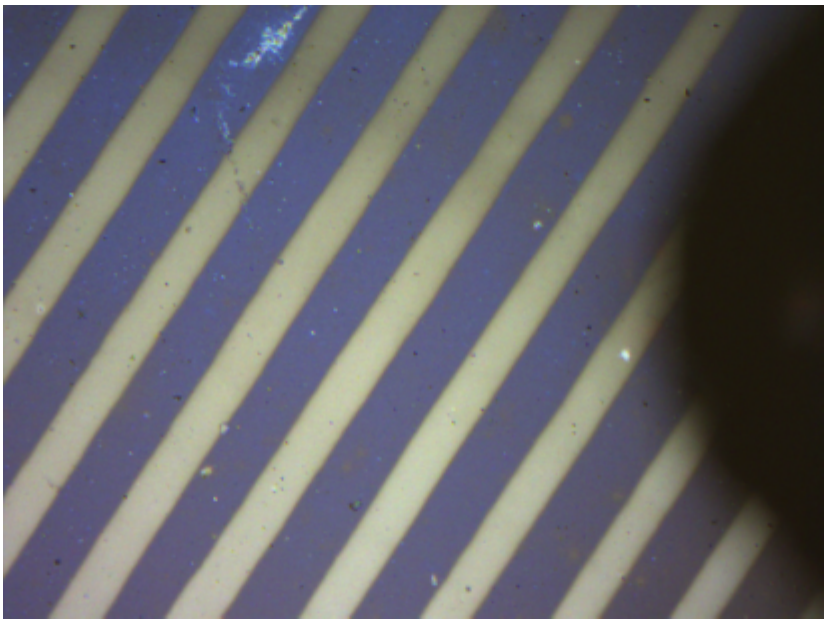

Figure S18: Microscope Image of a structured polymer brush using photomask 1.

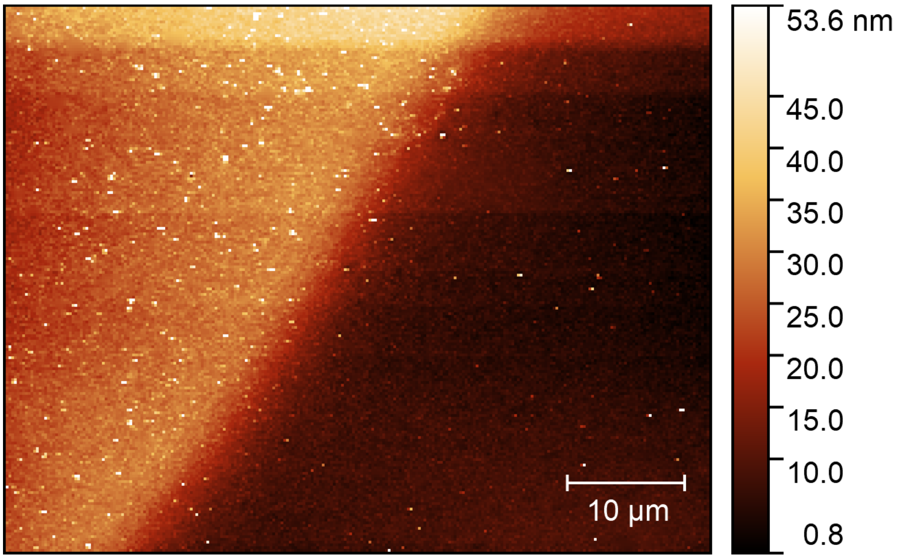

Figure S19: AFM image showing an interface between surface and polymer brush using photomask 1.

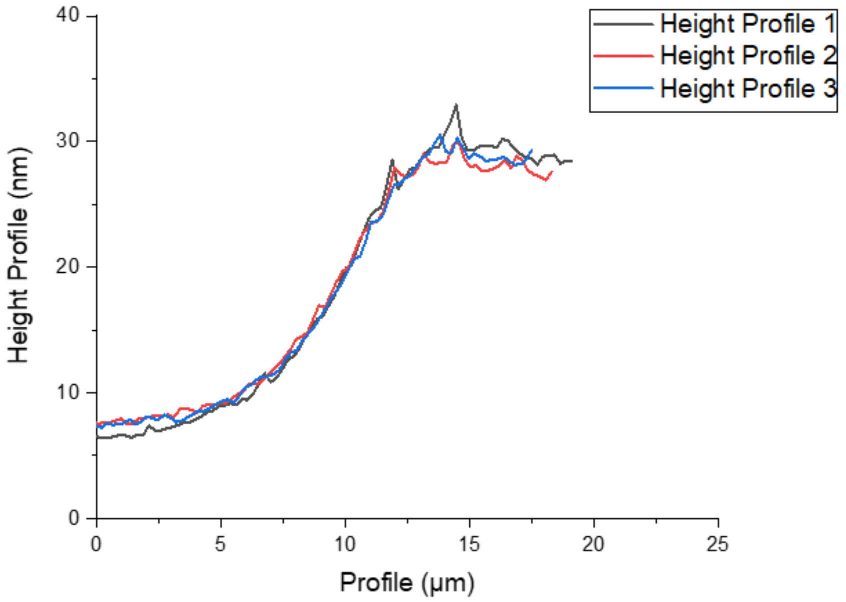

Figure S20: Height profiles showing the interface of surface to polymer brush using photomask 1.

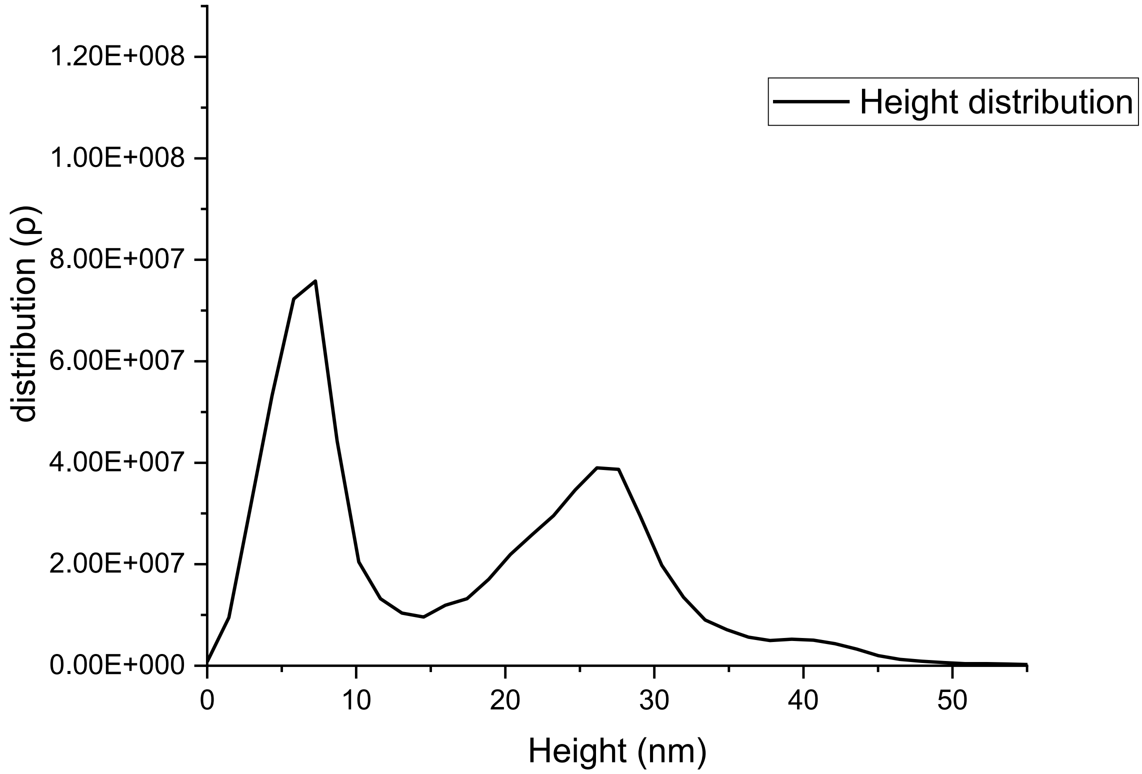

Figure S21: Height distribution of the AFM image using photomask 1.

5.4.2 0.5 M monomer solution

Polymer brushes were also obtained at lower monomer concentrations, which was halved from 1 M for standard conditions to 0.5 M in this experiment. All other parameters were kept identical to the standard conditions.

The resulting polymer brushes show a reduced dry thickness (here: 11.36 nm) indicating a dependence between monomer concentration and dry brush thickness.

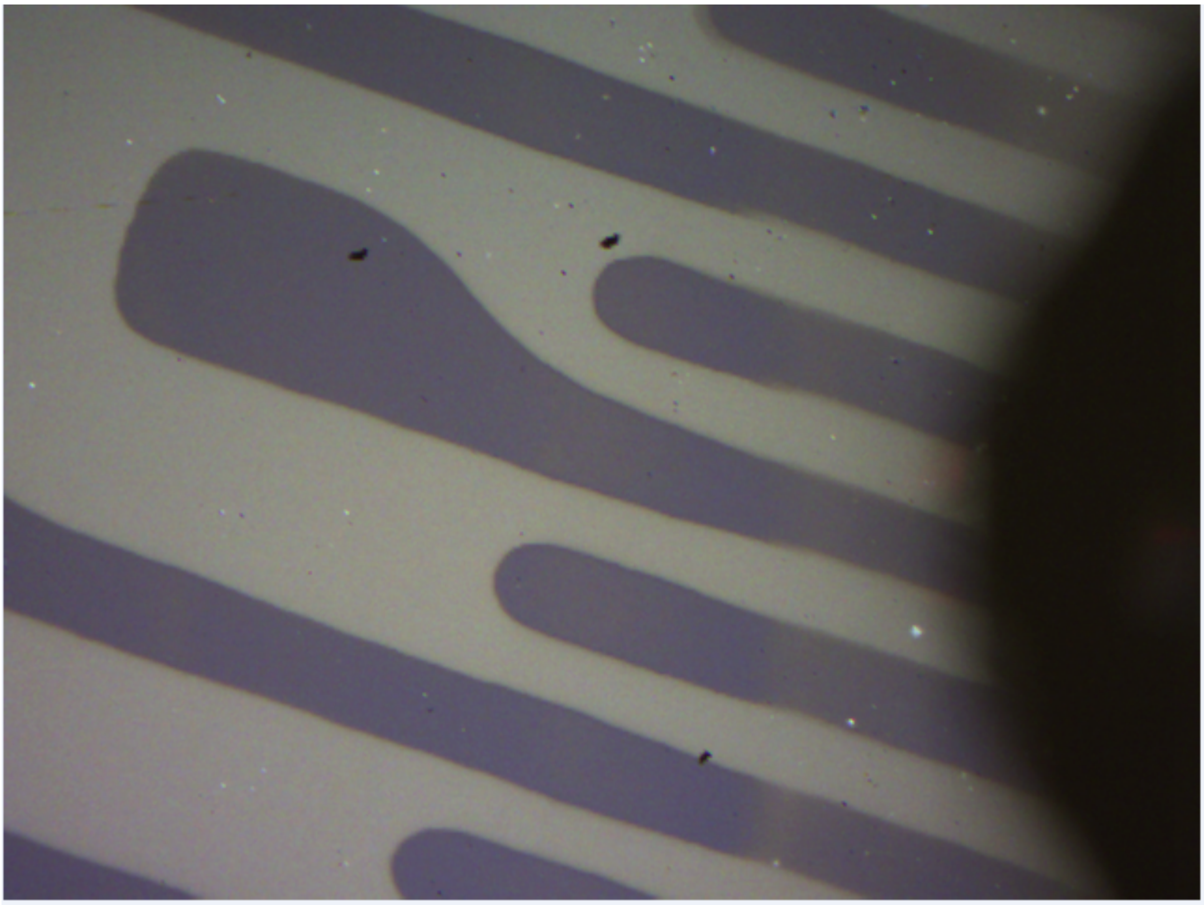

Figure S22: Microscope image of structured polymer brushes, polymerized at a combined monomer concentration of 0.5 M using photomask 1.

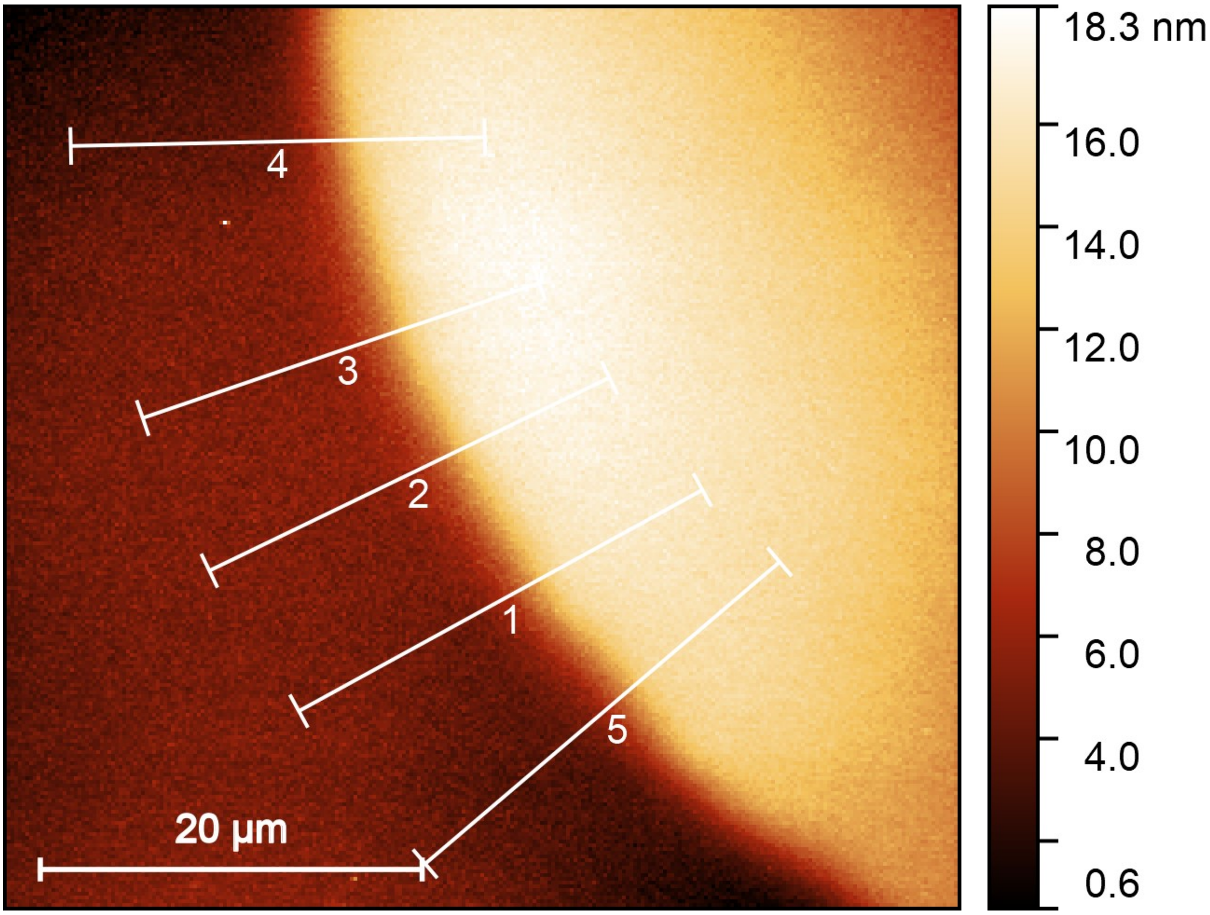

Figure S23: AFM image of surface brush interface polymerised at a combined monomer concentration of 0.5 M using photomask 1.

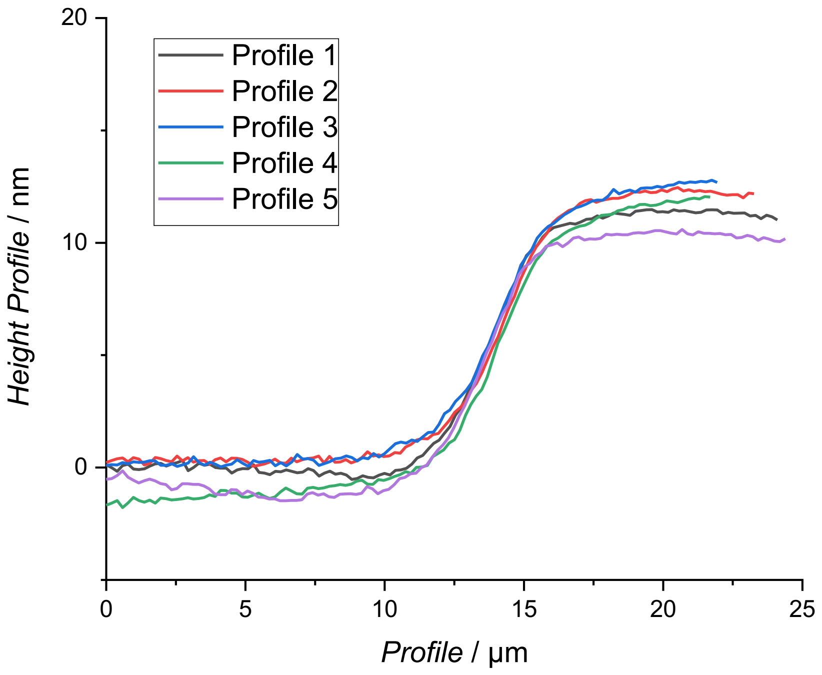

Figure S24: Height profiles of surface brush interface polymerised at a combined monomer concentration of 0.5 M using photomask 1.

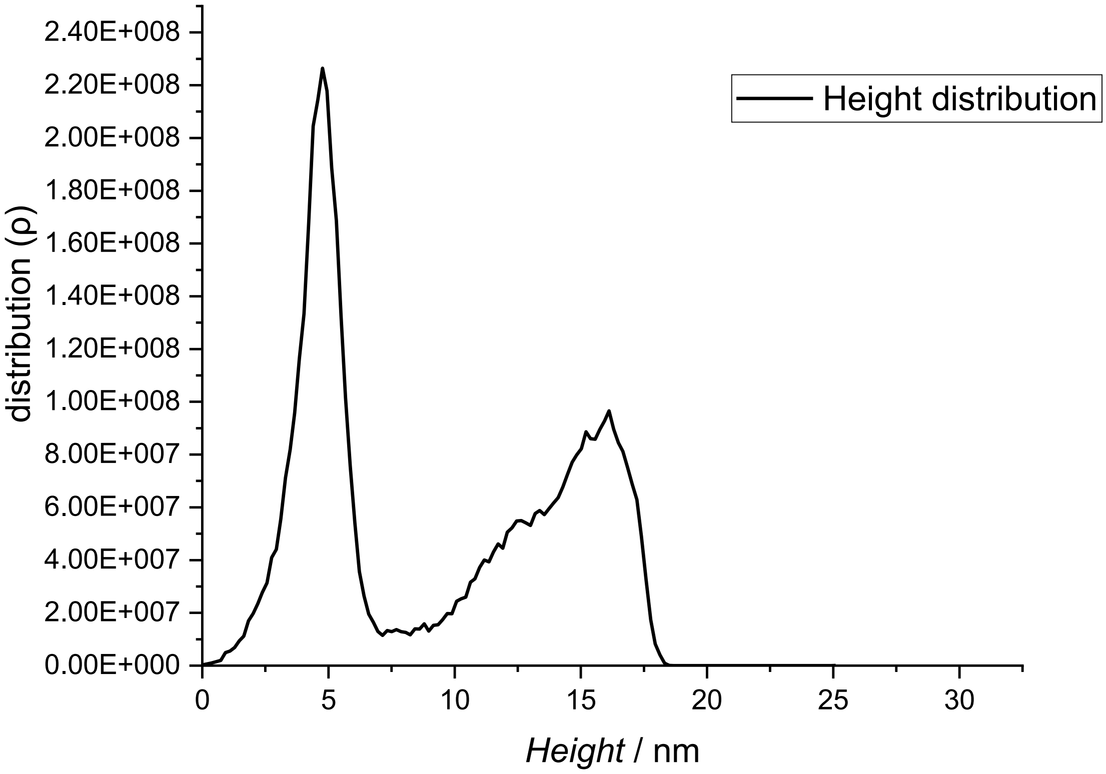

Figure S25: Height distribution of AFM image above.

5.5 AFM of scratched non-structured polymer brushes

To determine the brush height of non-patterned polymer brushes, a polymer brush was grown without photomask and scratched with the tip of pointy tweezers to remove the polymer brush and surface bound CTA. The resulting interface of brush and scratch was imaged via AFM. Thus, the height of the dry thickness of brush including the surface bound CTA can be compared to the native silicon wafer.

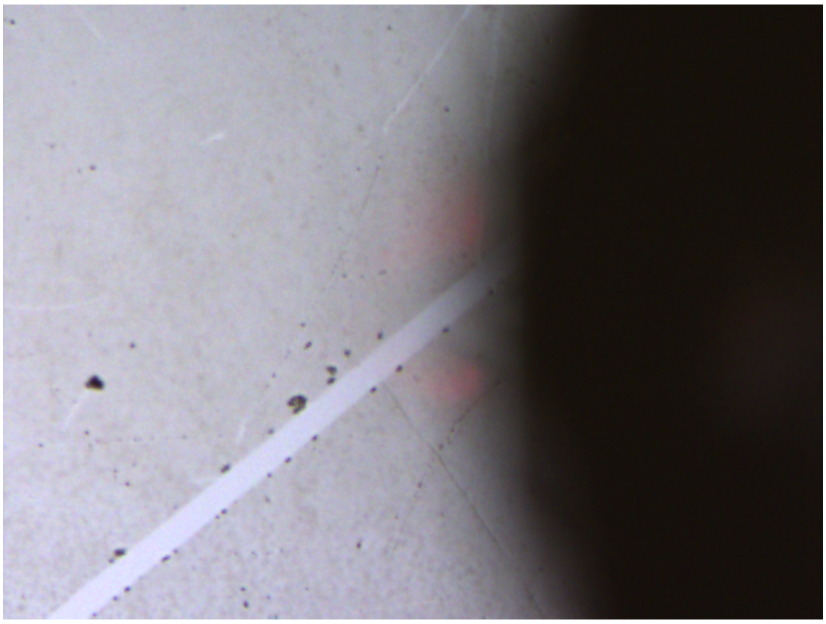

Figure S26: Microscope image of scratched surface.

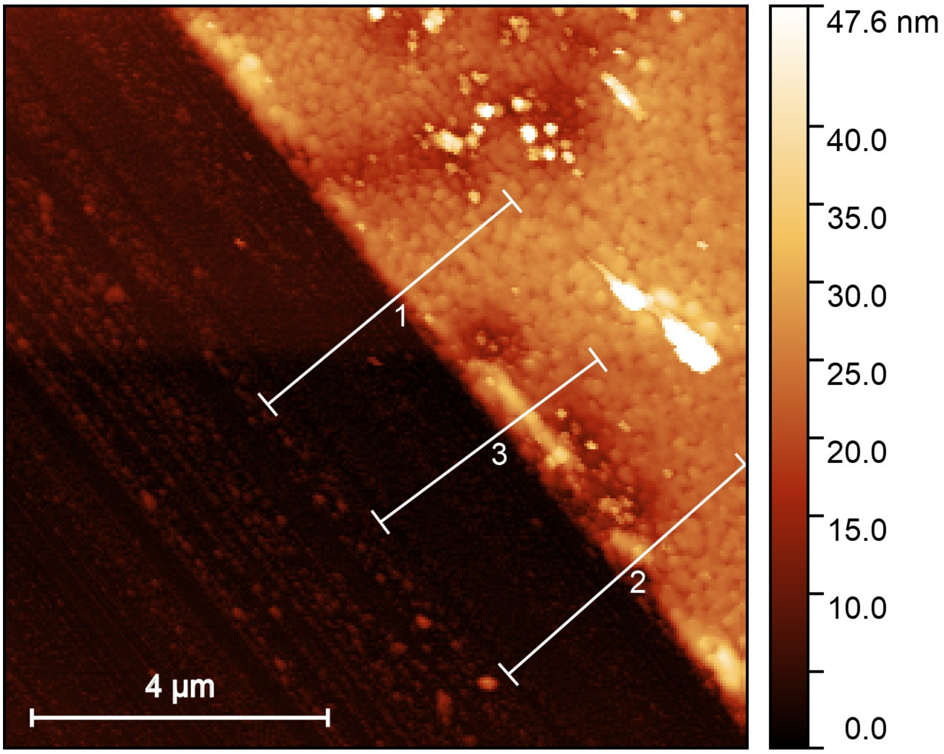

Figure S27: AFM image of scratched surface.

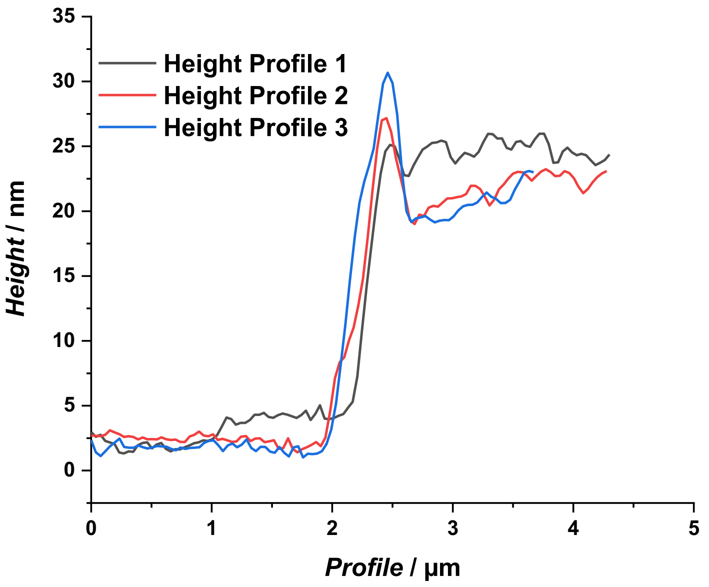

Figure S28: Height profiles of the scratch-polymer brush interface.

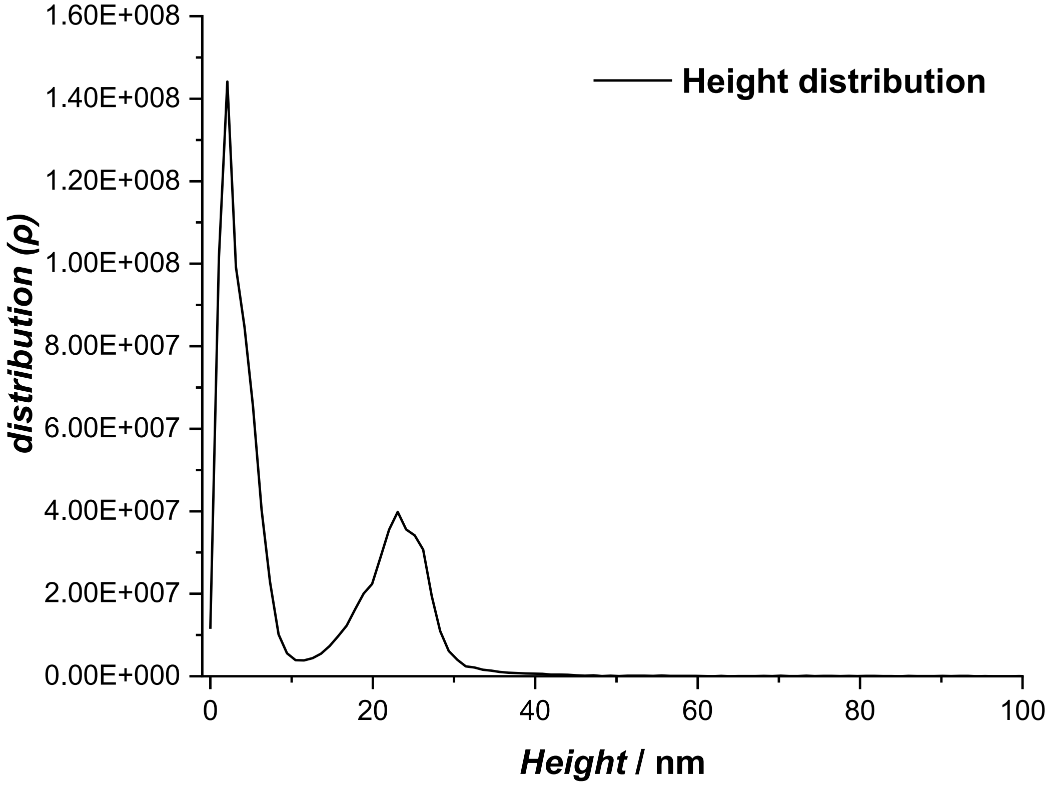

Figure S29: Height distribution of the AFM image above.

6 Photodegradation

6.1 Photodegradation setup

In solution

Photodegradations in solution were performed using UVB lamps with a peak emission of λ = 313 nm for 30 min of side irradiation.

On surface

Unless otherwise noted, all polymer brush photodegradations were performed in water with a glass slide on top of the wafer to:

i) minimize water evaporation during the reaction
ii) create a water layer of uniform thickness across the entire wafer.
iii) hold the photomask in place for patterned polymer brush degradation.

Polymer brush photodegradations on small wafers (10 x 10 mm) were performed using a λ = 325 nm LED at a distance of 3.5 cm for 12 h for slow, controlled degradation and degradation monitoring experiments. For fast degradation experiments such as patterning, UVB lamps with a peak emission of λ = 313 nm were used in a photoreactor to irradiate polymer brushes on surface for 30 min in water.

Polymer brush photodegradations on large wafers (d = 10 cm) were exclusively performed using the λ = 325 nm LED at a distance of 3.5 cm.

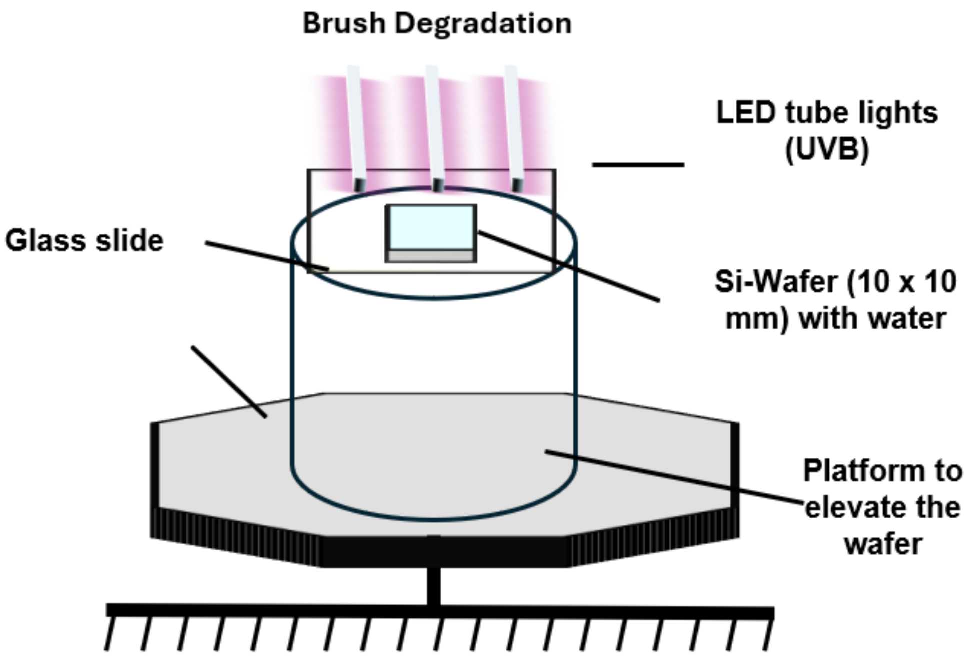

Scheme S6: Scheme of polymer brush degradation setup for fast degradation on small wafers.

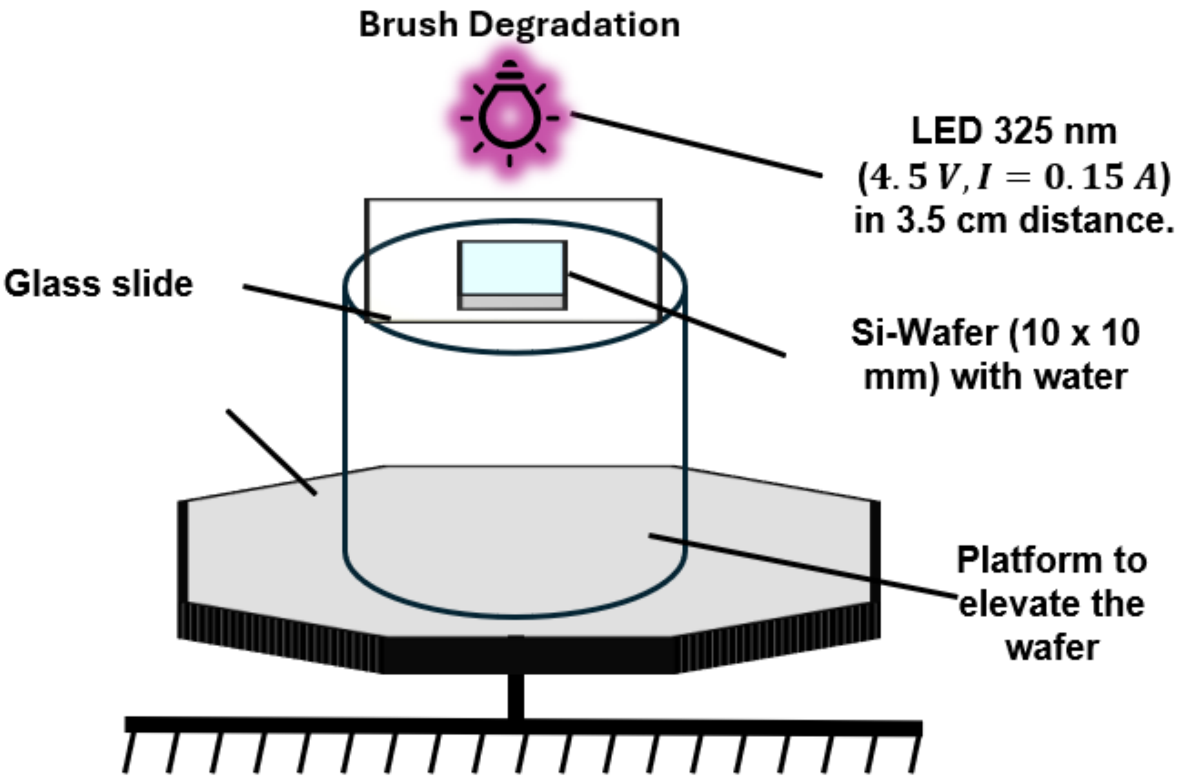

Scheme S7: Scheme for polymer brush degradation setup for slow and controlled degradation on small wafers.

6.2 Polymer photodegradation

6.2.1 Polymer photodegradation in solution

An **M**-DMA copolymer with $\overline{M_n} = 51{,}000\frac{g}{mol}$; $Đ = 1.92$ was obtained via PET RAFT polymerisation in solution using standard conditions as outlined in chapter 5.3.

The polymer degradability was initially confirmed in solution by irradiating an SEC sample of the polymer in DMAc with UVB light for 30 min. The shift in the SEC trace was monitored by injecting the sample into the SEC, performing the irradiation and reinjecting the sample into the SEC.

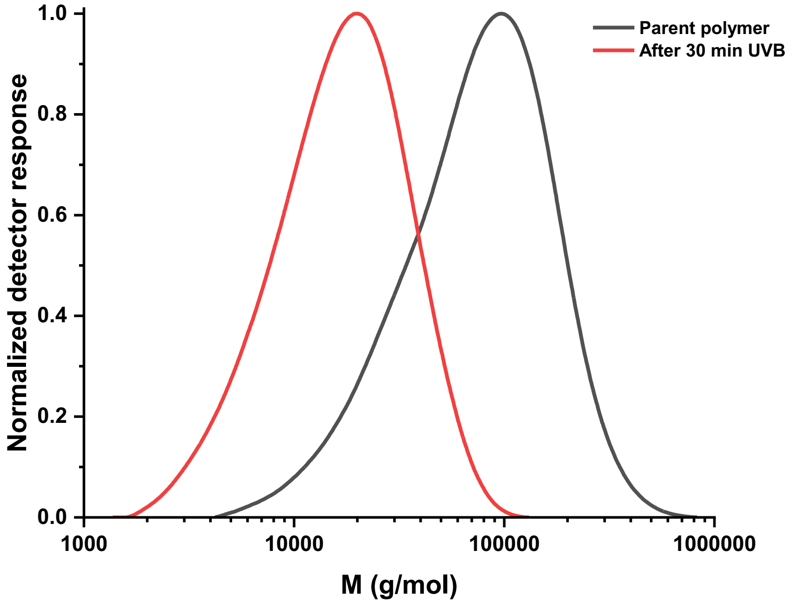

Figure S30: SEC overlay of initial and degraded M-PDMA copolymer in Dimethylacetamide.

Pristine Polymer: $\overline{M_n} = 51{,}000\frac{g}{mol}$; $Đ = 1.92$

After 30 min UVB irradiation: $\overline{M_n} = 12{,}500\frac{g}{mol}$; $Đ = 1.67$

An **M'**-DMA copolymer with $\overline{M_n} = 19{,}000\frac{g}{mol}$; $Đ = 1.63$ was obtained via PET-RAFT polymerisation in solution using standard conditions as outlined in chapter 0.

The polymer degradability was initially confirmed in solution by irradiating an SEC sample of the polymer in DMAc with UVB light for 30 min. The shift in the SEC trace was monitored by injecting the sample into the SEC, performing the irradiation and reinjecting the sample into the SEC.

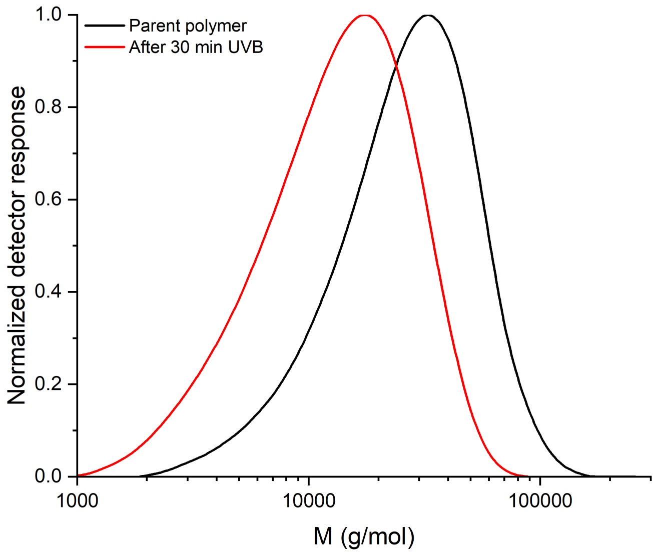

*Figure S31: SEC overlay of initial and degraded **M'**-PDMA copolymer in Dimethylacetamide.*

Pristine Polymer: $\overline{M_n} = 19{,}000\,\frac{g}{mol}$; $Đ = 1.63$

After 30 min UVB irradiation: $\overline{M_n} = 9{,}500\,\frac{g}{mol}$; $Đ = 1.73$

6.2.2 Polymer Brush Photodegradation

Kinetics

The photodegradation of polymer brushes obtained using standard conditions as outlined in 5.3 was investigated in water (all data shown in main manuscript) and monitored via ellipsometry. A degradation study containing 3.5% of **M** and 2% of the more hydrophilic **M'** are shown below. The thickness of the CTA functionalization layer was subtracted from the measured values to report relative brush thickness.

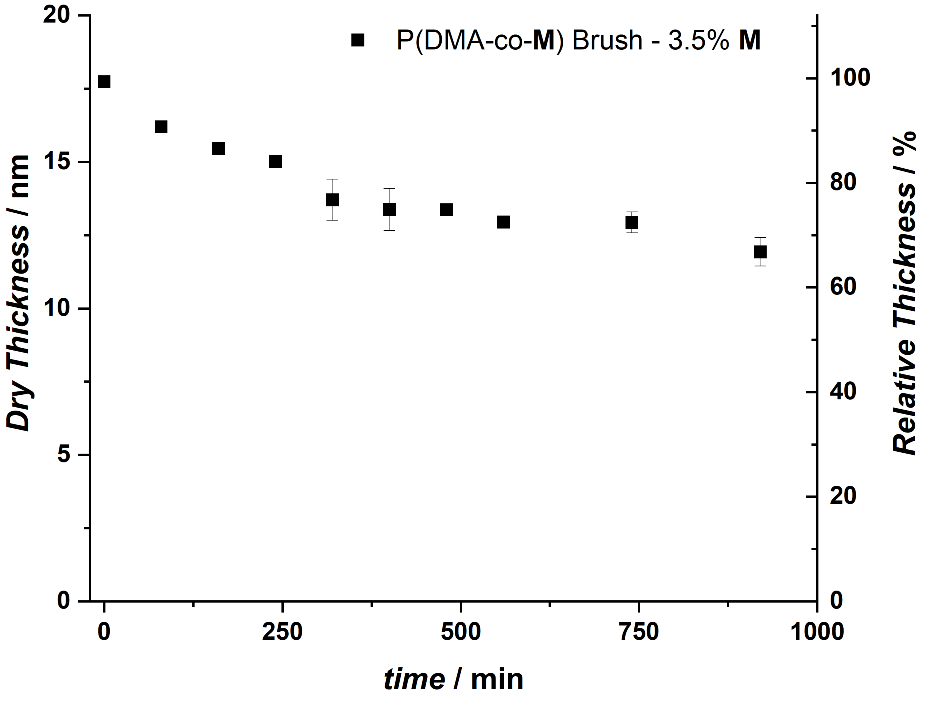

*Figure S32: Polymer brush degradation kinetics containing 3.5% of **M**.*

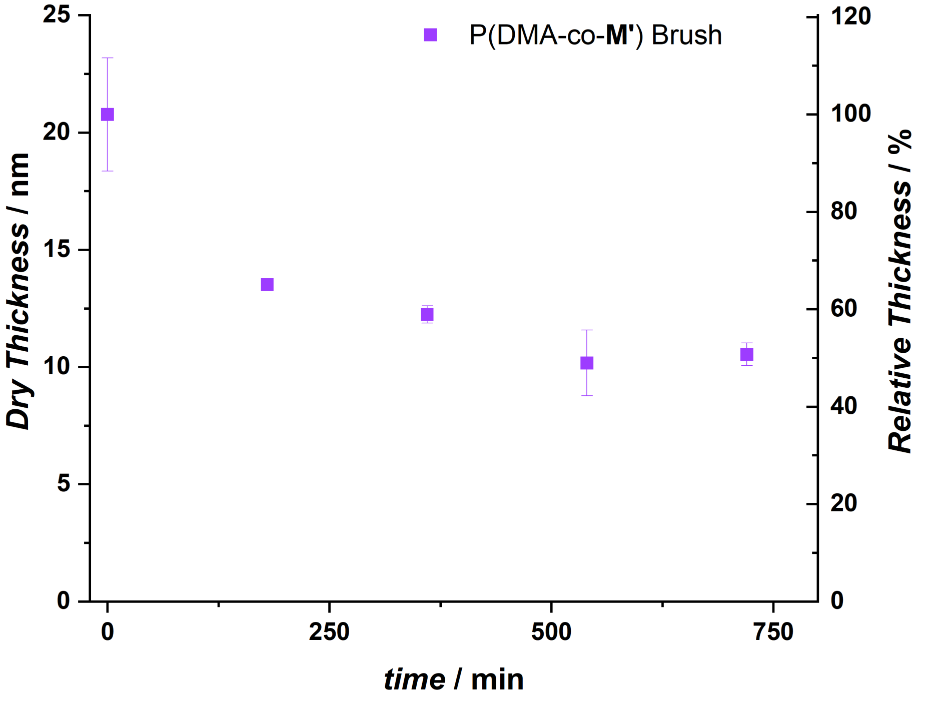

*Figure S33: Polymer brush degradation kinetics containing 2% of **M'**.*

6.2.3 XPS of Polymer Brushes

XPS of an unstructured P(DMA-*co*-**M**) brush obtained as outlined in section 5.3 was measured on the pristine brush and after UVA light photolysis in water for 600 min.

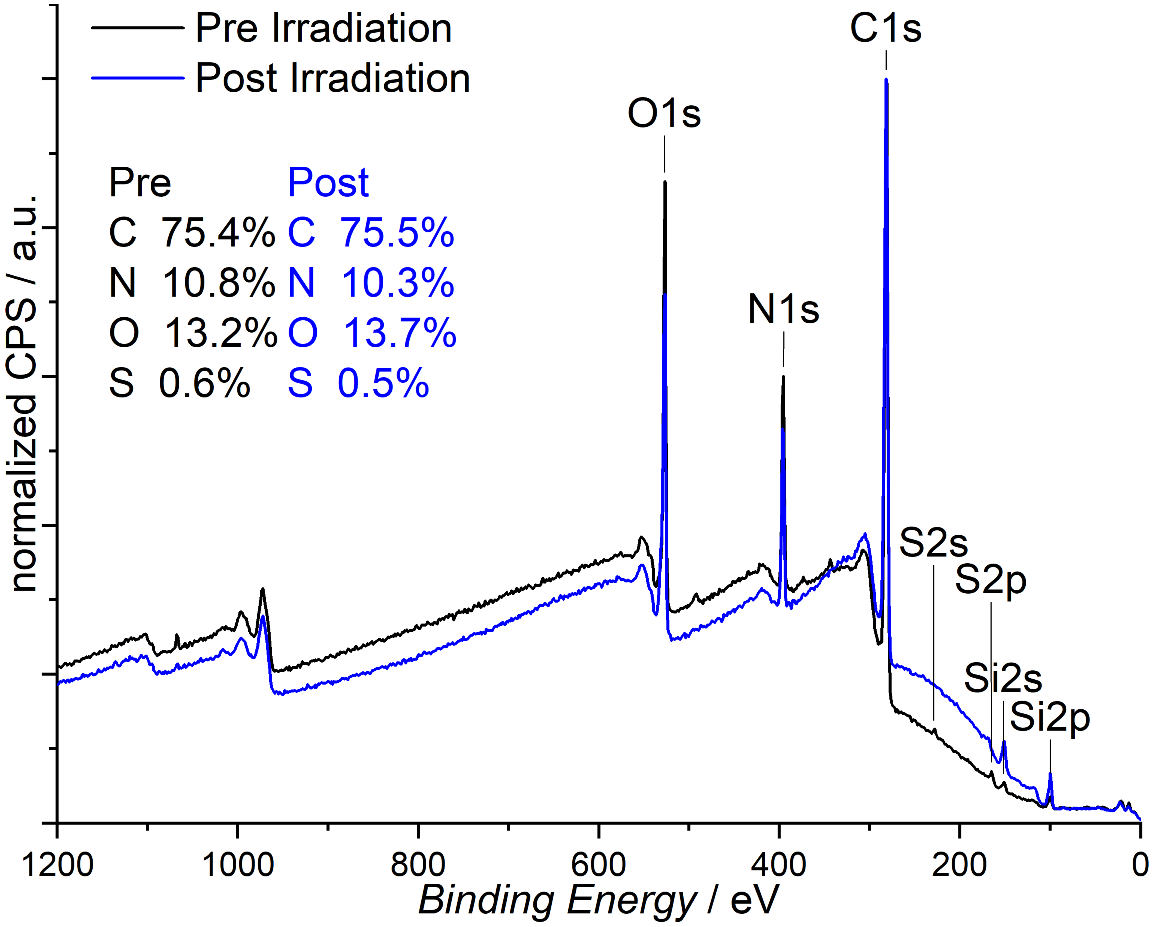

*Figure S34: XPS of an unstructured P(DMA-co-**M**) brush before and after photolysis.*

Patterned polymer brush photodegradation

Patterned degradation experiments were performed in water (main manuscript), but additionally conducted in toluene using the λ = 325 nm LED for 12 h at 3.5 cm distance.

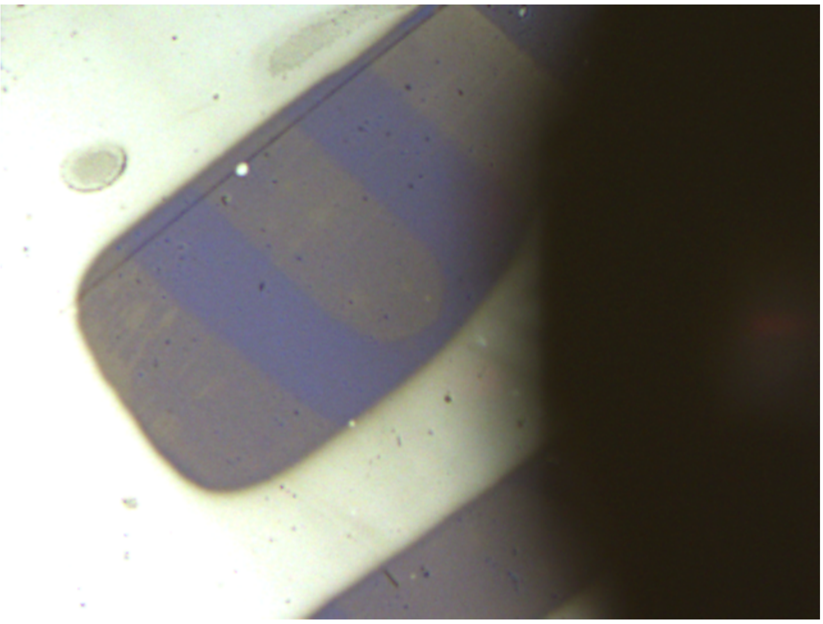

Figure S35: Microscope image of patterned brush with subsequent patterned degradation in Toluene.

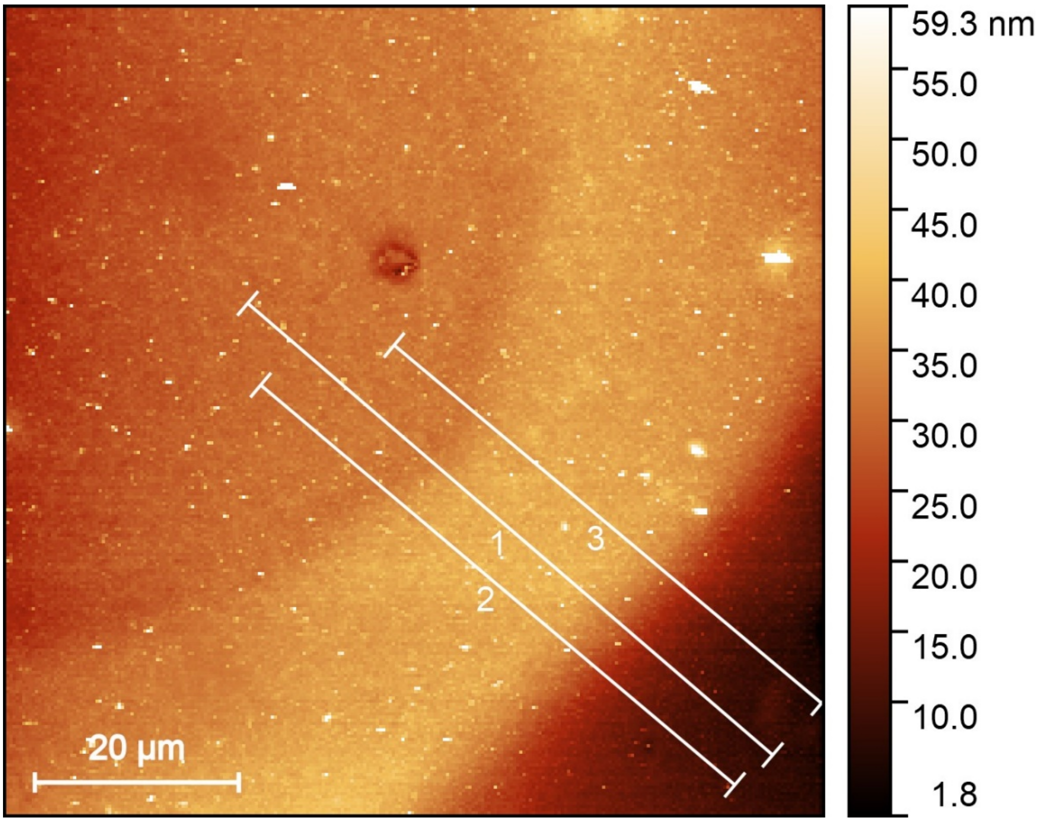

Figure S36: AFM image of patterned brush with subsequent patterned degradation in Toluene.

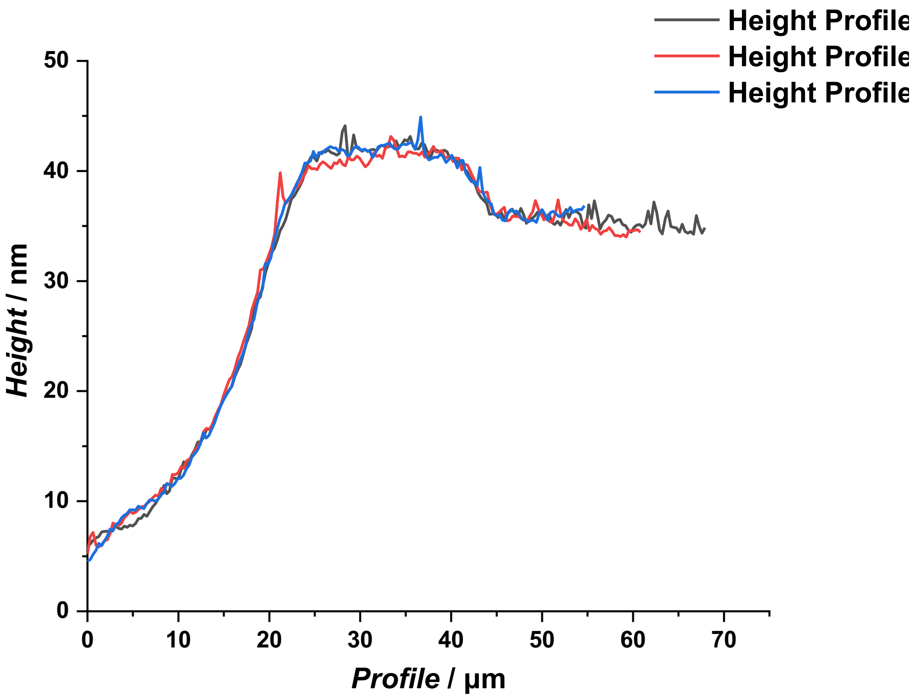

Figure S37: Height profile of functionalized silicon - pristine polymer brush interface and pristine brush - degraded polymer brush interface.

6.3 Polymer Brush Properties

6.3.1 Contact angle measurements

2 polymer brushes were obtained in parallel from identical stock solutions using standard conditions outlined in chapter 5.3. One polymer brush was subsequently degraded by irradiating the polymer brush for 30 min with UVB light in water. The other polymer brush was kept in the dark.

Contact angle measurements of both polymer brushes were performed. The average angle of three measurements on both sides of the drop was 70.97° for the pristine polymer brush and 70.76° after photodegradation. The following images show the water drops on the pristine and degraded polymer brush.

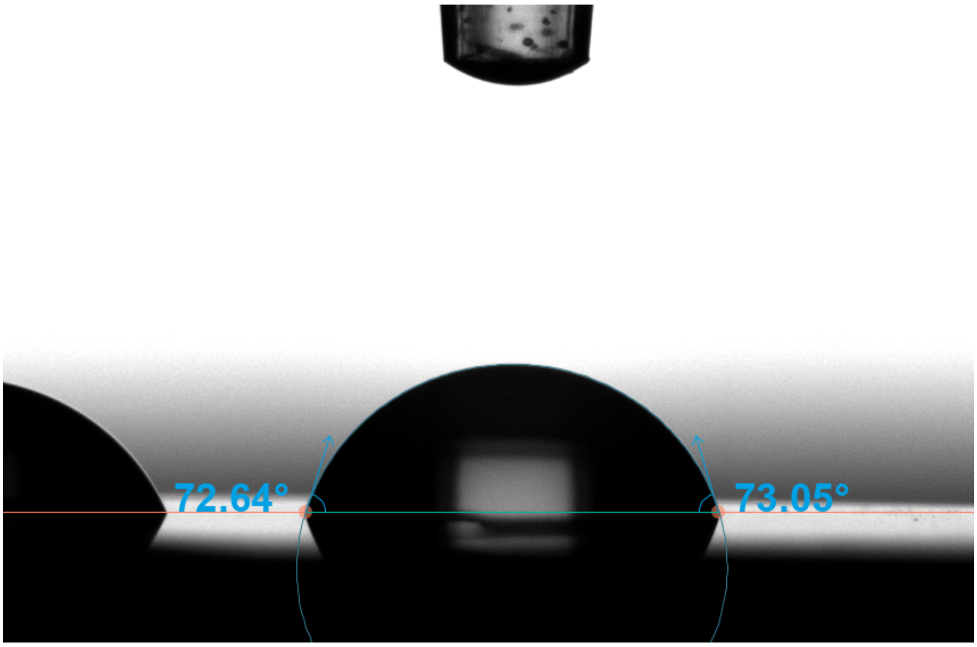

Figure S38: Drop of water on pristine polymer brush on small Si-wafer (10 x 10 mm).

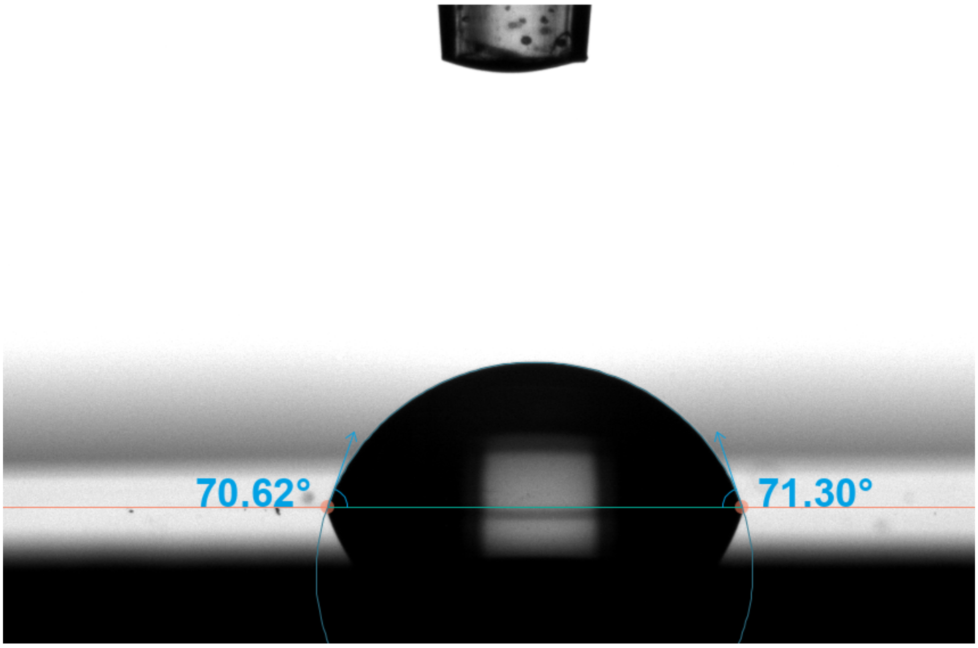

Figure S39: Drop of water on a degraded polymer brush on small Si-wafer (10 x 10 mm).

6.3.2 Adhesion measurements

The same polymer brushes used for contact angle measurements in 6.3.1 were used for adhesion measurements via AFM. The adhesion force distributions for both polymer brushes are overlayed in the following graph and do not show any significant difference indicating no significant change in hydrophobicity upon polymer brush degradation.

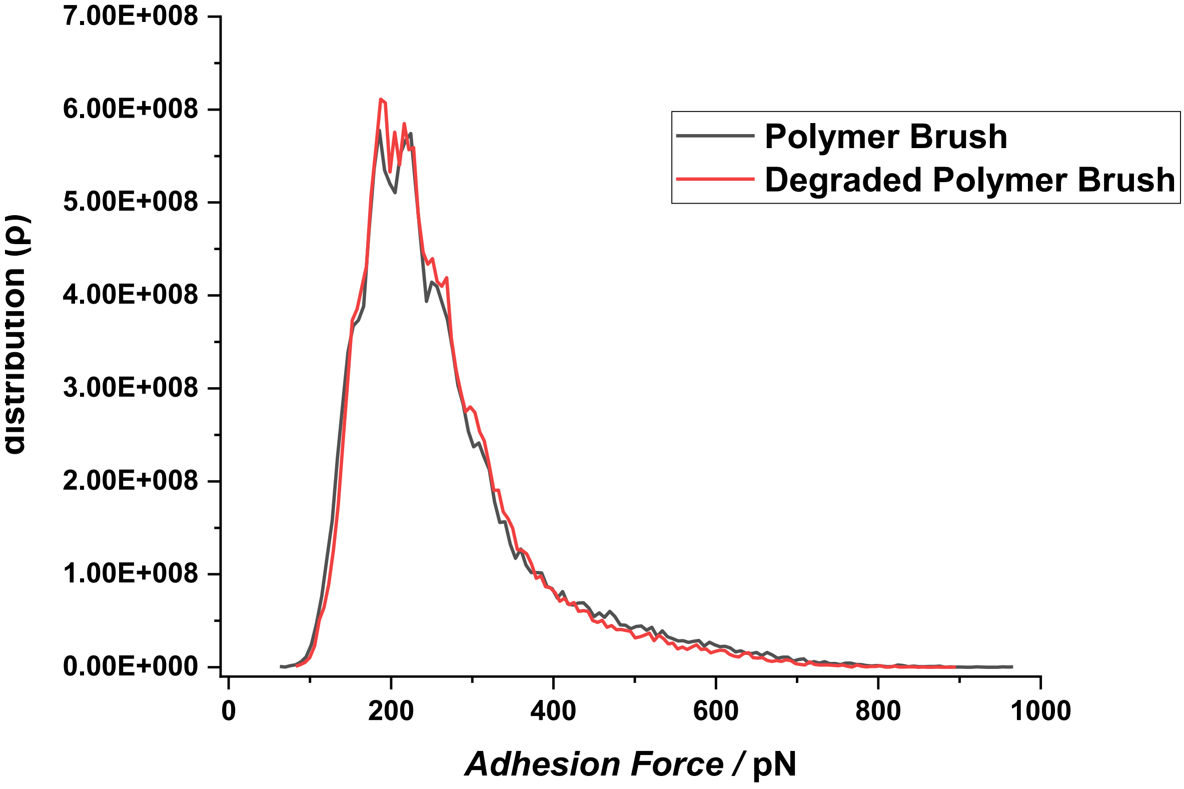

*Figure S40: Overlay of adhesion force distributions measured via AFM for a non-patterned pristine P(DMA-co-**M**) polymer brush and non-patterned degraded polymer brush.*

7 Characterization of large wafers (d = 10 cm)

NR measurements in the dry state of P(DMA-co-***M***) brush*es* were carried out before and after UV irradiation are shown in the following:

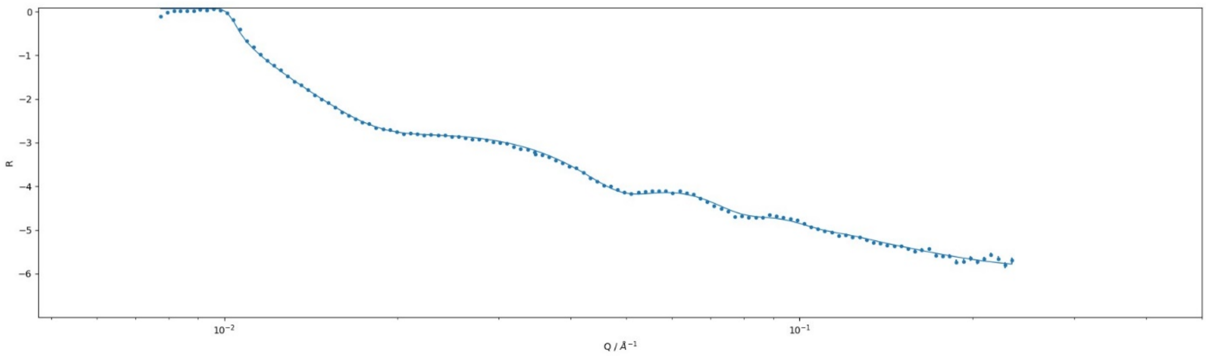

Figure S41: Reflectivity of P(DMA-co-**M**) brushes before UV irradiation.

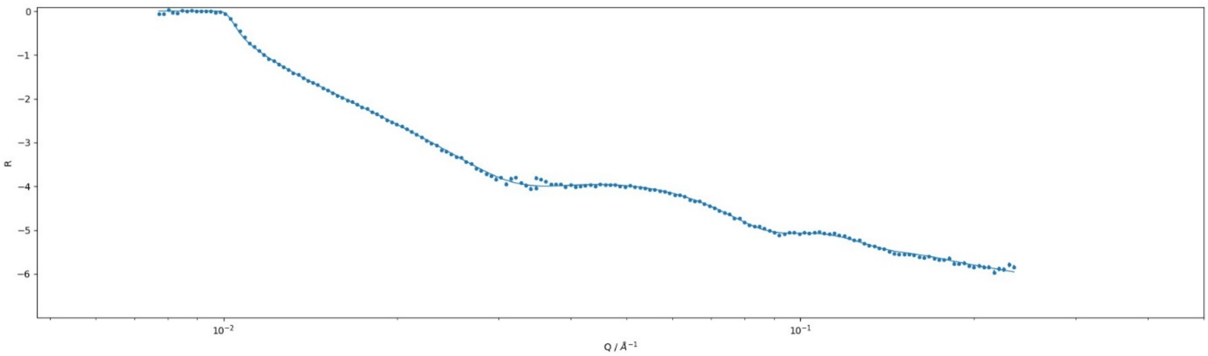

Figure S42: Reflectivity of P(DMA-co-**M**) brushes after UV irradiation *with LED centred at 325 nm*.

For NR measurements, a PDMA wafer was prepared and characterized via NR measurements and ellipsometry mapping:

<u>DMA homopolymer brush</u>

To confirm the polymer degradation being a direct result of the functional monomer **M** a homopolymer brush of PDMA was prepared. The wafer was characterized and showed a polymer film thickness of 175 – 225 Å (17.5 – 22.5 nm) according to Ellipsometry and 183.4 Å (18.34 nm) with a roughness of 20.1 Å (2.01 nm) according to NR before irradiation. After irradiation with 325 nm for 8 h the polymer film thickness was unchanged according to Ellipsometry.

*** During the measurements at ANSTO, the acquisition details including the density of datapoints as well as the scanned area for the ellipsometry maps were increased. Thus, the first measurements and the resulting maps show a smaller area on the sample with less datapoints.***

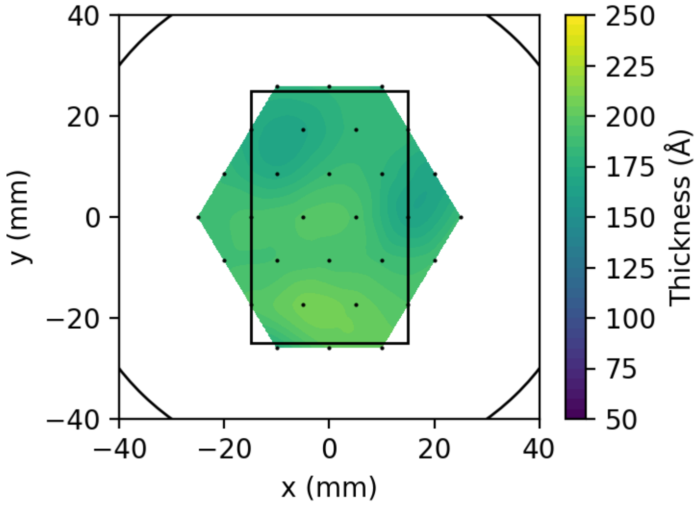

Figure S43: Dry film thickness map of PDMA brush.

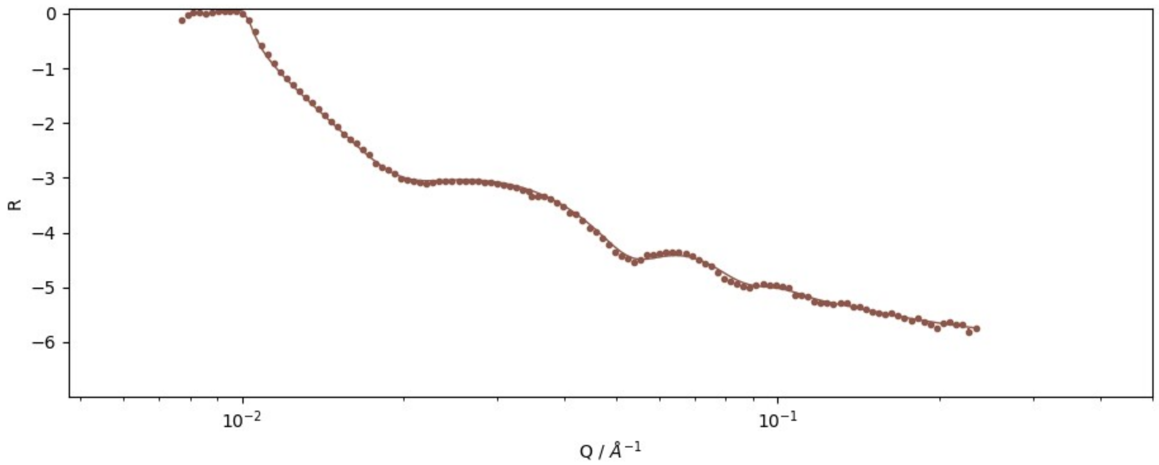

Figure S44: Reflectivity of PDMA brush before irradiation.

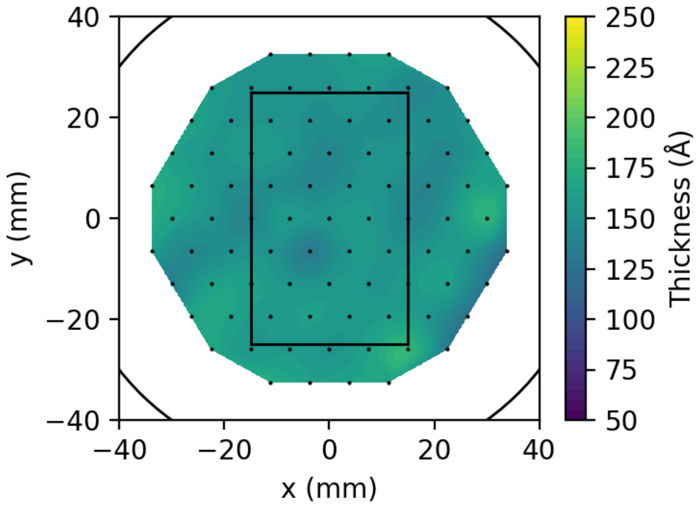

Figure S45: Dry film thickness map of PDMA brush after irradiation with an LED centred at 325 nm.

8 Fitting Liquid Cell Neutron Reflectometry Data

The P(DMA-*co*-**M**) brush wafer was analyzed in the swollen state in D_2O, using the approach by Gresham et al.[5,6] This process requires that the amount of grafted polymer be used as a model parameter. During irradiation this amount decreases. We therefore linearly decreased the amount of polymer in line with the linear trend observed in the ellipsometry measurements. The overlay of the fit and the raw data for all relevant time points is shown here:

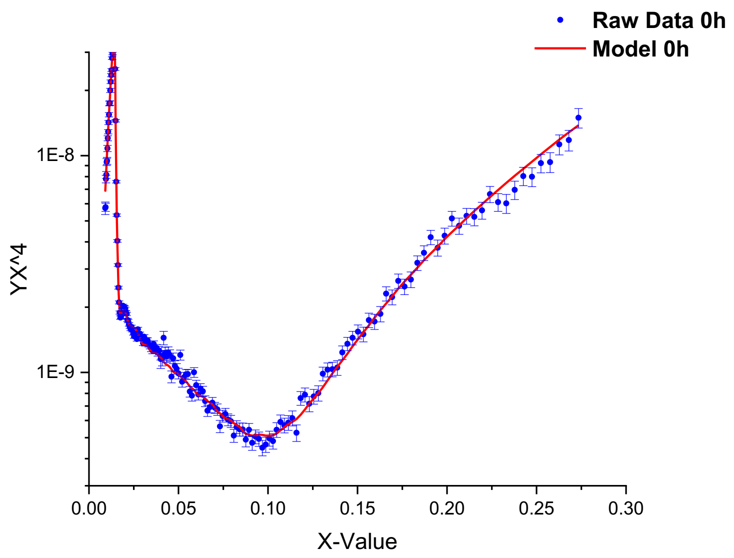

Figure S46: Overlay of raw data and fitted model for NR liquid cell measurement after 0 h irradiation.

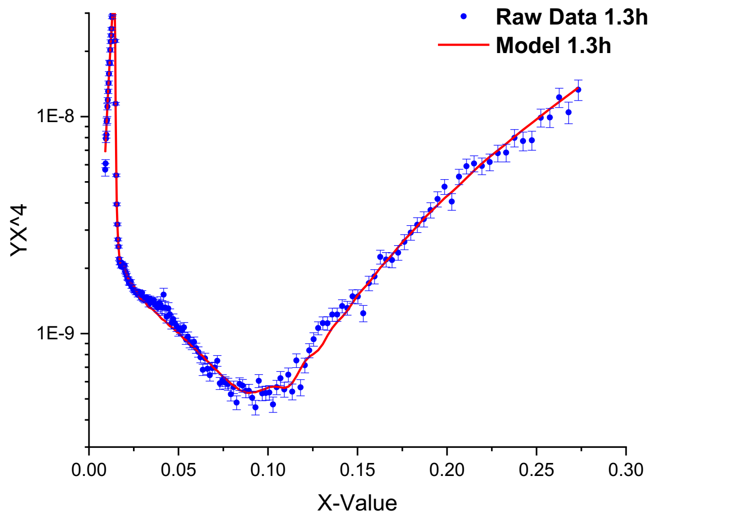

Figure S47: Overlay of raw data and fitted model for NR liquid cell measurement after 1.3 h irradiation.

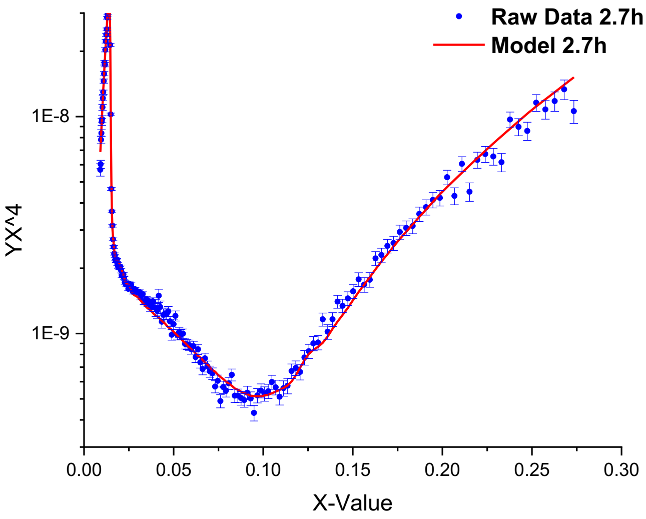

Figure S48: Overlay of raw data and fitted model for NR liquid cell measurement after 2.7 h irradiation.

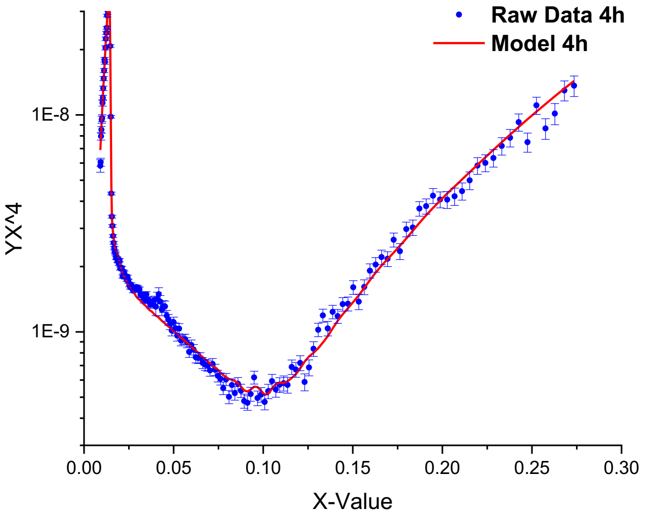

Figure S49: Overlay of raw data and fitted model for NR liquid cell measurement after 4 h irradiation.

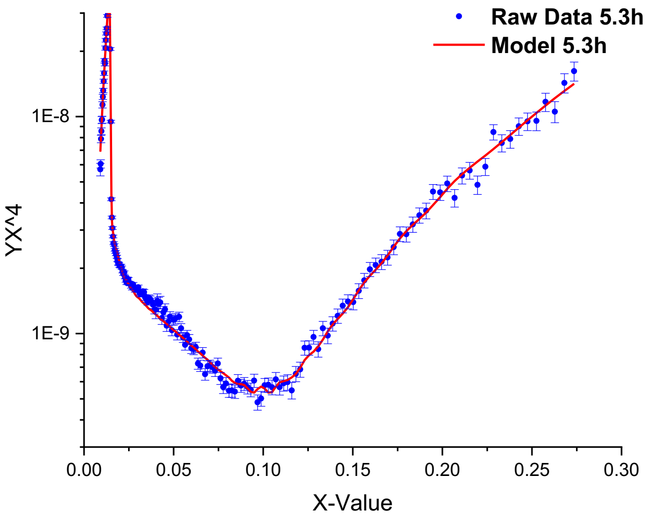

Figure S50: Overlay of raw data and fitted model for NR liquid cell measurement after 5.3 h irradiation.

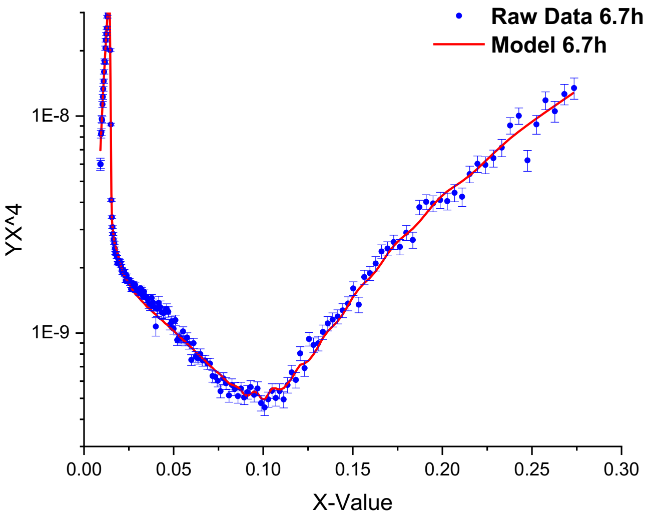

Figure S51: Overlay of raw data and fitted model for NR liquid cell measurement after 6.7 h irradiation.

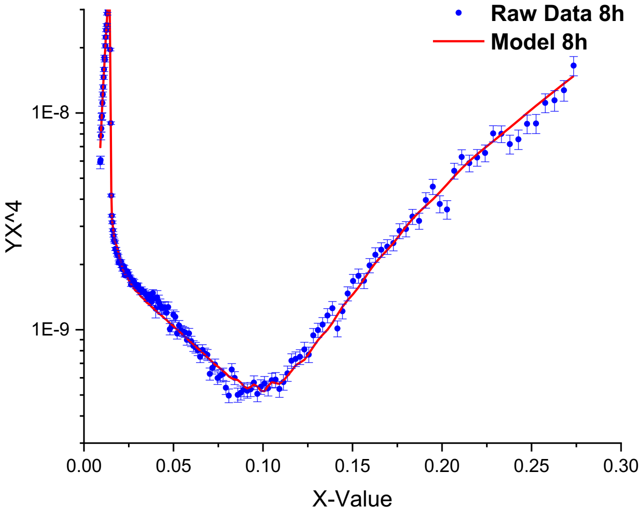

Figure S52: Overlay of raw data and fitted model for NR liquid cell measurement after 8 h irradiation.

The quality of the fit was further confirmed by the ratio of $\frac{\chi^2}{n}$ with:

$$\chi^2 = \sum \frac{(y_{i,calc} - y_{i,obs})}{\sigma_{i,obs}^2}$$

$$y_{i,obs}: observed\ data$$

$$\sigma_{i,obs}: uncertainty\ in\ observed\ data$$

$$y_{i,calc}: calculated\ model$$

Irradiation time [h] / Dataset	$\frac{\chi^2}{n}$
0	2.66
1.3	2.58
2.7	3.01
4	3.80
5.3	3.45
6.7	3.67
8	3.14

Table S2: $\frac{\chi^2}{n}$ values for all fitted datasets.

9 Extracted Ion Chromatograms of Degraded Polymer Brushes

To analyze the polymer detached from the surface, SEC-MS measurements were conducted. Eight polymer brush-coated wafers containing monomer **M** (total surface area ~6 cm²) were subjected to UVB irradiation (λ = 313 nm, distance 3 cm, 2 h) in the presence of a thin water layer. The detached polymer was collected by washing the wafers and covering glass slides with THF, followed by concentration via rotary evaporation and freeze-drying, and subsequent redissolution in THF for analysis.

Due to the low amount of recovered material, no signals clearly above baseline were observed in conventional RI and UV detectors. Therefore, mass spectrometric detection was evaluated in extracted ion chromatogram (XIC) mode. XIC traces were generated by selecting narrow m/z windows (±1.5–2 *m/z*) centered around calculated m/z values corresponding to coumarin-terminated oligomers with varying numbers of repeat units (N), based on simulated isotopic distributions.

Signals were observed at retention times between 16–20 min for selected ions. Comparison with a blank sample (THF only) showed no corresponding features above baseline within the same *m/z* window. It should be noted that blank comparison was only feasible for triply charged species, as the blank measurement was acquired over a limited *m/z* scan range (upper limit *m/z* = 2200). Due to an overall low signal intensity, no resolved isotopic patterns could be obtained.

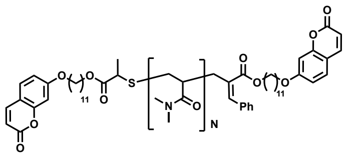

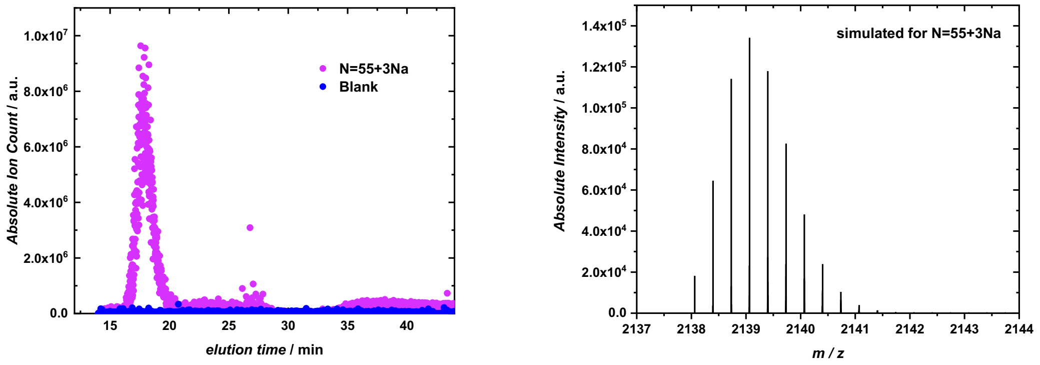

Figure S53: XIC traces of the degraded polymer solution (left) and pure THF (right) recorded for m/z = 3138–3141, targeting the calculated m/z range of a coumarin-terminated oligomer with N = 55 repeat units in the triply sodiated state, based on its simulated isotopic distribution (right).

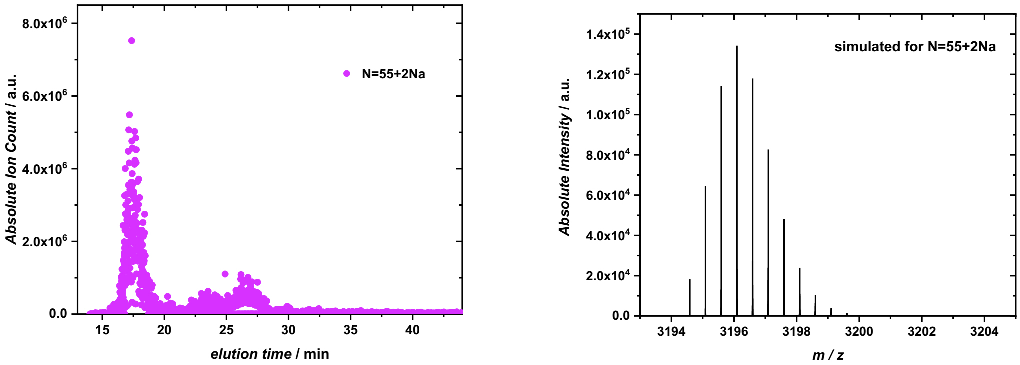

Figure S54: XIC traces of the degraded polymer solution (left) for m/z = 3194–3198, targeting the calculated m/z range of a coumarin-terminated oligomer with N = 55 repeat units in the doubly sodiated state, based on its simulated isotopic distribution (right).

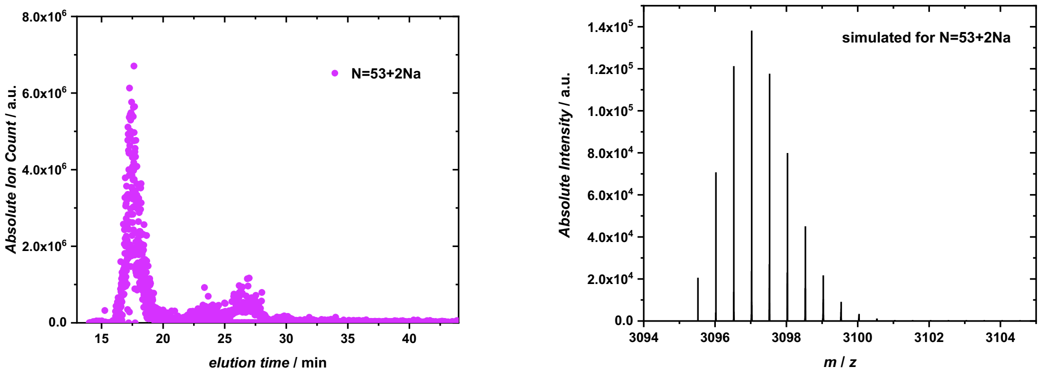

Figure S55: XIC traces of the degraded polymer solution (left) for m/z = 3095–3099, targeting the calculated m/z range of a coumarin-terminated oligomer with N = 53 repeat units in the doubly sodiated state, based on its simulated isotopic distribution (right).

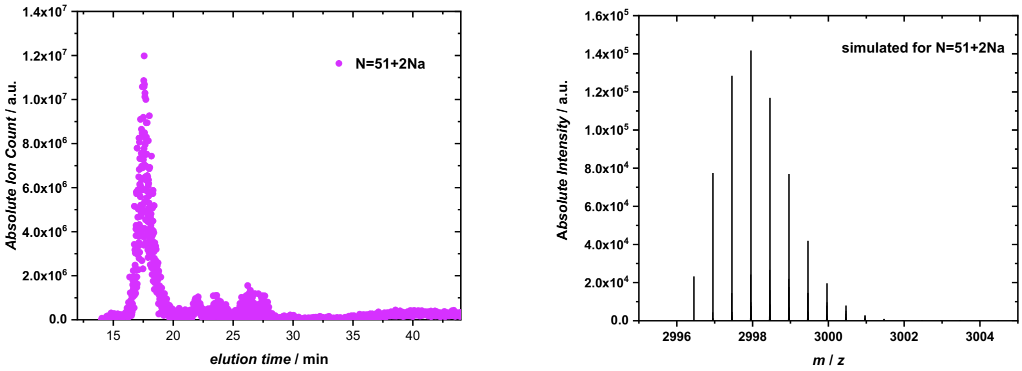

Figure S56: XIC traces of the degraded polymer solution (left) for m/z = 2996–3000, targeting the calculated m/z range of a coumarin-terminated oligomer with N = 51 repeat units in the doubly sodiated state, based on its simulated isotopic distribution (right).

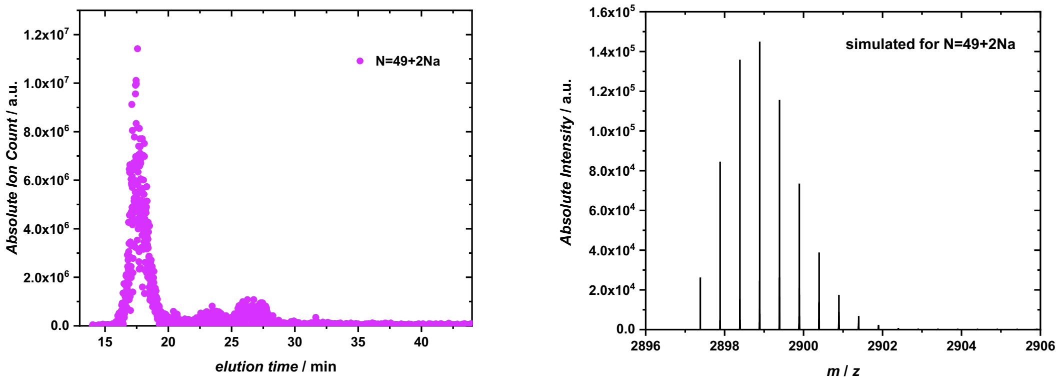

Figure S57: XIC traces of the degraded polymer solution (left) for m/z = 2897–2901, targeting the calculated m/z range of a coumarin-terminated oligomer with N = 49 repeat units in the doubly sodiated state, based on its simulated isotopic distribution (right).

10 Time-Dependent Conversions of DMA and M’

To probe the relative reactivity of DMA and M’, a PET-RAFT copolymerization of DMA with 0.2 mol% M’ was carried out in DMSO-d_6 following the procedure described in Section 5.3, employing an LED plate setup with increased light output. Monomer conversion was monitored by ^1H NMR spectroscopy using the vinyl proton signals of M’ (δ ≈ 5.91 ppm) and DMA (δ ≈ 5.62 ppm), alongside the appearance of the PDMA backbone CH_2 resonances in the range δ = 1.93–1.

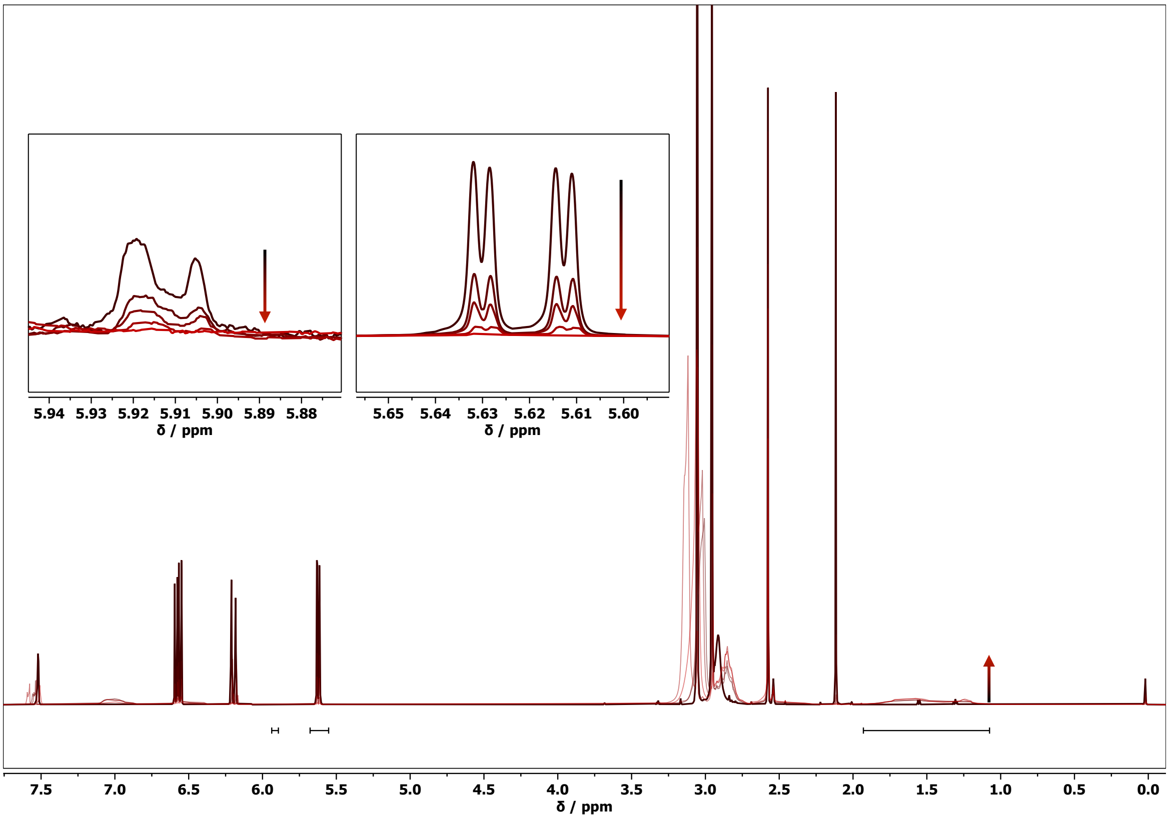

Figure S58: ^1H-NMR of a PET-RAFT copolymerization of DMA and M’ (0.2%) measured in a mixture of DMSO-d_6 and $CDCl_3$ after 90, 180, 360 and 540 min.

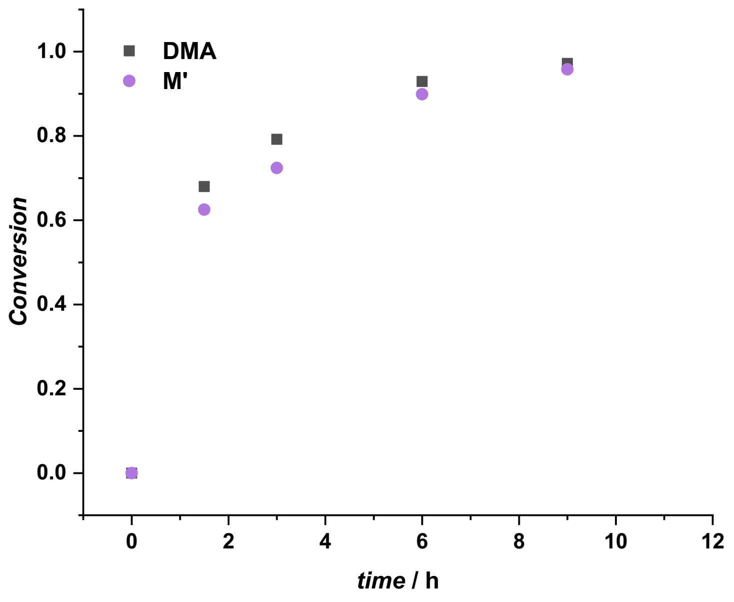

*Figure S59:Conversion of **M'** (purple circles) and DMA (black squares) during PET RAFT polymerization in solution according to ^{1}H-NMR.*

11 Literature

[1] H. Robertson, I. J. Gresham, S. W. Prescott, G. B. Webber, E. J. Wanless, A. Nelson, *SoftwareX* **2022**, *20*, 101225.

[2] P. T. Do, B. L. J. Poad, H. Frisch, *Angewandte Chemie International Edition* **2023**, *62*, DOI 10.1002/anie.202213511.

[3] R. A. Olson, J. S. Levi, G. M. Scheutz, J. J. Lessard, C. A. Figg, M. N. Kamat, K. B. Basso, B. S. Sumerlin, *Macromolecules* **2021**, *54*, 4880–4888.

[4] H. Foster, M. H. Stenzel, R. Chapman, *Macromolecules* **2022**, *55*, 5938–5945.

[5] I. J. Gresham, T. J. Murdoch, E. C. Johnson, H. Robertson, G. B. Webber, E. J. Wanless, S. W. Prescott, A. R. J. J. Nelson, *J Appl Crystallogr* **2021**, *54*, 739–750.

[6] A. J. Nelson, S. Prescott, *J Appl Crystallogr* **2019**, *52*, 193–200.
